# Thieno[3,2-*d*]pyrimidines in Anticancer Drug Discovery: Recent Advances in Drug Design and Molecular Targets

**DOI:** 10.3390/ijms27167457

**Published:** 2026-08-20

**Authors:** Anvarjon Buronov, Shukhrat Gaybullaev, Zarifa Murtazaeva, Feruza Ruzieva, Zohidjon Khushnazarov, Davron Turgunov, Azizbek Nasrullaev, Rustamkhon Kuryazov, Yuldash Takhirov, Firdavsi Tursunov, Temur Kushatov, Dilshod Dushamov, Shavkat Matmuratov, Nilufar Nurullaeva, Aziza Shodikulova, Kakhor Khalikov, Dilafruz Kholmurodova, Sodik Numonov, Chao Niu, Yuanyuan Ji, Jiangyu Zhao, Zhishen Ge, Khurshed Bozorov

**Affiliations:** 1 Department of Medicinal Chemistry, Institute of Biochemistry, Samarkand State University, University Blvd. 15, Samarkand 140104, Uzbekistan; anvarjon4488@gmail.com (A.B.);; 2 Department of Chemistry, Urgench State University named after Abu Rayhan Biruni, Kh. Olimjon st. 14, Urgench 220100, Uzbekistan; 3 Samarkand State Medical University, Amir Temur st. 18, Samarkand 140100, Uzbekistan; 4 Scientific and Practical Center of Immunology, Allergology and Human Genomics, Samarkand State Medical University, Makhdūm-i Aʿẓam st. 18, Samarkand 140104, Uzbekistan; 5 Research Institution “Chinese-Tajik Innovation Center for Natural Products”, National Academy of Sciences of Tajikistan, Dushanbe 734063, Tajikistan; 6 State Key Laboratory Basis of Xinjiang Indigenous Medicinal Plants Resource Utilization, Xinjiang Technical Institute of Physics and Chemistry, Chinese Academy of Sciences, South Beijing Rd 40-1, Urumqi 830011, China; 7 Department of Geriatric General Surgery, The Second Affiliated Hospital, Xi’an Jiaotong University, Xi’an 710004, China; 8 School of Chemistry, State Key Laboratory of Fluorine & Nitrogen Chemicals, Institute of New Concept Sensors and Molecular Materials (INCSMM), Engineering Research Center of Energy Storage Materials and Devices, Ministry of Education, Xi’an Key Laboratory of Sustainable Polymer Materials, Xi’an Jiaotong University, Xi’an 710049, China

**Keywords:** anticancer agents, CDK inhibitors, dual-target inhibitors, EGFR inhibitors, olmutinib, pictilisib, PI3K/mTOR inhibitors, structure–activity relationship, thieno [3,2-*d*]pyrimidines, thiophene

## Abstract

The thieno[3,2-*d*]pyrimidine scaffolds have emerged as an important class of heterocycles in anticancer drug discovery, with clinically advanced drugs olmutinib and pictilisib highlighting their therapeutic potential. This review presents thieno[3,2-*d*]pyrimidine-containing anticancer agents reported between January 2008 and August 2025, focusing on synthetic methodologies, anticancer-related biological activities, and structure–activity relationships. Thieno[3,2-*d*]pyrimidine derivatives have been investigated as inhibitors of numerous cancer-related targets, including EGFR, PI3K/mTOR, CDKs, JAK, VEGFR, HDAC, ATR, and other oncogenic proteins. This review also summarizes thieno[3,2-*d*]pyrimidine scaffolds with anticancer activity, with particular emphasis on the design and synthesis of lead compounds, molecular hybridization strategies, and recent advances in this area. Synthetic pathways for lead compounds are systematically presented and discussed, along with pharmacophoric features. In addition, detailed structure–activity relationship analyses are provided to highlight the influence of heterocyclic fusion, linker optimization, hydrogen-bonding motifs, electronic effects, hydrophobic fragments, and the introduction of hybrid scaffolds on antiproliferative potency, kinase inhibition, selectivity, and multitarget activity. In addition, this review demonstrates the significant potential of thieno[3,2-*d*]pyrimidine-based scaffolds as a privileged platform for the development of next-generation targeted anticancer agents and offers valuable guidance for future medicinal chemistry research.

## 1. Introduction

Cancer remains one of the leading causes of death worldwide [1,2]. According to the World Health Organization (WHO), it causes about 10 million deaths each year [3]. The global cancer burden is expected to grow due to aging populations, changing lifestyles, and environmental factors [4,5,6,7]. There has been progress in surgery, radiotherapy, and immunotherapy [8,9,10]. Still, chemotherapy remains essential for treating most cancers [11,12]. However, the effectiveness of current anticancer drugs is often limited [13,14,15]. Issues include multidrug resistance, toxic side effects at higher doses, and poor specificity [16]. These problems highlight the urgent need for chemotherapeutic drugs that are more potent, selective, and safe. In the past twenty years, drug discovery has increasingly focused on small molecules that block cancer-specific pathways [17,18,19]. This shift has advanced the field of precision oncology [20].

Heterocyclic scaffolds play a crucial role in medicinal chemistry, particularly in the development of anticancer drugs [21,22,23,24]. Nitrogen-containing heterocycles [25,26], such as pyridines, pyrimidines, purines, quinazolines, and triazines, appear in many FDA-approved drugs [27,28,29]. These compounds offer diverse pharmacological effects, structural flexibility, and the ability to interact with biological macromolecules [30]. Nitrogen-rich systems are often used to create anticancer agents that target kinases, tubulin, topoisomerases, and epigenetic regulators. Many scaffolds act as ATP mimetics in kinase inhibitors, which explains their frequent appearance in approved drugs. Over the last decade, our research team has focused on synthesizing and testing nitrogen-containing heterocycles with fused or partially saturated rings [31,32,33,34,35,36,37,38,39,40,41,42,43]. These scaffolds often mimic natural products, such as alkaloids, and show promising pharmacological effects. We have investigated their anticancer potential by modifying oxazole [44], pyrrole [45], imidazole [46,47], thiophene [48,49], and furan [50,51,52] cores to improve target-specific binding and activity. In addition, five-membered pyrazole [53,54] fragments are also being studied as thienopyrimidine analogs for their anticancer potential [23,33]. This work provides a solid foundation for our current research on thienopyrimidine-based compounds.

**Figure 1:**
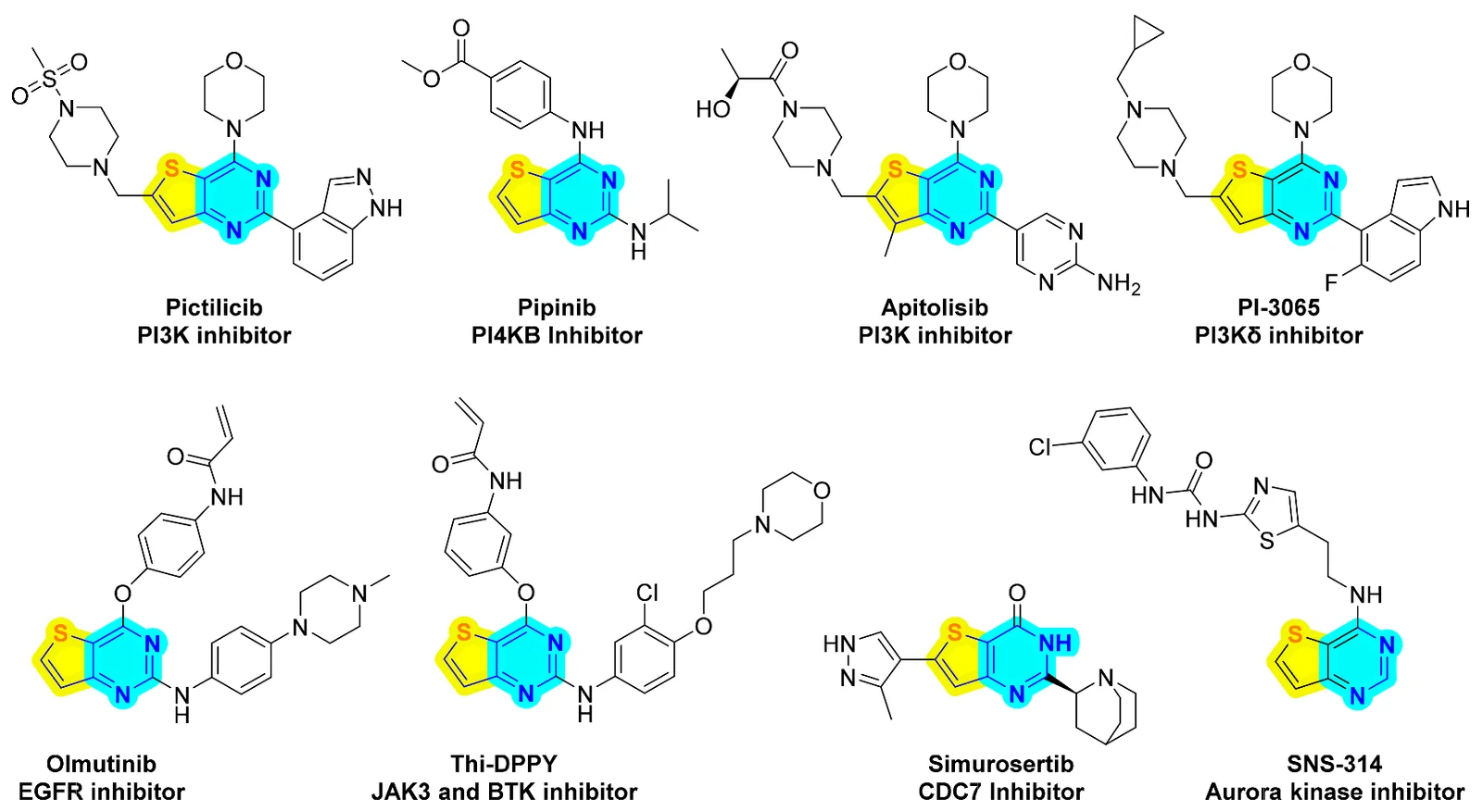
Structures of selected thieno[3,2-*d*]pyrimidine-based anticancer drugs.

The thieno[3,2-*d*]pyrimidine core has become a valuable tool in cancer research [55,56,57]. This fused bicyclic heterocycle consists of a thiophene ring fused with a pyrimidine ring, combining electron-rich and electron-deficient features. Its structural similarity to purines allows it to bind to the ATP-binding sites of various kinases. The thiophene piece also increases lipophilicity and aids cell penetration. Over the last fifteen years, numerous thieno[3,2-*d*]pyrimidine derivatives have demonstrated strong anticancer activity through diverse mechanisms [58,59,60,61]. These include kinase inhibition (e.g., EGFR, PI3K, CDK, and JAK), tubulin disruption, cell-cycle arrest, apoptosis, and epigenetic modulation. Some derivatives have achieved nanomolar IC_50_ values in vitro and reduced tumor growth in vivo. These results make this scaffold a key platform for designing multi-target drugs [62]. For example, several compounds based on the thieno[3,2-*d*]pyrimidine structure have reached preclinical or clinical testing as targeted anticancer agents (Figure 1). Notable examples include pictilisib [63,64,65,66], apitolisib (GDC-0980) [57,67,68,69], PI-3065 [70,71,72], and pipinib [73], which are strong inhibitors of the PI3K pathway and show promising antitumor effects by affecting the PI3K/AKT/mTOR signaling pathway. Olmutinib also uses this scaffold and has been studied as an EGFR inhibitor [74], improving binding and selectivity for mutant EGFR forms. Simurosertib (TAK-931) shares this core and acts as a selective CDC7 inhibitor [75,76], disrupting the cell cycle. Thi-DPPY has shown JAK-inhibitory activity [77], and SNS-314 has been identified as an Aurora kinase inhibitor [78,79,80]. These cases demonstrate the flexibility of the thieno[3,2-*d*]pyrimidine core in the development of kinase-targeted anticancer drugs.

This review compiles and examines literature from January 2008 to August 2025. It focuses on thieno[3,2-*d*]pyrimidine-based lead compounds with proven anticancer activity and experimental support. Only studies with biological results, such as IC_50_ values, apoptosis assays, kinase inhibition, and in vivo efficacy, are included. Studies without direct anticancer evaluations or focusing only on synthesis are excluded. The selected lead compounds are grouped by molecular targets, including single-target agents (e.g., EGFR, PI3K, and CDK) and multi-target inhibitors. For each compound, this review provides details on synthesis, biological potency, mechanisms, and structure–activity relationships (SAR). This approach offers a comprehensive view of their significance and prospects.

## 2. Thieno[3,2-*d*]pyrimidines: Previous Reviews, Relation and Difference to Present Review

**Figure 2:**
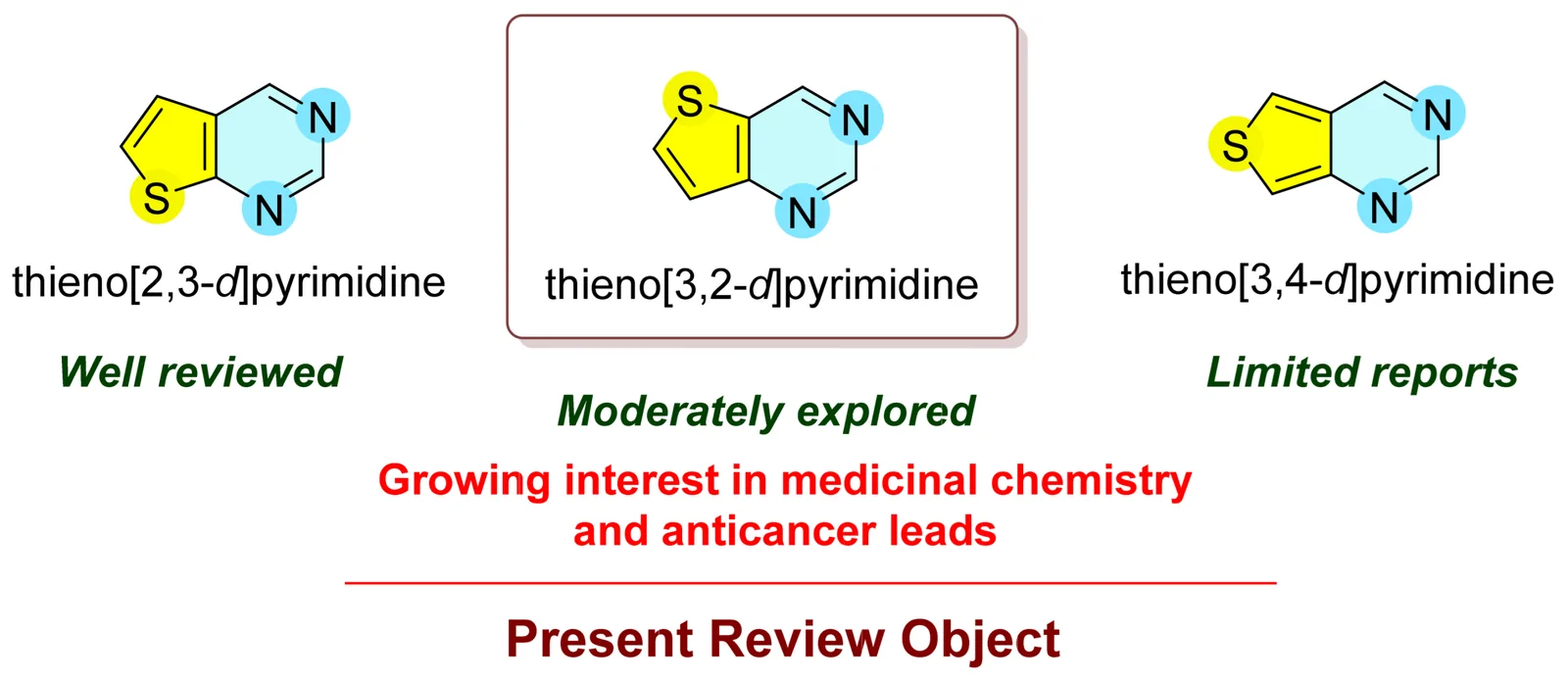
Comparative overview of thienopyrimidine scaffolds and the focus of the present review.

Thienopyrimidines are an important class of fused sulfur- and nitrogen-containing heterocycles formed through annulation of thiophene and pyrimidine rings. Depending on the fusion pathway, thienopyrimidines are generally classified into three principal structural isomers, namely thieno[2,3-*d*]pyrimidines, thieno[3,2-*d*]pyrimidines, and thieno[3,4-*d*]pyrimidines (Figure 2). Variations in ring fusion influence electronic distribution, heteroatom orientation, and molecular recognition properties, which may affect their synthetic behavior and biological activity profiles.

Among the three principal thienopyrimidine frameworks, thieno[3,2-*d*]pyrimidines hold an intermediate position in the literature. While thieno[2,3-*d*]pyrimidines have been extensively explored, thieno[3,4-*d*]-analogs remain comparatively less investigated. In addition, thieno[3,2-*d*]pyrimidines have attracted increasing attention in medicinal chemistry, particularly for anticancer drug discovery.

Several thienopyrimidine-titled reviews have been presented so far. However, most of them are dedicated to mixed isomers of thienopyrimidine scaffolds (i.e., reviewed [2,3-*d*] isomers or all three types) [81,82,83,84]. In 2024, Farag et al. [85] reviewed aryl urea-containing thienopyrimidines as anticancer agents targeting VEGFR kinases, focusing on reported literature during 2013–2023, SARs, and biological activity. It was target-oriented, not specifically about thieno[3,2-*d*]pyrimidines or broader anticancer potentials (Table 1). In 2025, Nadar et al. [86] reviewed thienopyrimidines, covering their synthesis and biological activities. It discussed all three major isomers and their anticancer, antimicrobial, and other diverse biological properties, also not specifically about thieno[3,2-*d*]pyrimidines. In 2017, Ghith et al. [87] reviewed thienopyrimidine-based scaffolds as kinase-targeting anticancer agents. The focus was on kinase inhibition and SARs, but not specifically on total synthesis of thieno[3,2-*d*]pyrimidine leads. In 2026, Rabeh et al. [88] summarized green synthetic approaches for thienopyrimidines from 2007 to 2025, discussing sustainable synthetic pathways for both thieno[2,3-*d*] and thieno[3,2-*d*] systems. The review was synthesis-oriented and did not specifically address the medicinal chemistry or anticancer properties of thieno[3,2-*d*]pyrimidines. In 2025, Liu et al. [89] discussed thienopyrimidines as scaffolds for kinase inhibitors with anticancer activity, covering their biological activity and SARs, including thieno[2,3-*d*]- and thieno[3,2-*d*]pyrimidines. The review was kinase-focused and not only dedicated analysis of anticancer thieno[3,2-*d*]pyrimidine derivatives. In 2023, Sayed et al. [90] reviewed synthetic approaches and anticancer activities of thienopyrimidines, mainly from the last nine years. Although both isomers of the thienopyrimidine scaffold were included, the discussion focused on the thieno[2,3-*d*] series, with limited coverage of thieno[3,2-*d*]pyrimidine analysis. In 2022, Lagardère et al. [91] reviewed thienopyrimidines as anti-infectives, covering all three isomers and their synthetic pathways and antimicrobial activities. However, it primarily focused on anti-infective applications and did not specifically address the anticancer properties of thieno[3,2-*d*]pyrimidines.

In 2021, Islam and Quadery [92] published a review specifically dedicated to thieno[3,2-*d*]pyrimidines. The review summarized synthetic strategies, therapeutic applications, patent literature, and SARs of thieno[3,2-*d*]pyrimidine derivatives across diverse biological applications. However, despite its scaffold-specific nature, the review was not primarily focused on anticancer leading compounds and their total synthetic pathways and included broader pharmacological applications.

Thus, we have focused only on the thieno[3,2-*d*]pyrimidine series because of its high use in medicinal chemistry, most commonly centered on the synthesis pathways of leading compounds, along with their SARs. The next subsection (PRISMA analysis) will explain the methodology for literature selection.

### Methodology for Literature Selection

**Figure 3:**
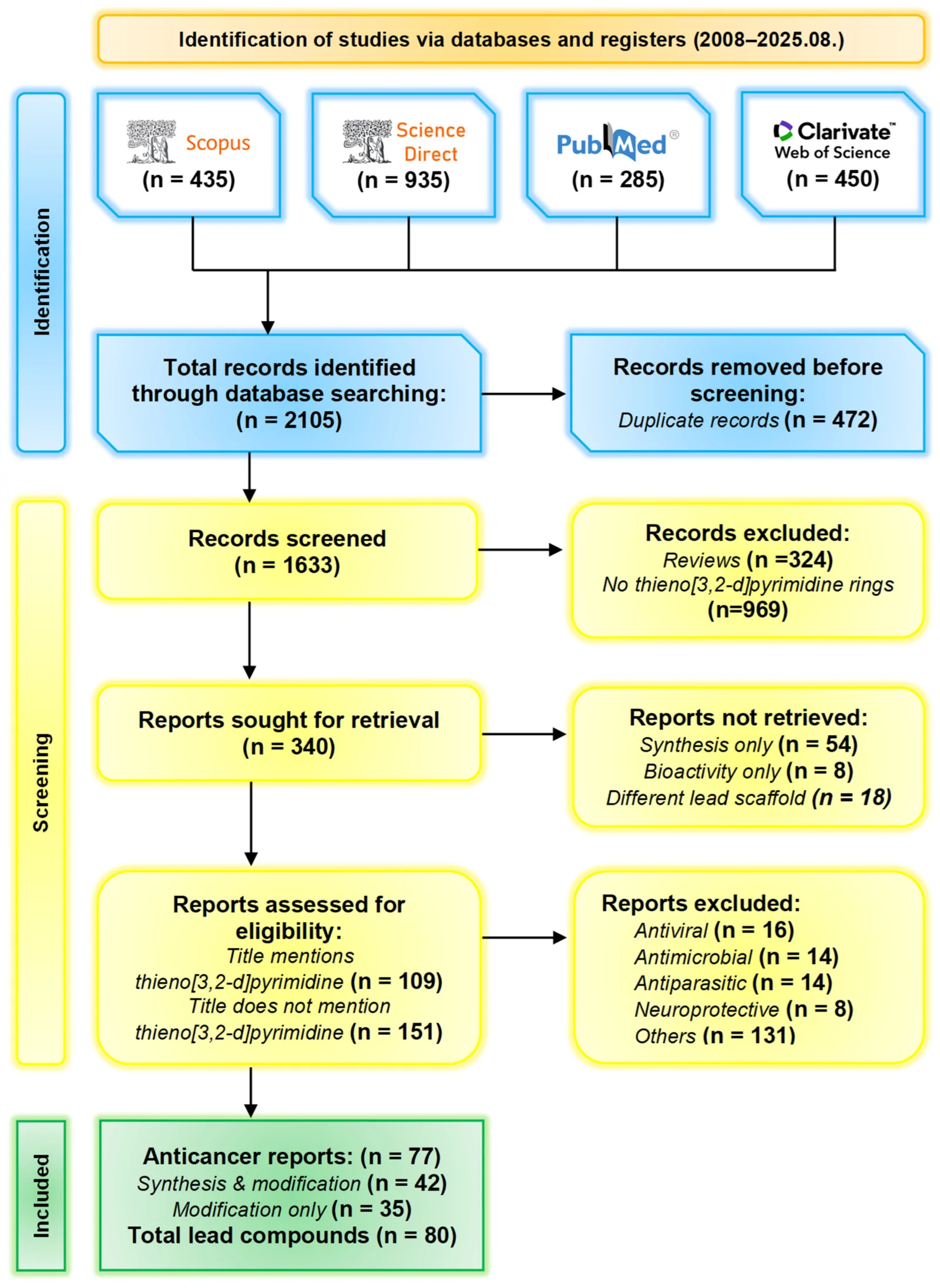
Literature selection of thieno[3,2-*d*]pyrimidine-based leading compounds in this review.

The current review was conducted in accordance with the PRISMA (Preferred Reporting Items for Systematic Reviews and Meta-Analyses) guidelines (Figure 3) [93]. These guidelines help ensure systematic and unbiased selection of relevant studies. The primary objective was to compile peer-reviewed articles detailing the synthesis and anticancer testing of thieno[3,2-*d*]pyrimidine-based lead compounds. Studies are needed to provide clear structural and biological data, including IC_50_ values, kinase inhibition assays, and apoptosis assays. The review included original research published from January 2008 to August 2025. Studies were retrieved from PubMed, Web of Science, Scopus, and ScienceDirect. The search used the following terms: “thieno[3,2-*d*]pyrimidine” and “anticancer” or “cytotoxicity”, as shown in Figure 3.

References were included if they met the following criteria. First, they involved the experimental synthesis of thieno[3,2-*d*]pyrimidine derivatives. Second, they featured at least one anticancer assay, such as MTT, SRB, flow cytometry, or kinase profiling. Third, they identified a lead compound based on potency, selectivity, or SAR optimization. Studies were excluded if they focused solely on synthetic methods without biological testing, examined unrelated pharmacological effects (e.g., antimicrobial or anti-inflammatory actions), or lacked structural data. Review articles, patents, and conference abstracts were also excluded from the analysis. More than 1600 articles were screened. Ultimately, 83 original research papers met all criteria and were selected.

Figure 3 (PRISMA chart) shows the literature selection process. Molecular targets, including EGFR [94,95], PI3K [96,97], CDK [98,99], tubulin [100], JAK [101], and mTOR [102], were classified in the chosen studies. Mechanistic profiles, including apoptosis induction, kinase inhibition, and cell-cycle arrest, were also utilized for classification. Total synthetic pathways, target binding, anticancer activity, and SAR were gathered for each lead compound. The obtained data were then organized into thematic subsections. This target-focused classification helps clarify the pharmacological significance and therapeutic prospects of thieno[3,2-*d*]pyrimidine leads.

## 3. Anticancer Properties of Thieno[3,2-*d*]pyrimidines

### 3.1. Antiproliferative Activity

**Scheme 1:**
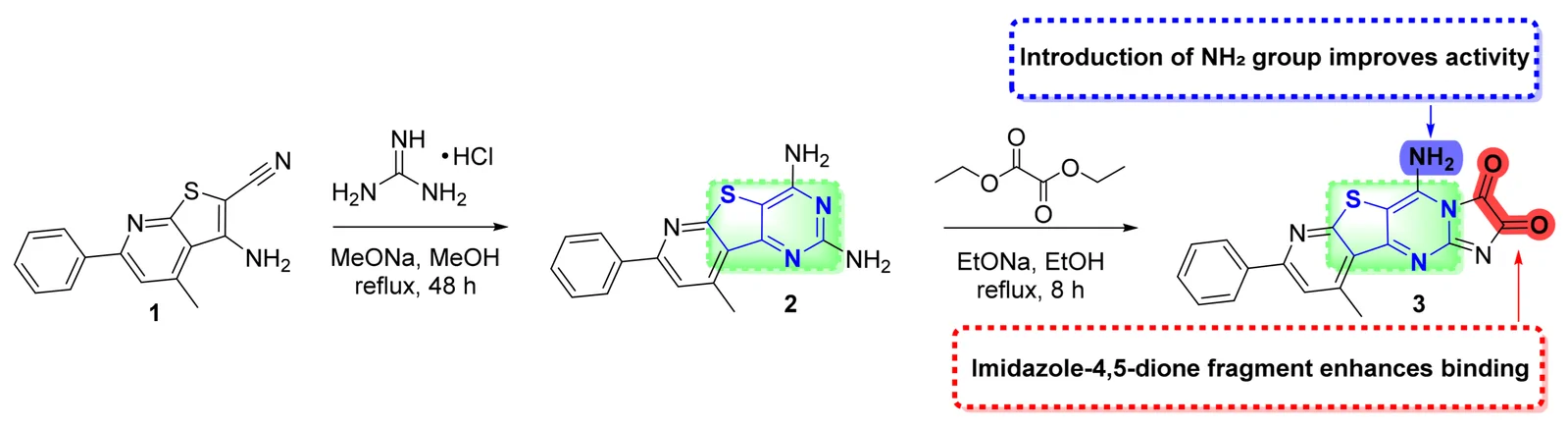
Structure and synthetic route of lead compound **3**.

Antiproliferation, or the inhibition of cancer cell growth and division, is a key part of current cancer treatments [103]. Heterocyclic scaffolds are important in this context because their varied structures enable targeted control of antiproliferative activity by interacting with specific molecular targets [104,105,106]. Thieno[3,2-*d*]pyrimidines, a notable group of heterocycles that combine thiophene and pyrimidine groups, show strong antiproliferative effects in many types of human cancer cell lines. Hassan et al. [107] synthesized a series of fused heterocycles based on a thieno[3,2-*d*]pyrimidine scaffold. Starting from 3-amino-4-methyl-6-phenylthieno[2,3-*b*]pyridine-2-carbonitrile, cyclization with guanidine hydrochloride, followed by reaction with diethyl oxalate, afforded compound **3** bearing an imidazole-4,5-dione ring. The reaction proceeded through stepwise nucleophilic attack with loss of ethanol (Scheme 1). Compound **3** showed more potent cytotoxicity than doxorubicin against HepG-2 (IC_50_ = 10.16 μM), but was less active on MCF-7 (IC_50_ = 32.7 μM). SAR of derivative **3** shows that the addition of an amino group increases antiproliferative activity by acting as a hydrogen-bond donor and facilitating interactions with the biological target, particularly in HepG-2 cells. Incorporating the imidazole-4,5-dione fragment also increases cytotoxicity by facilitating more effective ligand–target interactions and binding (Scheme 1).

**Scheme 2:**
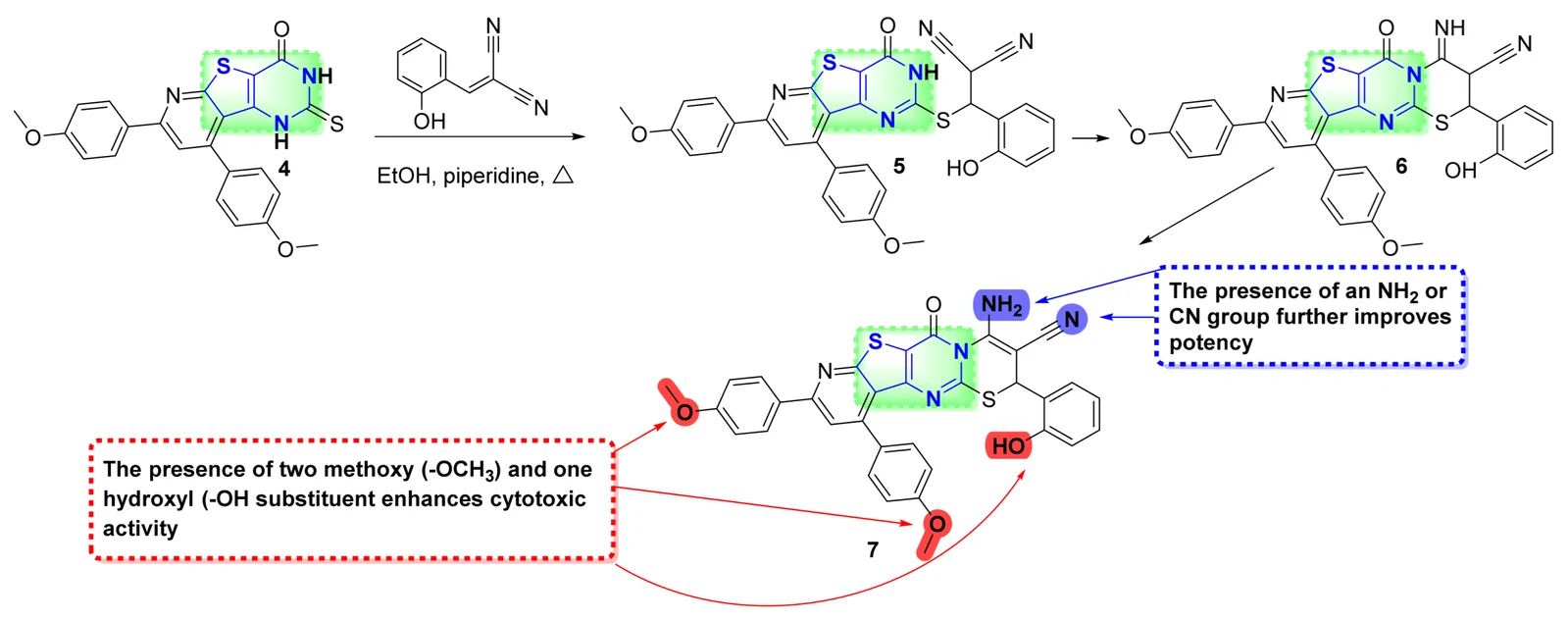
Structure and synthetic route of lead compound **7**.

Abbas and co-workers [108] synthesized a new tetracyclic pyrimidothiazine by reacting a thioxothienopyrimidine with an aromatic acceptor through a Michael addition in refluxing ethanol. The resulting intermediate underwent hydrogenation, followed by spontaneous intramolecular cyclization to afford compound **7** (Scheme 2). Lead derivative **7** was tested for antiproliferative activity and showed IC_50_ values of 6.16 ± 0.65 µM in MCF-7 cells and 7.13 ± 0.97 µM in HepG2 cells using the MTT assay. These results were close to those of the reference drugs doxorubicin and vinblastine, indicating that this scaffold may serve as a starting point for further optimization. SAR analysis of derivative **7** shows that the presence of hydroxyl and methoxy groups increases the antiproliferative activity of the compound in HepG2 cells. The hydroxyl group facilitates hydrogen bonding, while the amino and cyano groups enhance cytotoxicity by optimizing ligand–target interactions.

**Scheme 3:**
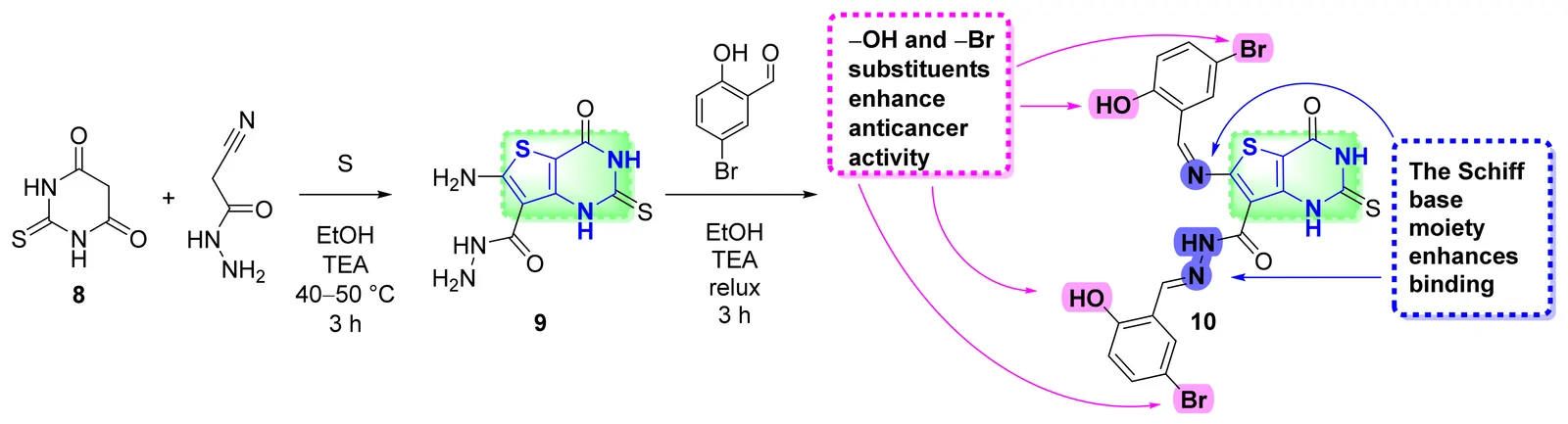
Structure and synthetic route of lead compound **10**.

A series of thieno[3,2-*d*]pyrimidine-7-carbohydrazide derivatives were synthesized by Aly et al. [109] via a Gewald-type cyclization, followed by Schiff base formation to obtain the lead compound **10** (Scheme 3). This compound exhibited selective cytotoxicity against Caco2 cells (IC_50_ = 0.73 µM), with minimal effect on normal fibroblasts (IC_50_ = 3.61 µM), resulting in a selectivity index of 5.8. The nanoscale La-complex of **10** further improved activity (IC_50_ = 0.61 µM), suggesting that heteroatoms (*O*, *N*, *S*) and metal coordination enhance its antitumor potential. SAR analysis of derivative **10** indicates that the presence of a phenolic hydroxyl group and a bromine atom on the aromatic ring increases the compound’s anticancer activity. The addition of the Schiff base moiety also enhances ligand–target interactions, further increasing biological activity.

**Scheme 4:**
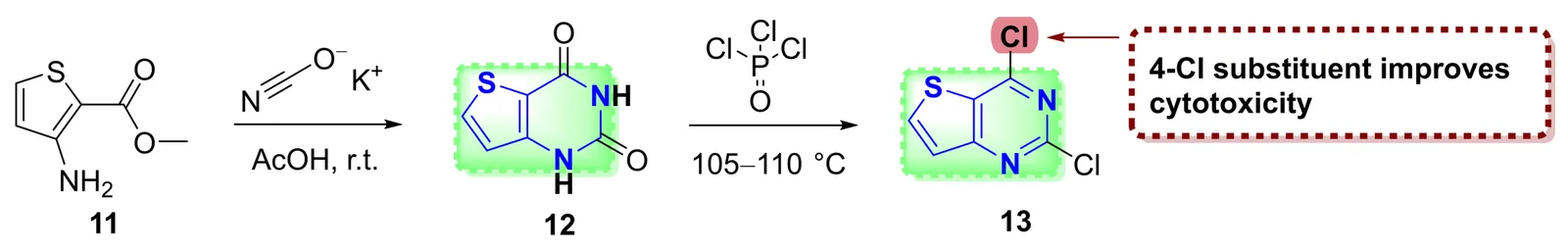
Structure and synthetic route of lead compound **13**.

Temburnikar et al. [110] developed a lead halogenated thieno[3,2-*d*]pyrimidine compound, **13**, that exhibits potent and selective antiproliferative activity. The synthesis of this compound began with the cyclization of methyl 3-aminothiophene-2-carboxylate **11** and potassium cyanate, producing intermediate **12**. Then, chlorination was performed using POCl_3_ (Scheme 4). Derivative **13** exhibited antitumor activity against several human cancer cell lines, including L1210, CEM, and HeLa, with respective IC_50_ values of 0.67, 5.2, and 3.9 μM. SAR studies revealed that the 4-chloro substituent is essential for activity and that the sulfur group enhances potency at a concentration of 1 μM. Lead compound **13** induced apoptosis in 60% of cells, primarily in the early stages. This lead compound provides a promising scaffold for further SAR development.

**Scheme 5:**
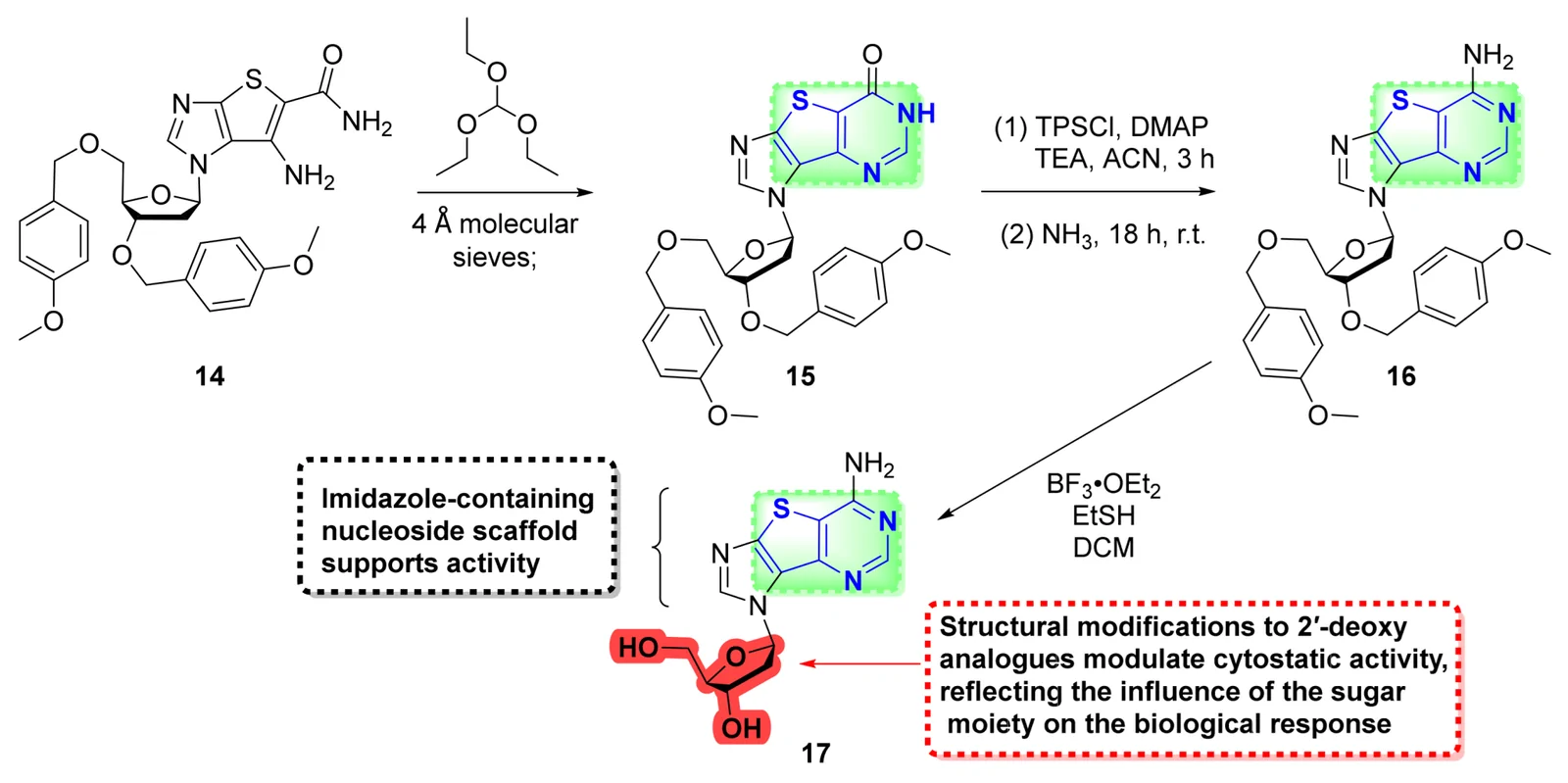
Structure and synthetic route of lead compound **17**.

A novel series of tricyclic pyrazolo-thieno[3,2-*d*]pyrimidine analogs bearing deoxyribose moieties was synthesized by Wauchope et al. [111] to assess their anticancer potential. The tricyclic scaffold **15** was constructed through a multistep synthesis involving triethoxymethane, followed by C-4 amination and deprotection of the methoxybenzyl group, yielding compound **17** (Scheme 5). The obtained compounds were screened for antiproliferative activity against L1210, CEM, HeLa, and HEL cell lines. Compound **17** exhibited moderate cytotoxicity, particularly against HEL cells (IC_50_ = 12 µM), while showing weaker effects on other tested lines. SAR analysis of **17** indicates that the tricyclic nucleoside scaffold containing an imidazole ring is favorable for maintaining cytotoxic activity. Additionally, structural modification of the 2′-deoxy analog alters cytostatic activity, underscoring the sugar moiety’s significant role in modulating biological responses. These results suggest that structural optimization, including the introduction of pharmacophoric groups, may enhance anticancer potency in future studies.

**Scheme 6:**
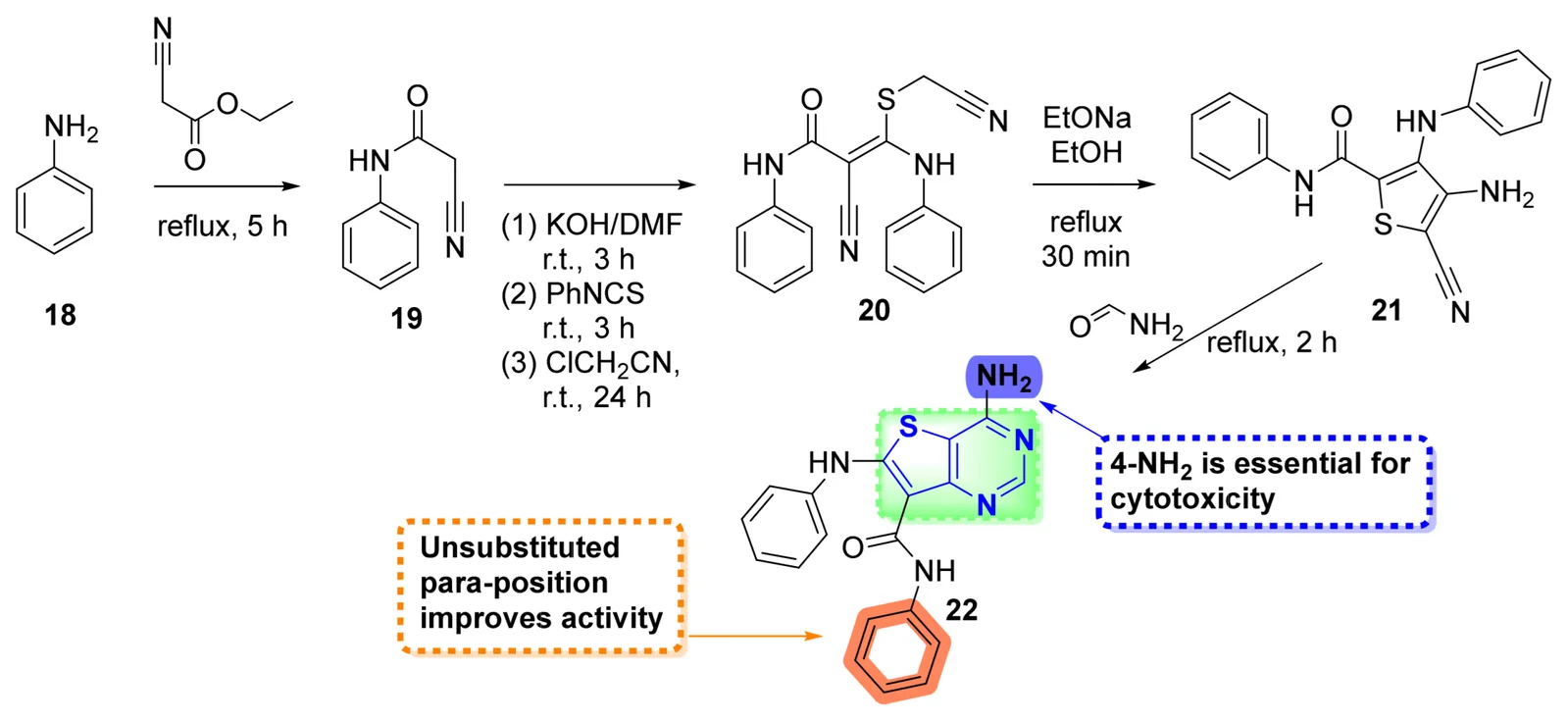
Structure and synthetic route of lead compound **22**.

Kandeel et al. [112] synthesized thieno[3,2-*d*]pyrimidine derivative **22** via sequential thiophene and thienopyrimidine cyclizations starting from ethyl 2-cyanoacetate (Scheme 6). Compound **22** exhibited potent cytotoxicity against MCF-7 cells with an IC_50_ of 2.04 nM. SAR analysis of derivative **22** shows that, compared to methyl substitution, an unsubstituted para-position on the phenylamide moiety favors cytotoxic activity. The presence of a 4-amino group at C-4 is crucial; replacement reduces potency, but improves cytotoxicity, compared to chloro, oxygen and aromatic carboxylic acids. Further studies are needed to clarify the mechanism of action.

**Scheme 7:**
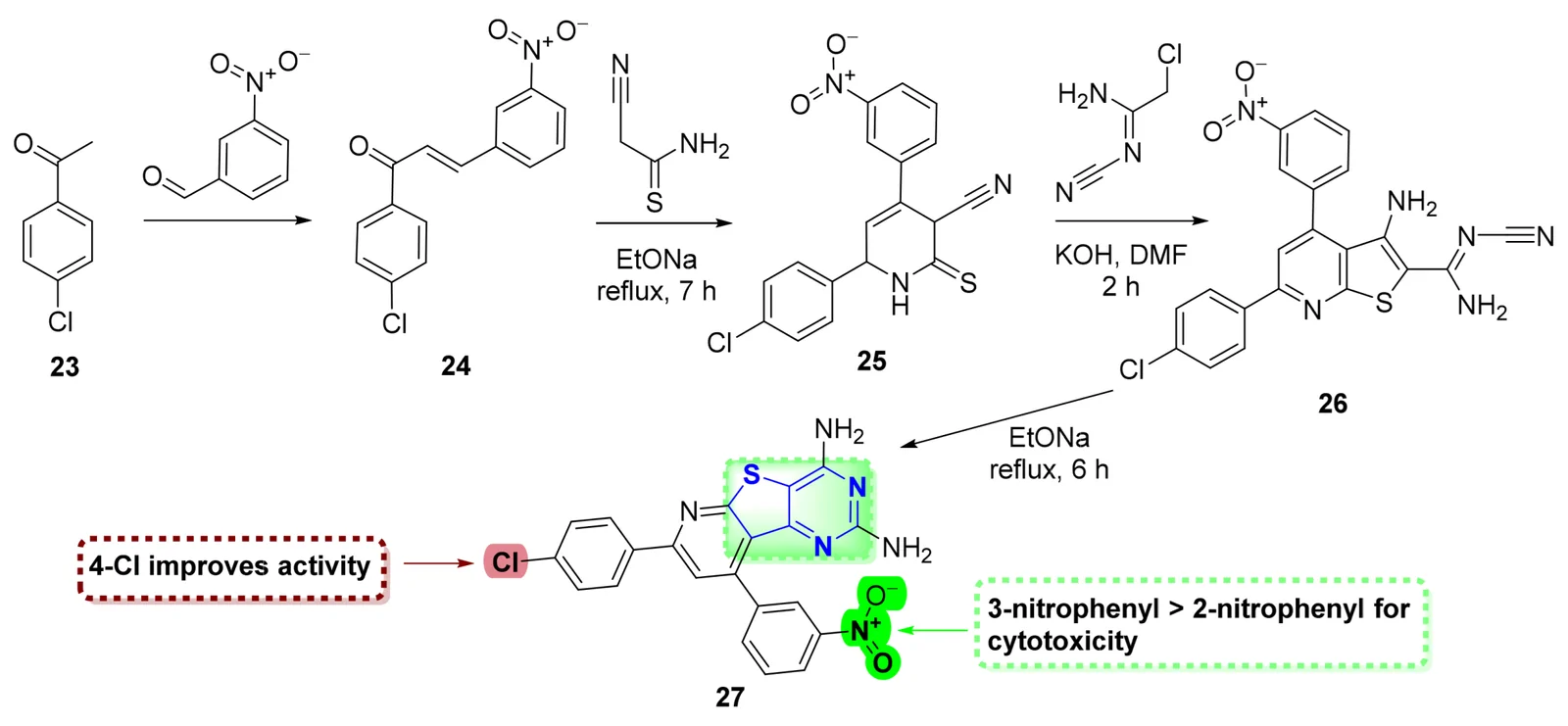
Structure and synthetic route of lead compound **27**.

Tricyclic thienopyrimidines and dipyridines were synthesized via the Thorpe–Ziegler reaction by Elansary et al. [113]. The target compound **27** was obtained through sequential cyclizations involving intermediate **25** and but-3-ynethioamide (Scheme 7). Pyridothienopyrimidine **27** exhibited the highest antiproliferative activity against MCF-7 cells (IC_50_ = 11.25 μM), comparable to doxorubicin (IC_50_ = 8.48 μM). SAR analysis of **27** indicates that the presence of a 4-chloro substituent enhances the antiproliferative activity of the pyridothienopyrimidine scaffold. Additionally, substitution with a 3-nitrophenyl group results in greater cytotoxicity than the 2-nitrophenyl analog, demonstrating the substantial impact of nitro group position. These results highlight **27** as a potential lead compound for anticancer development.

**Scheme 8:**
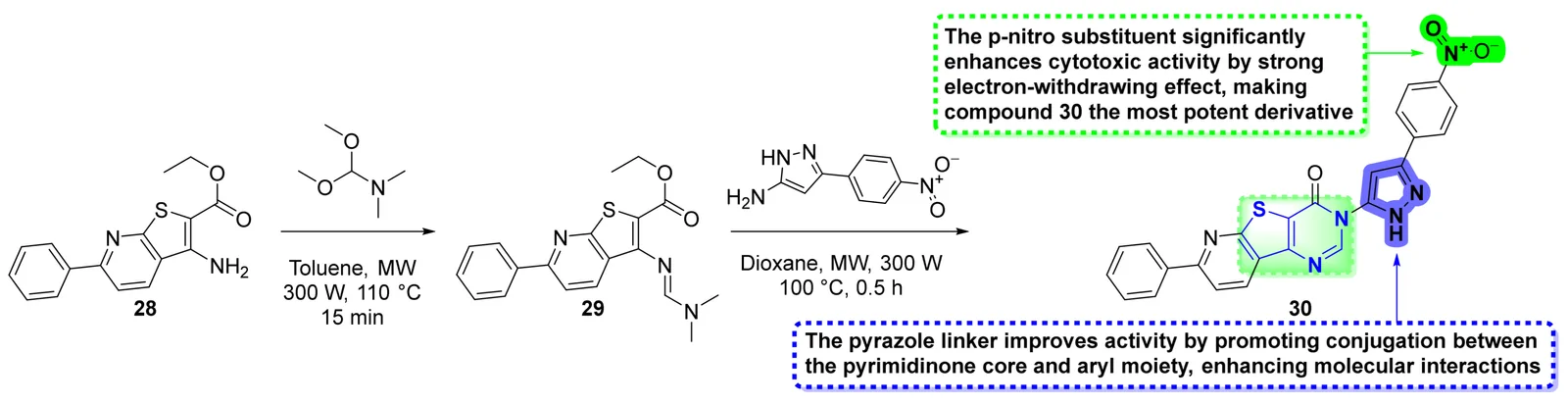
Structure and synthetic route of lead compound **30**.

Sanad and Mekky [114] synthesized a series of pyridine-condensed thieno[3,2-*d*]pyrimidinones using a three-component tandem protocol. The synthesis of lead compound **30** was achieved through microwave-assisted enamine formation, followed by intramolecular cyclization, yielding the pyrimidine ring (Scheme 8). Among the tested derivatives, compound **30** bearing a para-nitroaryl group demonstrated the most potent antiproliferative activity. It inhibited MCF-7, HepG2, and Caco-2 cancer cell lines with IC_50_ values of 4.20 μM, 3.65 μM, and 7.62 μM, respectively—outperforming 5-FU by 1.9–1.7 fold. Detailed analysis indicated that three fragments—pyrazole, nitrophenyl, and pyrimidine—played a key role in the biological response of **30**. The addition of a para-nitro group to **30** enhances its cytotoxicity, making it more potent than other analogs. The incorporation of a pyrazole linker between the pyrimidinone core and the aryl group further enhances biological activity, underscoring the importance of strong structural linkages. Computational models confirmed its binding to target proteins, supporting further study.

**Scheme 9:**
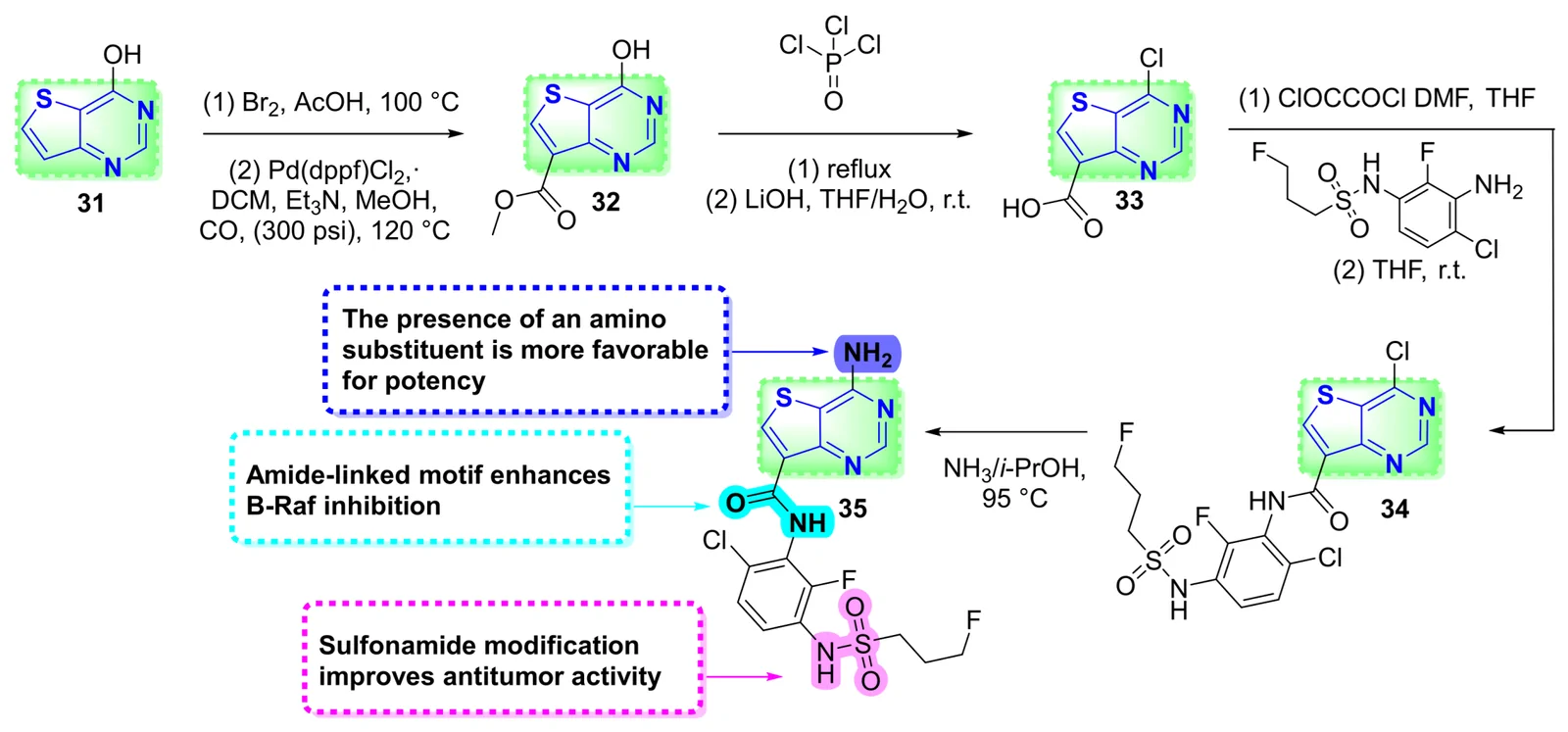
Structure and synthetic route of lead compound **35**.

A series of C-7-substituted thieno[3,2-*d*]pyrimidine derivatives was synthesized by Mathieu et al. [115]. Synthesis started from thieno[3,2-*d*]pyrimidin-4-ol **31**, which was halogenated at C-7, followed by Suzuki coupling and subsequent C-4 chlorination and amination to yield the target compound **35** (G945) as illustrated in Scheme 9. This lead compound showed potent antiproliferative activity against B-Raf^V600E^-mutant cell lines, including A375-X1, Colo205, and HT29, with EC_50_ values of 2, 5, and 24 nM, respectively. Notably, compound **35** was 11- to 152-fold more potent than inhibitors of wild-type B-Raf. In vivo studies using the Colo205 xenograft model demonstrated that the thienopyrimidine hybrid **35** inhibits tumor growth more effectively than the reference drug. SAR analysis of derivative **35** reveals that modification of the 4-aminothieno[3,2-*d*]pyrimidine scaffold to afford a sulfonamide-containing derivative leads to improved antitumor activity, which may be associated with enhanced in vivo efficacy and bioavailability. Furthermore, incorporation of an amide-linked aminopyrimidine moiety enhances B-Raf inhibitory activity and improves antiproliferative effects, confirming its importance for kinase-targeted activity.

**Scheme 10:**
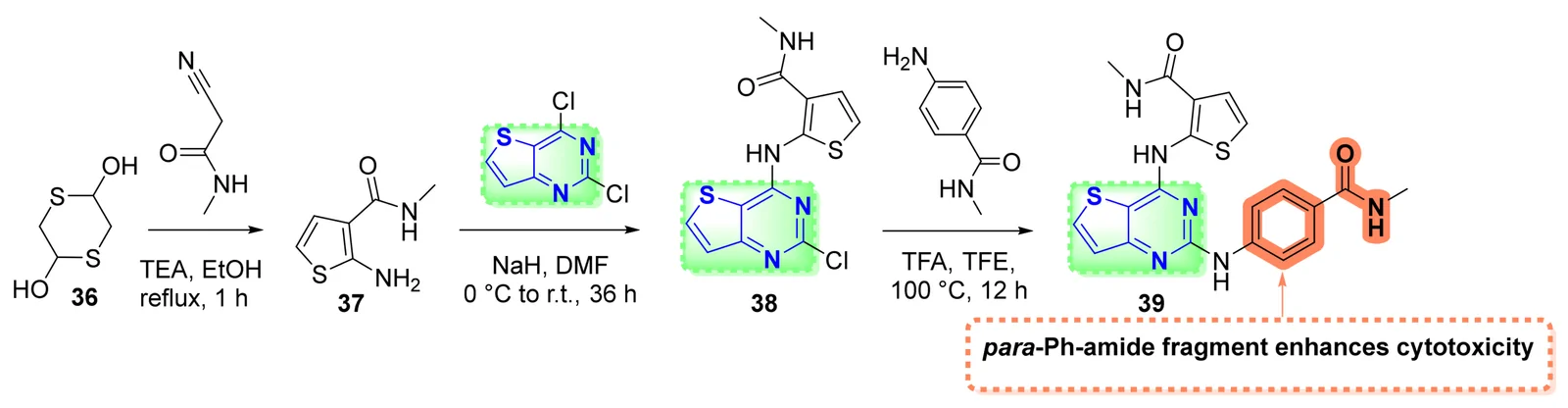
Structure and synthetic route of lead compound **39**.

Ding et al. [116] synthesized a new series of pyrimidinethiophene derivatives and assessed their cytotoxic potential against glioblastoma cells. Lead compound **39** was obtained via ring-transformation to generate an aminothiophene, which was then linked at C-4 and aminated at C-2 of the core scaffold (Scheme 10). In tests on U87-MG cells, **39** showed vigorous activity (IC_50_ = 3.38 μM), close to the standard TAE-226 (IC_50_ = 2.53 μM). SAR analysis of compound **39** demonstrates that para-substitution on the aromatic ring is favorable for antiproliferative activity, resulting in enhanced potency. Herein, this derivative potency was linked to key hydrogen bonds between the carbonyl and amine groups of the para-aryl unit. Docking analysis confirmed stable target interaction, and ADMET predictions suggest a basis for further optimization.

**Scheme 11:**
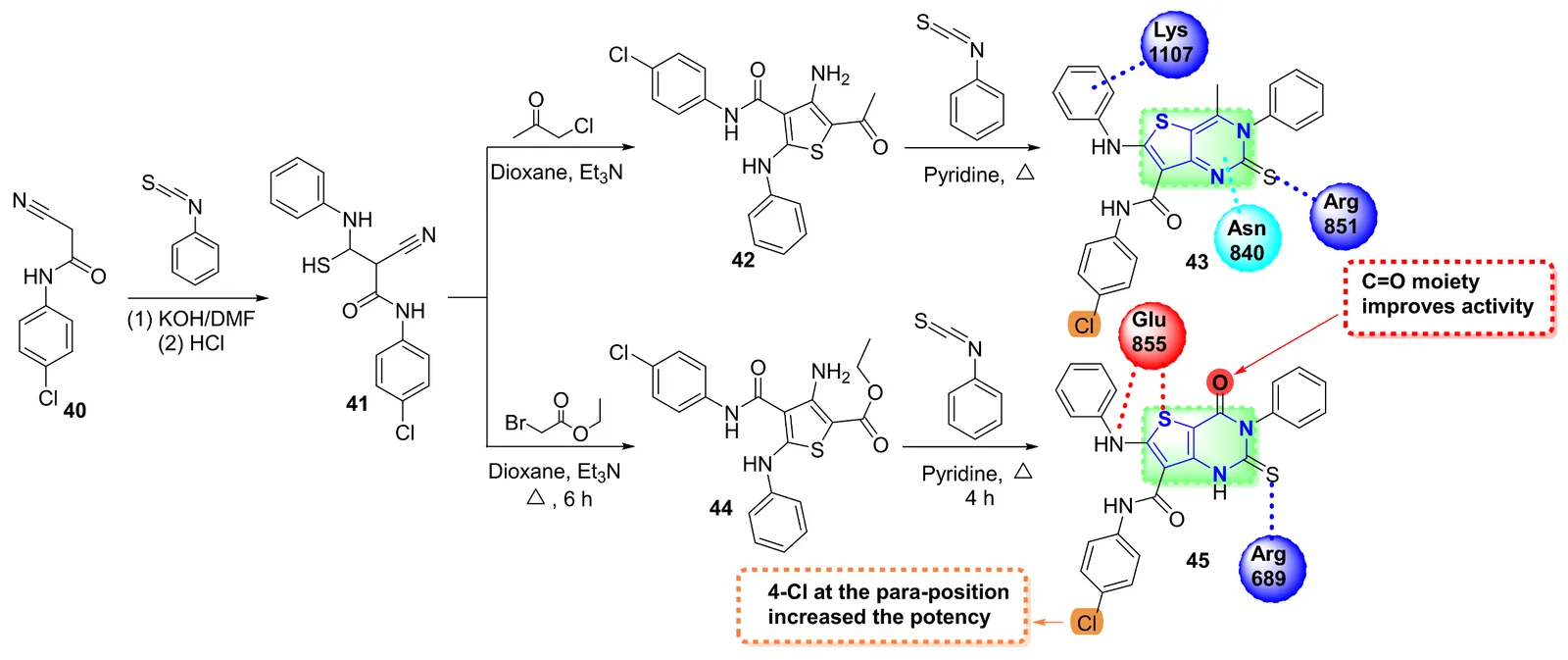
Structure and synthetic routes of lead compounds **43** and **45**.

Hossan and colleagues [117] synthesized novel thienopyrimidine derivatives from *N*-(4-chlorophenyl)-2-cyanoacetamide **40**, which was reacted with isothiocyanatobenzene and subsequently underwent thiophene and pyrimidine ring closures to obtain compounds **43** and **45** (Scheme 11). These were tested for antiproliferative activity against HepG2, HCT-116, and MCF-7 cell lines using the MTT assay, with 5-fluorouracil as a reference. Compounds **43** and **45** exhibited notable cytotoxicity, particularly against MCF-7 (IC_50_ = 14.53 and 11.17 μM), HepG2 (IC_50_ = 12.27 and 9.33 μM), and HCT-116 (IC_50_ = 15.75 and 10.63 μM). The SAR analysis of derivative **45** shows that having a carbonyl (C=O) group in the pyrimidinone skeleton improves antiproliferative activity, making it more potent than the methyl-substituted analog **43**. Molecular docking results support this, indicating that derivative **45** interacts more strongly with important amino acid residues in the active site. These results suggest further investigation of compounds **43** and **45** as potential anticancer agents.

**Scheme 12:**
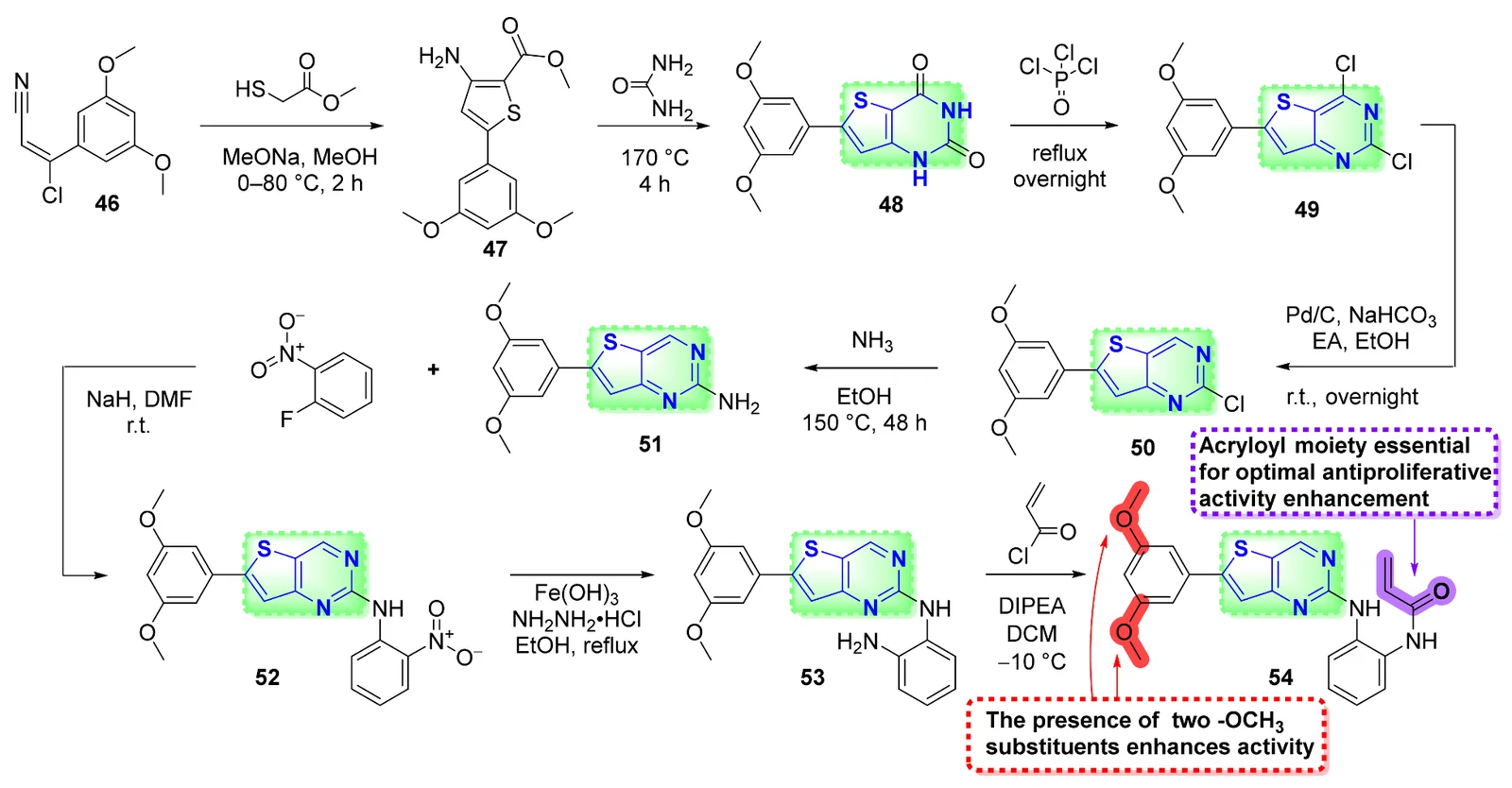
Structure and synthetic route of lead compound **54**.

Zhang et al. [118] developed a range of aryl-substituted thienopyrimidines and evaluated their antiproliferative properties. The lead compound **54** was synthesized via a cyclization process beginning with intermediate **46**, followed by sequential steps of halogenation, amination, nitroaryl coupling, reduction, and final acylation with acryloyl chloride. (Scheme 12). Hybrid compound **54** showed significant cytotoxic potency against the NCI-H1975, Ramos, A431, SNU-16, and NCI-H1581 cell lines, with IC_50_ values ranging from 0.6 to 2.6 µM, indicating its potential as a promising lead for anticancer drug development. SAR analysis of derivative **54** indicates that the presence of two methoxy groups enhances its antiproliferative activity and increases its cytotoxic potency. Introduction of an acryloyl group also boosts biological activity, highlighting its role in maintaining strong antiproliferative effects.

**Scheme 13:**
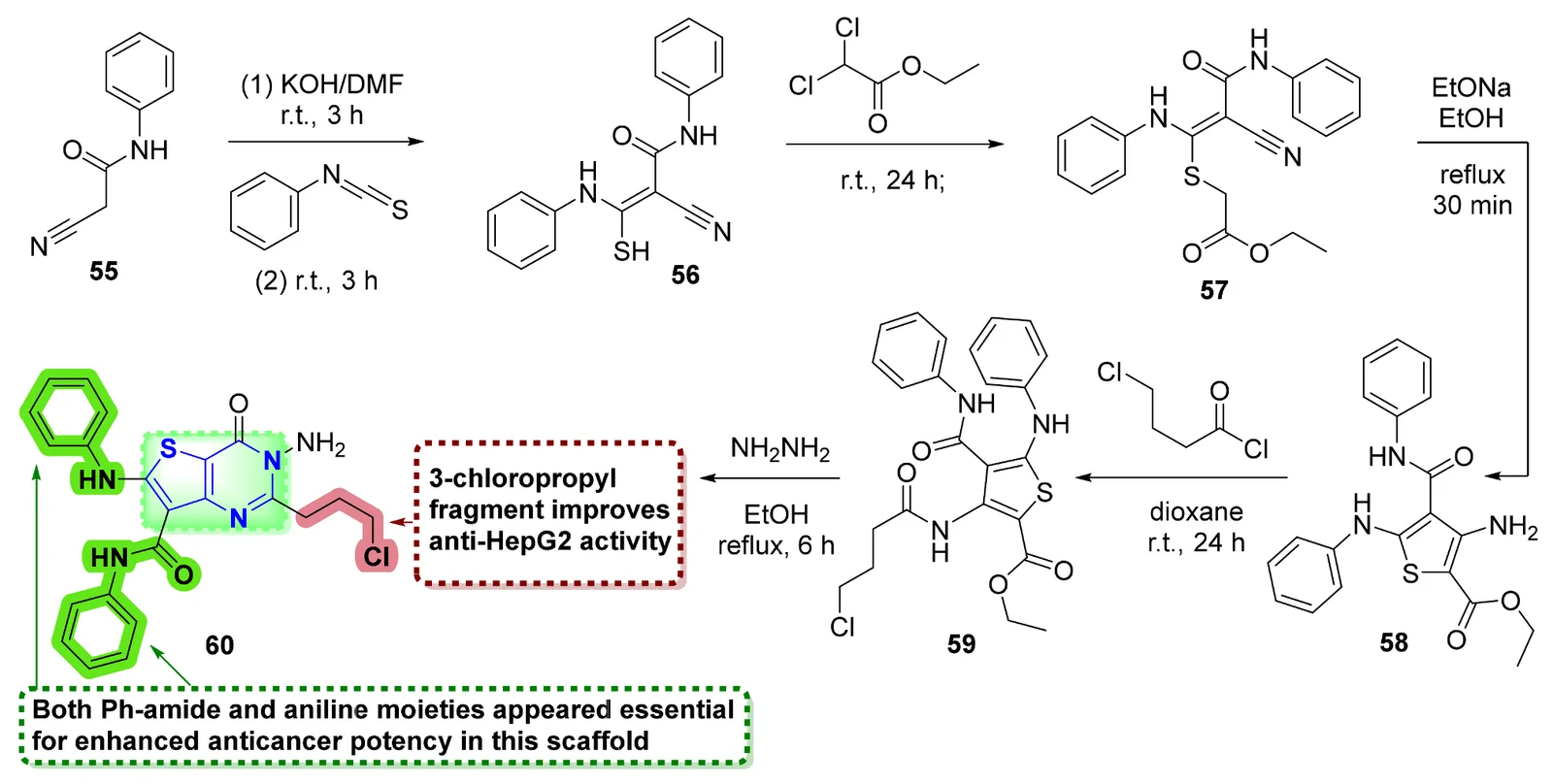
Structure and synthetic route of lead compound **60**.

Labib and Lamie [119] synthesized a series of thienopyrimidine–aniline derivatives via a multistep route starting from 2-cyano-*N*-phenylacetamide. Cyclization with isothiocyanatobenzene and ethyl dichloroacetate gave thiophene intermediate **58**, which was acylated with 4-chlorobutanoyl chloride and then cyclized with hydrazine to afford the target compound **60** (Scheme 13). Among the tested compounds, **60** displayed the highest cytotoxic activity against HepG2 liver cancer cells, with an IC_50_ of 0.0196 µM—nearly twice as potent as 5-fluorouracil (IC_50_ = 0.0384 µM). SAR analysis of **60** revealed that the presence of both phenylamide and aniline moieties appeared essential for enhanced anticancer potency in this scaffold. In addition, incorporating a 3-chloropropyl fragment improves cytotoxic activity against HepG2 cells.

**Scheme 14:**
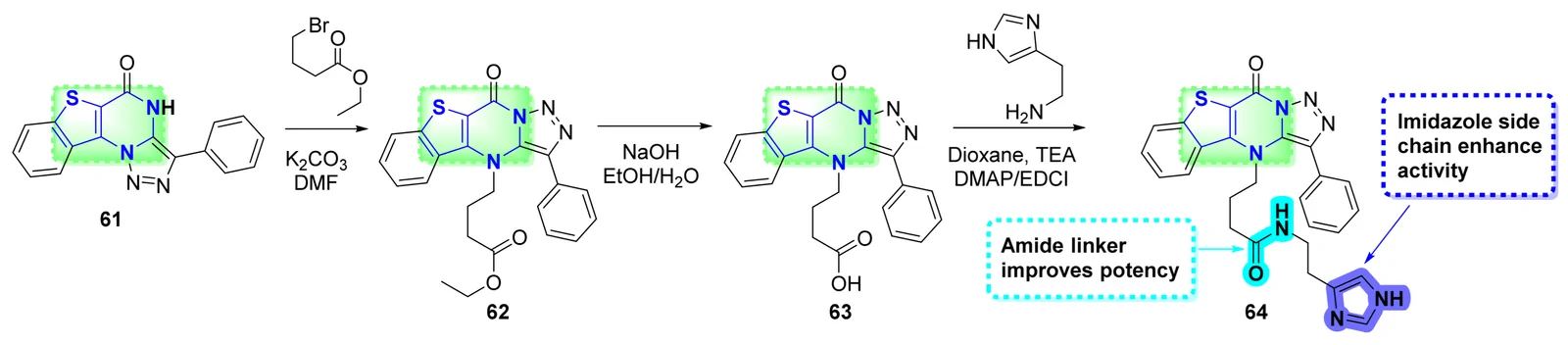
Structure and synthetic route of lead compound **64**.

Lauria et al. [120] synthesized a novel linear thieno[3,2-*d*][1–3]triazolo [1,5-*a*]pyrimidine **64** via a Dimroth rearrangement of a tetracyclic thieno[3,2-*d*]pyrimidine **61**. The route involved the alkylation of intermediate **61** with ethyl 4-bromobutanoate, followed by hydrolysis and formation of an amide bond with 2-(1*H*-imidazol-4-yl)ethan-1-amine (Scheme 14). Compound **64** was evaluated for antiproliferative activity using the NCI-60 human cancer cell line panel. It demonstrated broad cytotoxic effects with a mean IC_50_ of ~525 nM, showing stronger inhibition against colon (IC_50_ = 138 nM) and leukemia (IC_50_ = 49 nM) subpanels. Remarkable potency was observed in HCT-116, SF-539, CCRF-CEM, and SN12C cells, with IC_50_ values between 11 and 20 nM. Compound **64**, with strong activity against OVCAR-8 cells (IC_50_ < 10 nM) and low toxicity (LC_50_ > 100 µM), showed no adverse effects in mice over two weeks and is now under in vivo investigation to clarify its mechanism and optimize its unique ring system. SAR analysis of derivative **64** shows that the extended fused heterocyclic scaffold enhances antiproliferative activity and improves potency. Furthermore, the incorporation of an imidazole-containing side chain and an amide linker enhances biological activity, underscoring their importance in maintaining effective cytotoxicity.

**Scheme 15:**
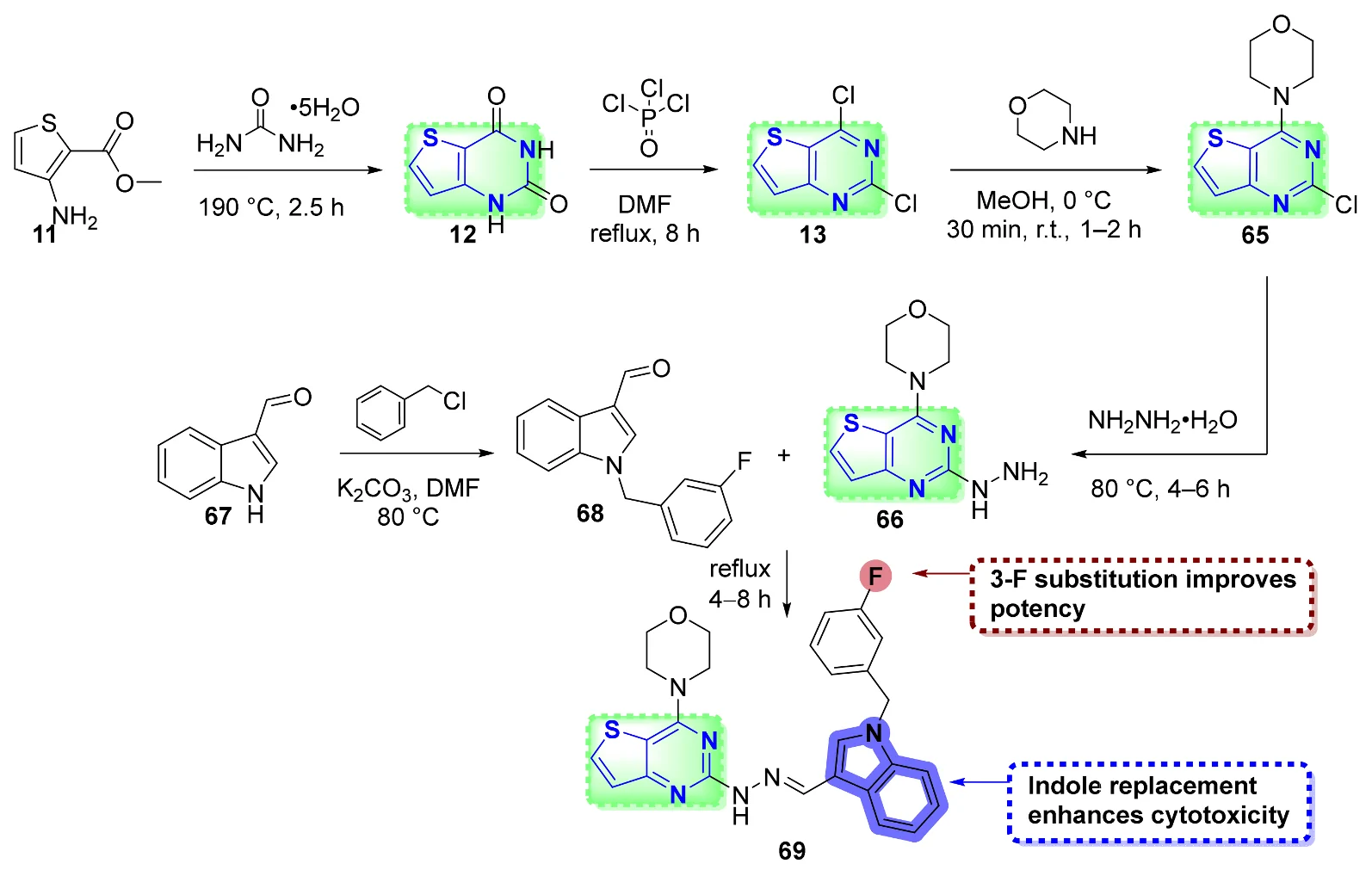
Structure and synthetic route of lead compound **69**.

Zhu et al. [121] synthesized a series of thieno[3,2-*d*]pyrimidines featuring C-2-hydrazinyl and C-4-morpholine groups, with **69** identified as the most potent compound. The synthesis began with methyl 3-aminothiophene-2-carboxylate, which was cyclized and subsequently chlorinated to yield compound **13**. This compound was then subjected to C-2 and C-4 substitutions, yielding **66**. Condensation with the 3-fluorobenzyl-substituted indole-3-carbaldehyde **68** afforded lead compound **69** (Scheme 15). Compound **69** exhibited strong antiproliferative activity against H460 (0.23 µM), HT-29 (0.39 µM), H226 (0.91 µM), SGC-7901 (0.8 µM), and MDA-MB-231 (1.11 µM) cell lines, significantly outperforming GDC0941. It also showed comparable potency against A549, HepG2, U87MG, and H1975 cells (IC_50_ 1.0–19 µM). The 3-F substitution at the benzyl ring was crucial for its enhanced cytotoxicity, resulting in up to a 66-fold increase in activity compared to the reference drug. In addition, replacing the benzimidazole moiety with an indole fragment increases cytotoxic activity, demonstrating the advantages of this structural modification.

**Scheme 16:**
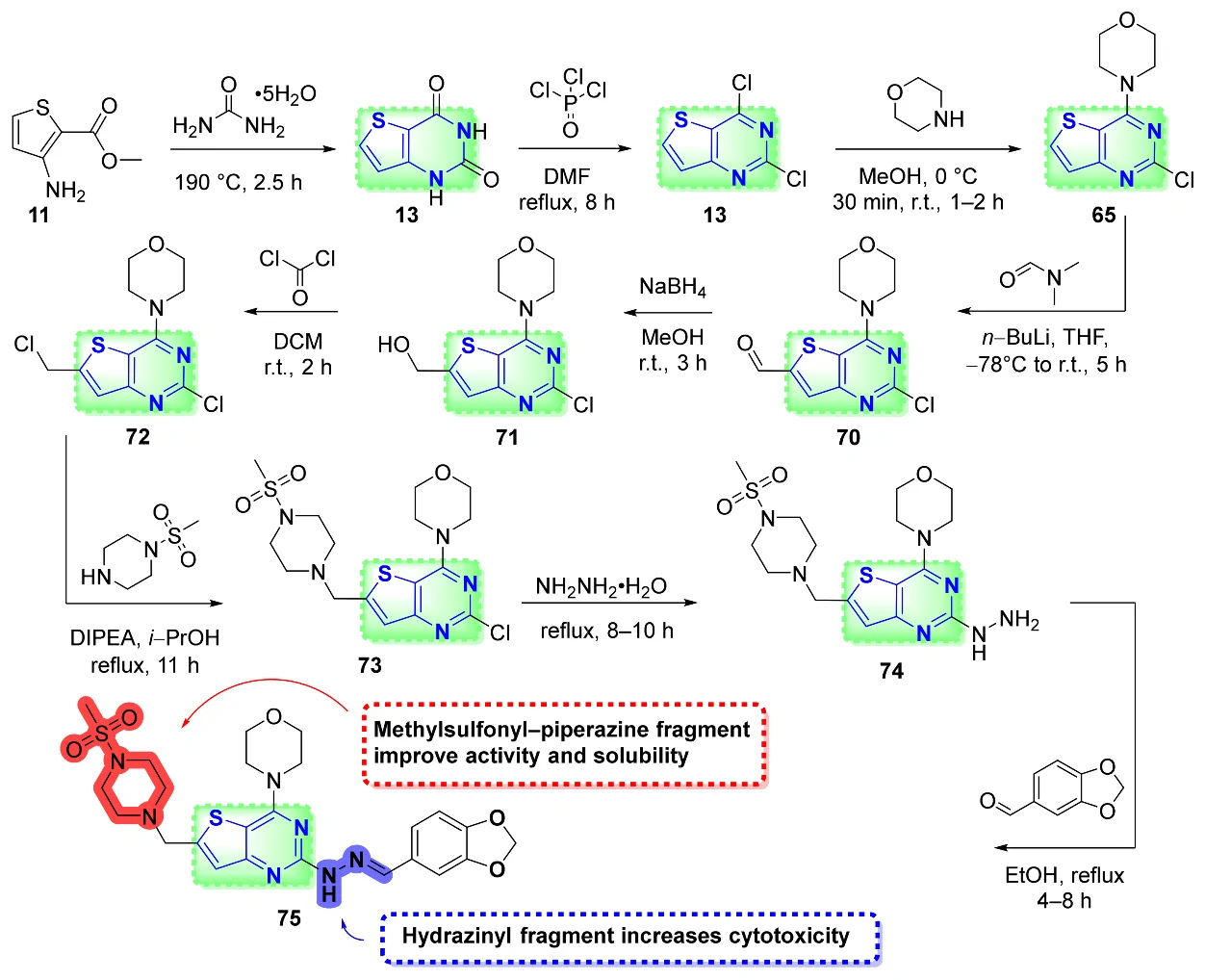
Structure and synthetic route of lead compound **75**.

Zhu et al. [122] synthesized morpholino-thieno[3,2-*d*]pyrimidine derivatives via cyclization of methyl 3-aminothiophene-2-carboxylate with urea, followed by chlorination, morpholine substitution, and stepwise modification at C-6 and C-2 to yield compound **75**. Key steps included aldehyde formation, reduction, phosgene chlorination, and coupling with benzo[*d*][1,3]dioxole-5-carbaldehyde (Scheme 16). Compound **75** exhibited potent cytotoxicity against H460 (IC_50_ = 0.003 µM), HT-29 (IC_50_ = 0.42 µM), and MDA-MB-231 (IC_50_ = 0.74 µM), demonstrating superior activity compared to GDC-0941. It also inhibited A549, H226, U87MG, HepG2, and SGC-7901 cells with good selectivity over normal WI-38 (SI = 2467 for H460). The SAR supported its potential as a selective anticancer agent. Derivative **75**, bearing a methylene hydrazinyl fragment, demonstrated increased cytotoxic activity compared with the indole and imidazole analogs. The introduction of a methylsulfonyl/piperazine fragment enhances solubility.

**Scheme 17:**
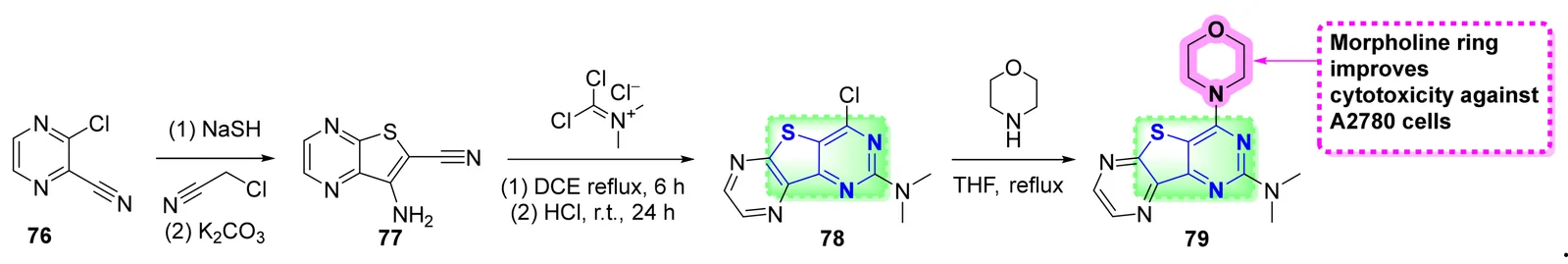
Structure and synthetic route of lead compound **79**.

Fernández-Mato et al. [123] synthesized a series of substituted pyrazino-thieno[3,2-*d*]pyrimidines and triazines via stepwise cyclizations starting from 2-chloro-3-ethynylpyrazine, yielding tricyclic core **78** and its morpholine-substituted analog **79** (Scheme 17). Derivatives were tested for antiproliferative activity against LLC-PK1, NCI-H460, HeLa 229, and A2780 cell lines. Lead compound **79** showed moderate anticancer activity, particularly against A2780 cells (IC_50_ = 8.6 µM), although it was less potent than cisplatin. The obtained results suggest that structural modifications, particularly morpholine substitution, can enhance the cytotoxic profiles of pyrazine-fused thienopyrimidines.

**Scheme 18:**
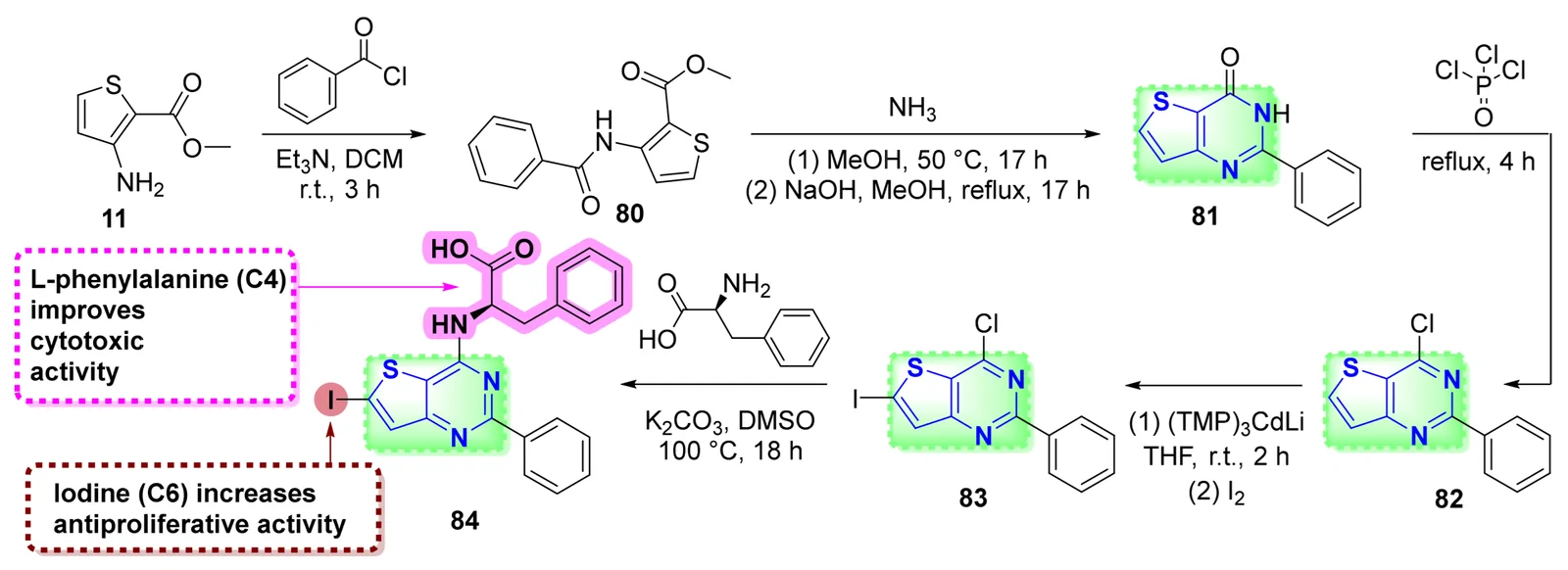
Structure and synthetic route of lead compound **84**.

A series of thienopyrimidines, including lead compound **84**, was synthesized via a deprotonation–trapping strategy, as reported by Snegaroff et al. [124]. The target lead compound was synthesized starting from the intracyclization of methyl 3-aminothiophene-2-carboxylate with benzoyl chloride. Subsequent chlorination and iodination, followed by final condensation with L-phenylalanine, afforded compound **84** (Scheme 18). Derivative **84** was tested for antitumor activity against the three human cancer cell lines, HepG2, MCF7, and HeLa, and exhibited moderate antiproliferative activity, with IC_50_ values of 5.39 µM, 2.99 µM, and 9.98 µM, respectively. The presence of the amino acid fragment in the system and iodine substitution at position 6 may contribute to the observed bioactivity, suggesting the potential of future hybrid thienopyrimidine derivatives.

### 3.2. EGFR-Targeted Anticancer Activity

**Scheme 19:**
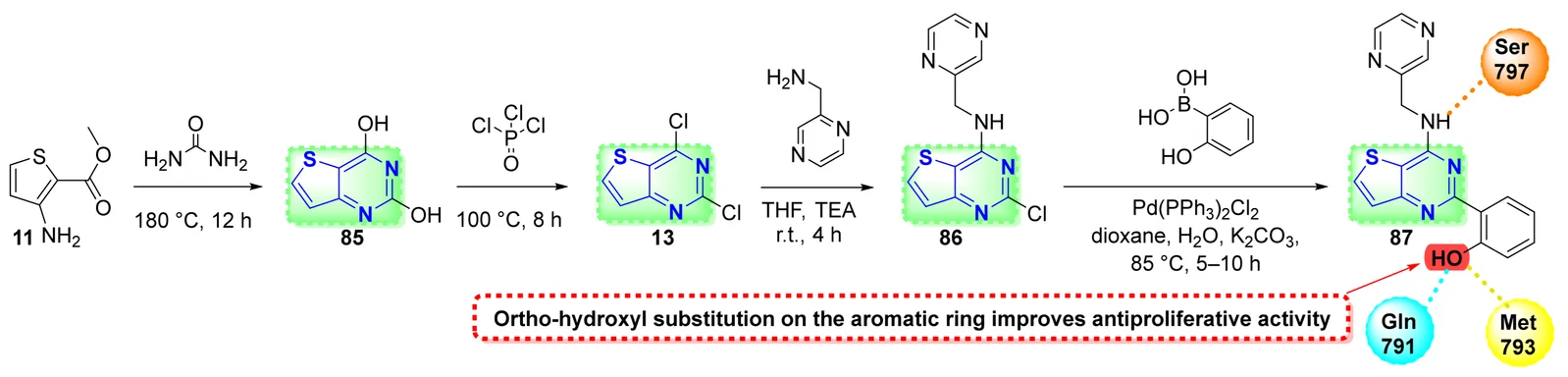
Structure and synthetic route of lead compound **87**.

Abnormal activation of the epidermal growth factor receptor (EGFR) is key to tumor growth, survival, and spread, so it is an important target in cancer drug research [125,126]. Many heterocyclic small molecules have been created to block EGFR and its mutant forms [127]. These molecules can inhibit the growth of EGFR-driven cancers and may help overcome resistance to current tyrosine kinase inhibitors [128,129]. Zhang et al. [130] synthesized a series of 2-aryl-4-aminothienopyrimidine derivatives by structural modification of the 2-aryl-4-aminoquinazoline scaffold, a classical backbone of fourth-generation EGFR inhibitors. Lead compound **87** was prepared via cyclization, halogenation, nucleophilic aromatic substitution (SNAr) with the corresponding amine, and subsequent Suzuki–Miyaura coupling (Scheme 19). In vitro assays showed that **87** exhibited more potent antiproliferative activity than AZD9291 against A549 (IC_50_ = 0.77 μM), HeLa (IC_50_ = 20.21 μM), and H1975 (IC_50_ = 6.90 μM) cells. In Ba/F3-EGFR^Del19/T790M/C797S^ cells, compound **87** inhibited proliferation by 98.9% at 10 μM. Also, derivative **87** inhibited mutant EGFR forms more strongly than the wild-type, particularly L858R/T790M and Del19/T790M/C797S. SAR analysis of **87** indicates that ortho-hydroxyl substitution on the aromatic ring improves antiproliferative activity. Docking analysis showed hydrophobic binding near MET790 and hydrogen bonding with GLN791, MET793, and SER797. In a xenograft model, **87** reduced tumor size by 55.9%, similar to AZD9291, and was well tolerated, with no signs of systemic toxicity.

**Scheme 20:**
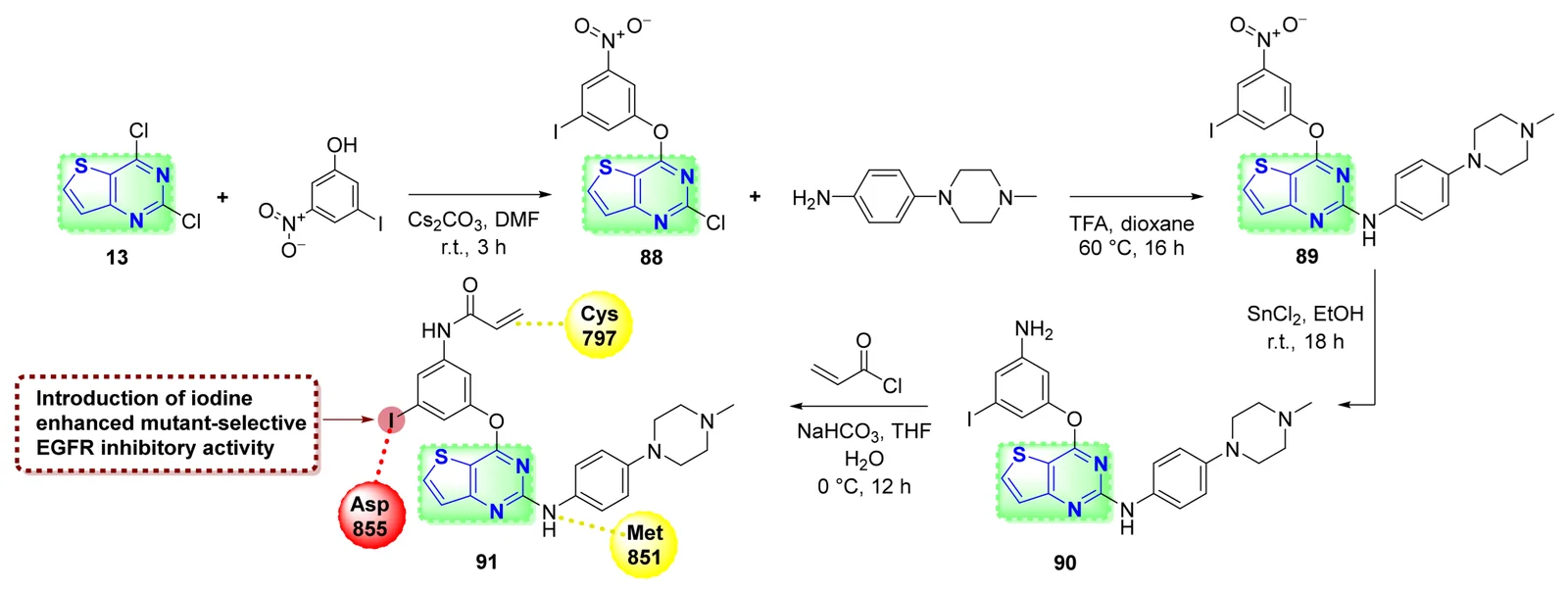
Structure and synthetic route of lead compound **91**.

Structural modification of Olmutinib, guided by docking studies against mutant EGFR, led to the design of compound **91**, as reported by Fawwaz et al. [131]. Its synthesis involved Williamson ester formation, SNAr, nitro reduction, and acryloylation (Scheme 20). Compound **91** showed similar binding energy and orientation to Olmutinib at the EGFR^T790M^ site. The addition of an iodine atom improved selectivity. In vitro tests revealed inhibitory activity against EGFR^L858R/T790M^ and EGFR^L858R^ mutants, with IC_50_ values of 10.49 and 14.55 μM, respectively, although these activities were lower than those of Olmutinib.

**Scheme 21:**
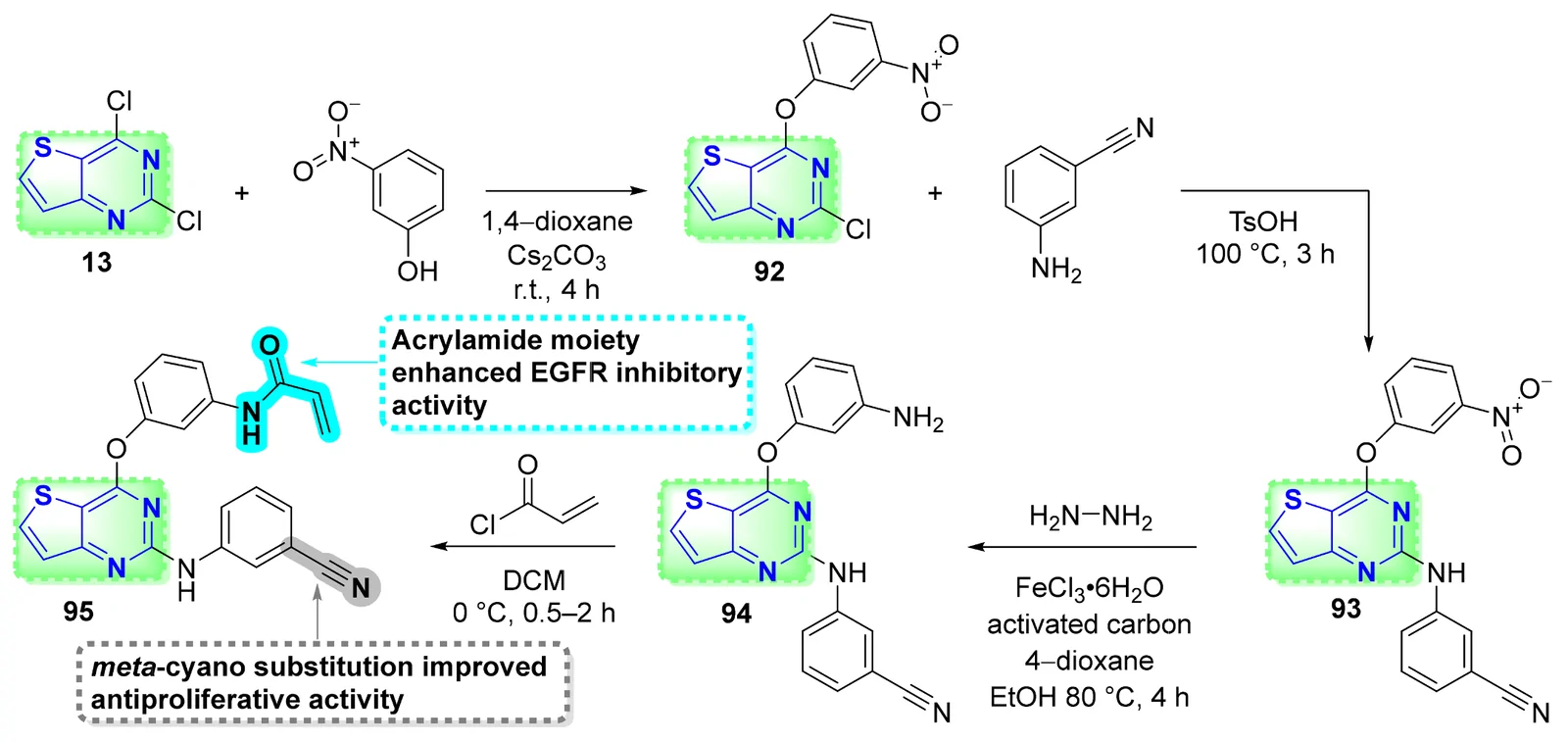
Structure and synthetic route of lead compound **95**.

Derivative **95** was prepared via a multistep route beginning with methyl-3-aminothiophene-2-carboxylate [132]. The sequence involved cyclization with urea, chlorination using DMF, nucleophilic substitution with *m*-nitrophenol, and final acylation with acryloyl chloride (Scheme 21). As the lead molecule, **95** demonstrated potent inhibition of EGFR^T790M/L858R^ mutations, with an IC_50_ of 699.2 nM in H1975 cells and potent antiproliferative activity against A549 (IC_50_ = 4.34 μM), A431 (IC_50_ = 3.79 μM), and HeLa (IC_50_ = 6.39 μM) cell lines. It achieved over 100% inhibition of EGFR^T790M/L858R^ at 1 µM and showed 15-fold selectivity over EGFR^WT^. The compound exhibited negligible activity against c-Met, KDR, and mTOR, highlighting its selectivity as a dual-mutation EGFR inhibitor. SAR analysis revealed that the acrylamide moiety was essential for potent EGFR inhibition, while the meta-cyano substituent enhanced antiproliferative activity and mutant selectivity.

**Scheme 22:**
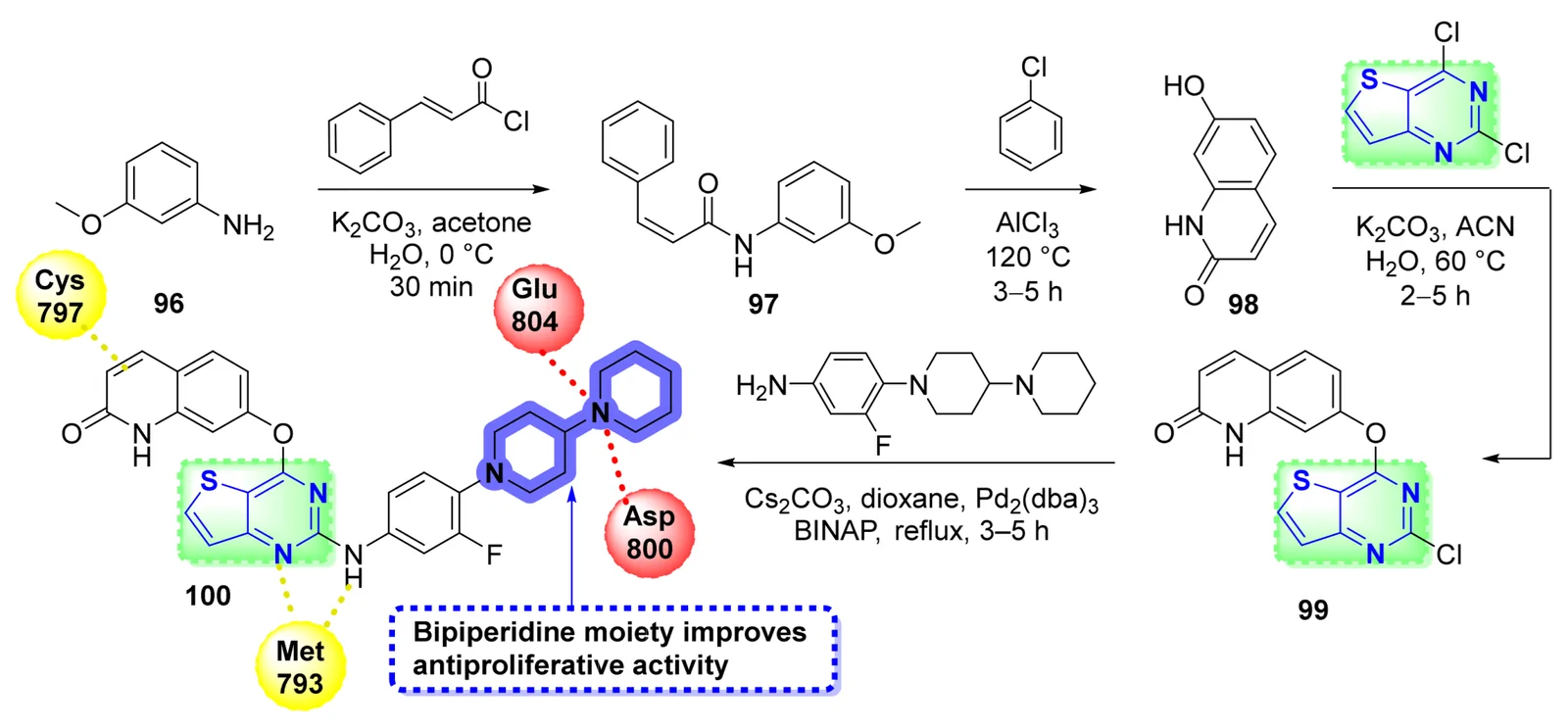
Structure and synthetic route of lead compound **100**.

Chen et al. [133] developed the lead compound **100** through a multistep synthesis involving acylation of 3-methoxyaniline with cinnamoyl chloride, followed by AlCl_3_-mediated cyclization to form a quinolinone scaffold. Subsequent SNAr with 2,4-dichlorothieno[3,2-*d*]pyrimidine was followed by Pd-catalyzed Buchwald–Hartwig coupling, which yielded compound **100** (Scheme 22). Kinase profiling revealed that compound **100** strongly inhibited the EGFR^L858R/T790M^ mutant, with an IC_50_ of 0.11 μM, while showing much weaker inhibition of wild-type EGFR (IC_50_ = 12.43 μM), yielding a selectivity index of 113. This value was notably higher than that of the reference compound Olmutinib (SI = 43). SAR analysis indicated that incorporation of the bipiperidine moiety significantly enhanced antiproliferative potency and mutant EGFR selectivity. Further support for its mutant selectivity came from docking simulations and analyses of EGFR-related signaling pathways, indicating that **100** is a promising candidate for targeted inhibition of EGFR resistance mutations.

**Scheme 23:**
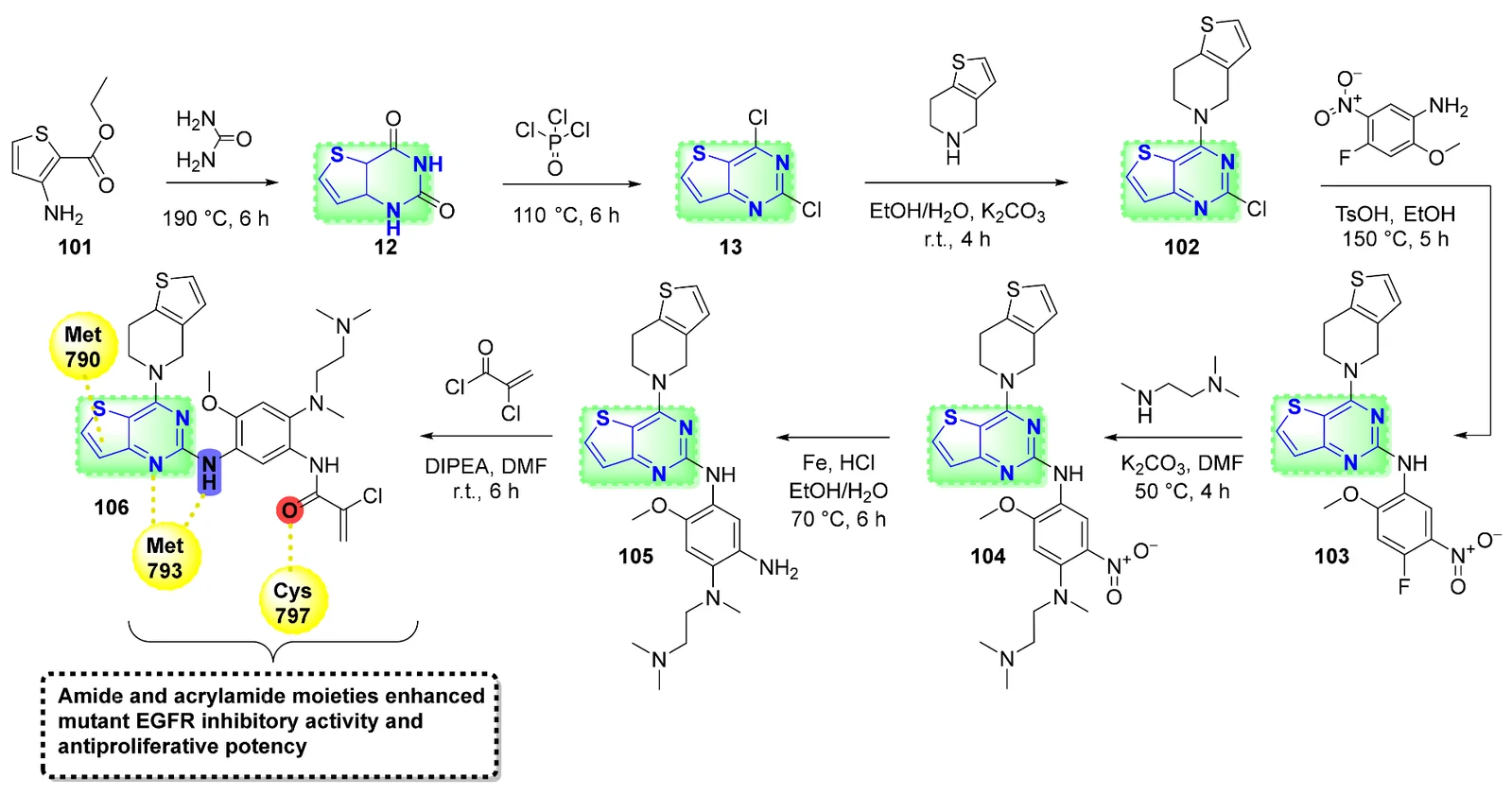
Structure and synthetic route of lead compound **106**.

Pyrido- and thienopyrimidine derivatives were synthesized as EGFR inhibitors, and compound **106** was identified as the lead compound, as reported by Fu et al. [134]. The synthesis involved a regioselective SNAr, followed by SNAr on the pyrimidine ring and subsequently on the fluorinated ring. Reduction of the nitro group followed by amidation afforded lead compound **106** (Scheme 23). **106** showed potent antiproliferative activity in H1975 (IC_50_ = 0.087 μM), A549 (IC_50_ = 1.508 μM), and H460 (IC_50_ = 2.335 μM) cells. At 0.1 μM, it inhibited EGFR^L858R/T790M^ by 90.3%. The compound exhibited 76.6-fold selectivity for the mutant EGFR (IC_50_ = 13 nM) over wild type (IC_50_ = 996 nM). This selectivity was attributed to the acryl chloride moiety introduced during synthesis.

**Scheme 24:**
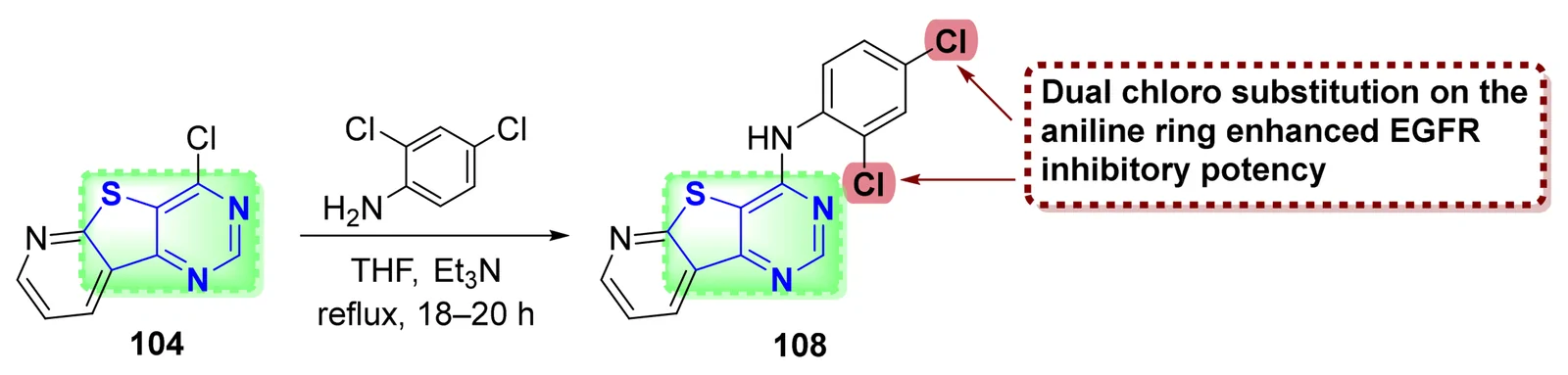
Structure and synthetic route of lead compound **108**.

A series of pyridothienopyrimidine derivatives bearing aniline or benzylamine moieties at the C-4 position was designed based on known EGFR inhibitors, as reported by Abdel Aziz et al. [135]. The tricyclic thienopyrimidine chloride was coupled with 2,4-dichloroaniline to afford compound **108** (Scheme 24). This compound showed potent EGFR inhibition (IC_50_ = 36.7 nM), outperforming erlotinib (IC_50_ = 486 nM). Compound **108** showed cytotoxic activity across several tumor cell lines, including KM12, HCC-2998, MALME-3M, SK-MEL-28, IGROV1, OVCAR-3, and MDA-MB-231, with GI_50_ values ranging from 10 to 98 nM. In the HS 578T breast cancer line, the effect was weaker (GI_50_ > 100 nM). In A549 non-small-cell lung cancer cells, **108** inhibited proliferation with an IC_50_ of 0.03 µM. SAR analysis revealed that dual ortho/para-chloro- substitution on the aniline ring significantly enhanced EGFR inhibitory potency and antiproliferative activity, likely due to favorable hydrophobic interactions within the EGFR binding pocket. These results suggest that **108** maintains consistent antiproliferative activity, particularly in EGFR-expressing models.

**Scheme 25:**
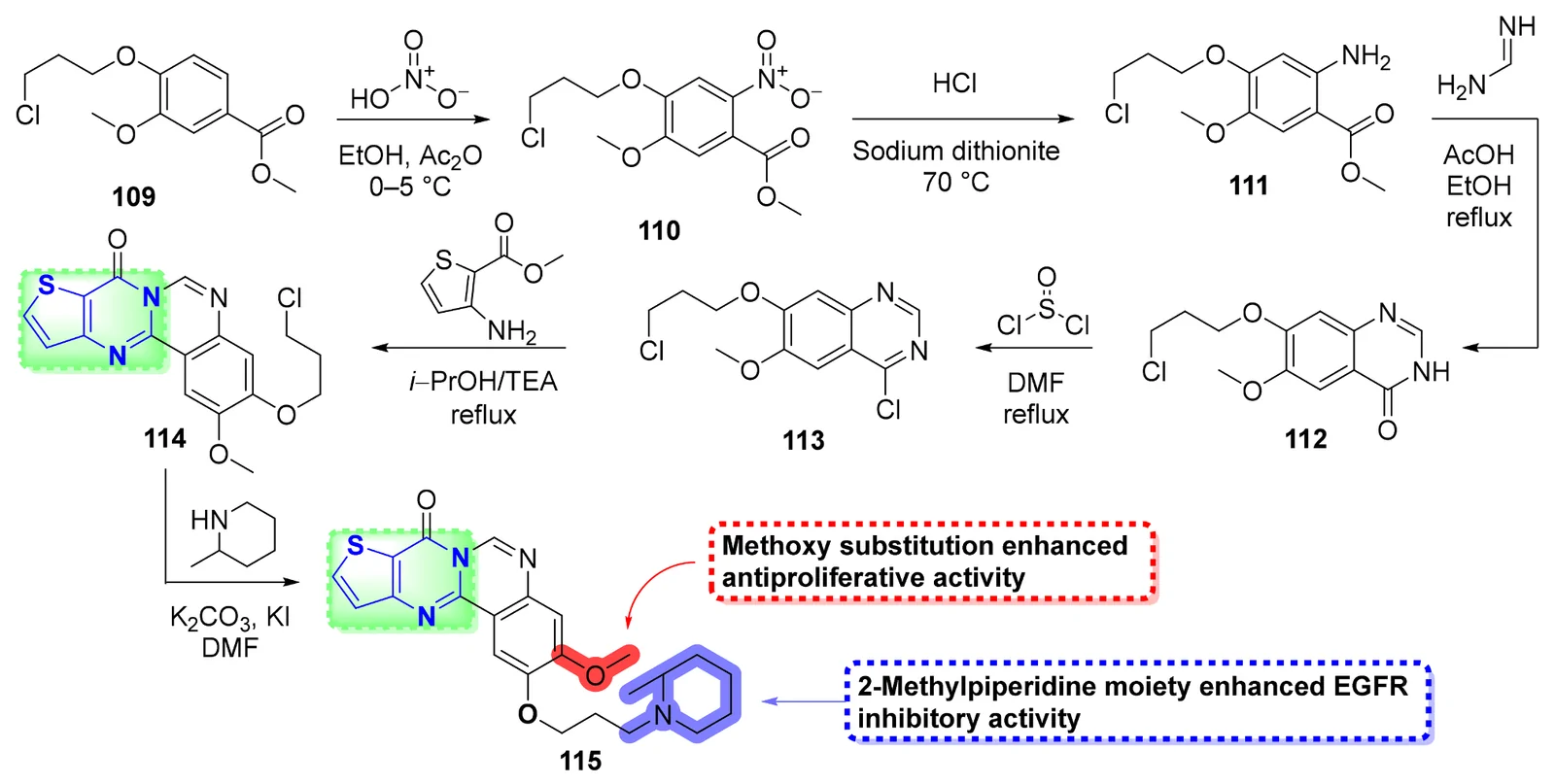
Structure and synthetic route of lead compound **115**.

Quinazoline-[3,4-*a*]thieno[3,2-*d*]pyrimidin-8-one derivatives featuring diverse amine moieties, including morpholine, *N*-methylpiperazine, and pyrrole, were synthesized, as reported by Zheng et al. [136]. The synthetic route involved nitration, reduction, and cyclization steps starting from methyl-4-(3-chloropropoxy)-3-methoxybenzoate, followed by the formation of a thiophene-based ring and final coupling with 2-methylpiperidine (Scheme 25). Compound **115** showed the highest cytotoxicity in vitro, particularly against MiaPaCa2 (IC_50_ = 9.04 µM) and DU145 (IC_50_ = 21.05 µM) cell lines. The improved activity was attributed to the 2-methylpiperidinyl moiety at C-2, while methoxy substitution enhanced binding affinity and antiproliferative potency. Notably, compound **115** also exhibited enhanced EGFR inhibition, highlighting its therapeutic potential.

**Scheme 26:**
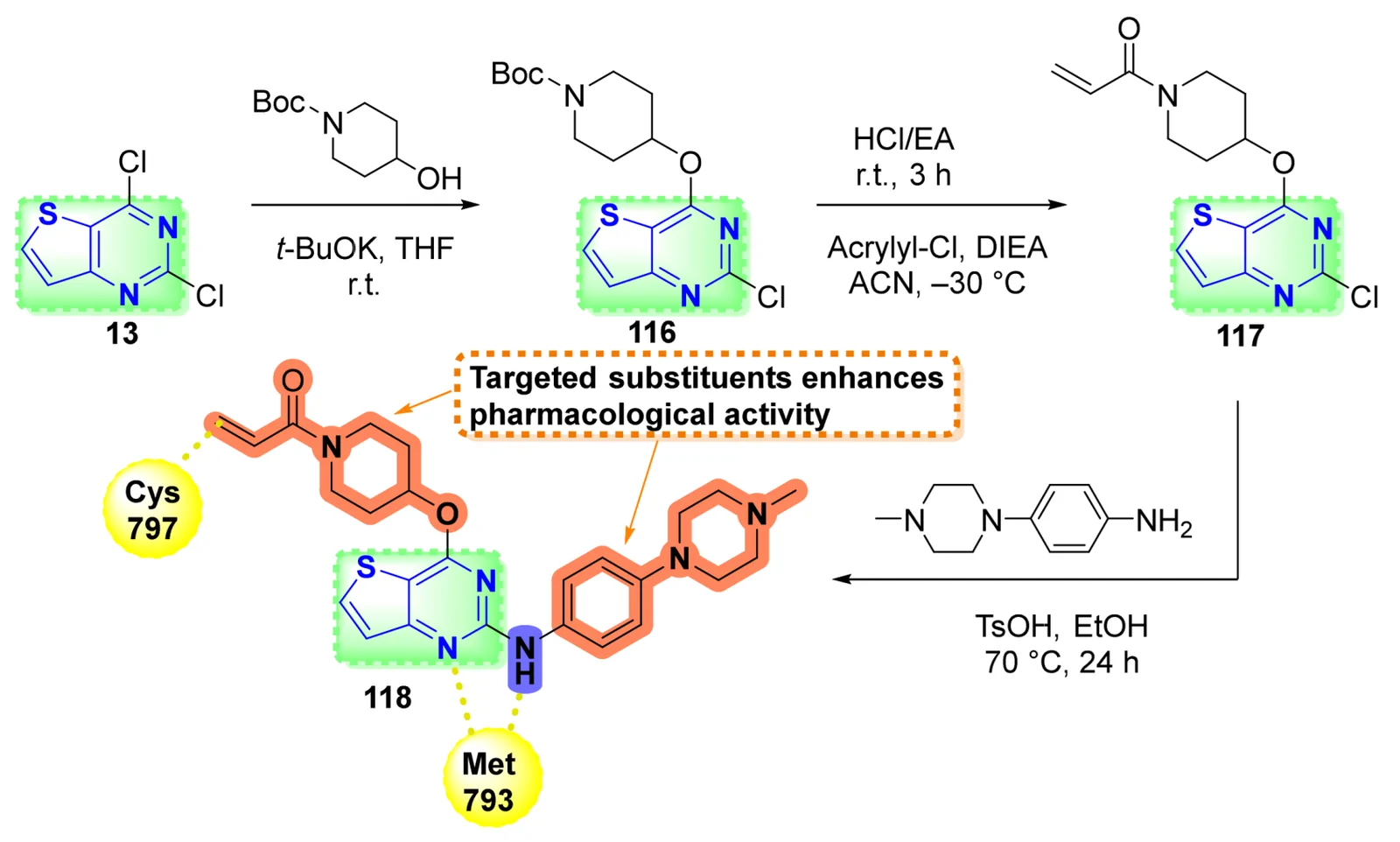
Structure and synthetic route of lead compound **118**.

2-Arylamino-4-(piperidin-4-yloxy)pyrimidines were synthesized by Tian and colleagues [137]. First, functionalized 2,4-dichlorothieno[3,2-*d*]pyrimidine **13** with *Boc*-protected piperidin-4-ol and then modified it sequentially to obtain compound **118** (Scheme 26). Compound **118** showed potent inhibition of EGFR^T790M/L858R^ (IC_50_ = 4.90 nM) and high selectivity over EGFR^WT^ (IC_50_ = 359.33 nM, SI = 73.33), surpassing Osimertinib. It also exhibited strong antiproliferative activity against H1975 cells (IC_50_ = 0.62 µM) and low cytotoxicity toward normal L02 and HBE cells, likely due to the presence of an *N*-methylpiperazinyl group at C-2. Thus, **118** is a promising candidate for NSCLC therapy.

### 3.3. PI3K-Targeted Anticancer Activity

**Scheme 27:**
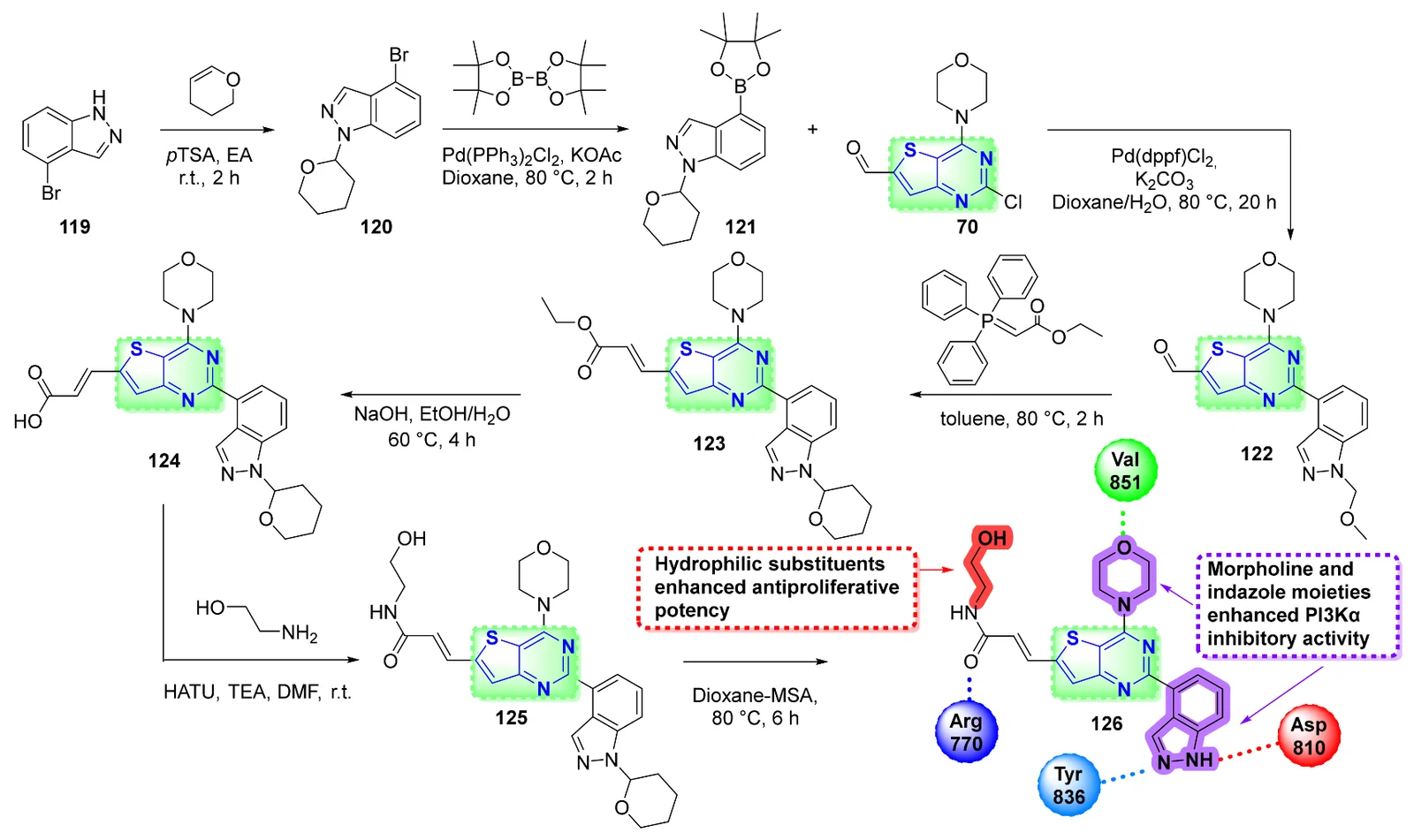
Structure and synthetic route of lead compound **126**.

Problems with the phosphoinositide 3-kinase (PI3K) signaling pathway often occur in human cancers, leading to unchecked cell growth, survival, and resistance to treatment [138]. As a result, small-molecule PI3K inhibitors have become a key group of targeted cancer drugs [139]. They show promising results in slowing cancer cell growth, both when used alone and with other treatments that target specific pathways. Liu et al. [140] synthesized a series of 2,6-disubstituted 4-(thieno[3,2-*d*]pyrimidin-4-yl)morpholine derivatives as selective PI3Kα inhibitors. The synthetic route started from 4-bromo-1*H*-benzimidazole **119**, which was *N*-protected and converted into the corresponding boronic ester. A Suzuki cross-coupling with the thienopyrimidine core afforded intermediate **122**, followed by esterification, amidation, and deprotection steps to yield the final lead compound **126** (Scheme 27). This molecule showed potent PI3Kα inhibitory activity (IC_50_ = 0.039 μM), attributed to hydrogen-bonding interactions of the aminoethanol group with active-site residues. Lead derivative **126** also demonstrated significant antiproliferative potency against MDA-MB-453 cells (IC_50_ = 0.93 μM), exhibiting low cytotoxicity in HEK-293T cells (IC_50_ = 28.18 μM). SAR analysis revealed that the morpholine and indazole moieties were crucial for PI3Kα inhibitory activity, and the incorporation of hydrophilic substituents further enhanced antiproliferative potency.

**Scheme 28:**
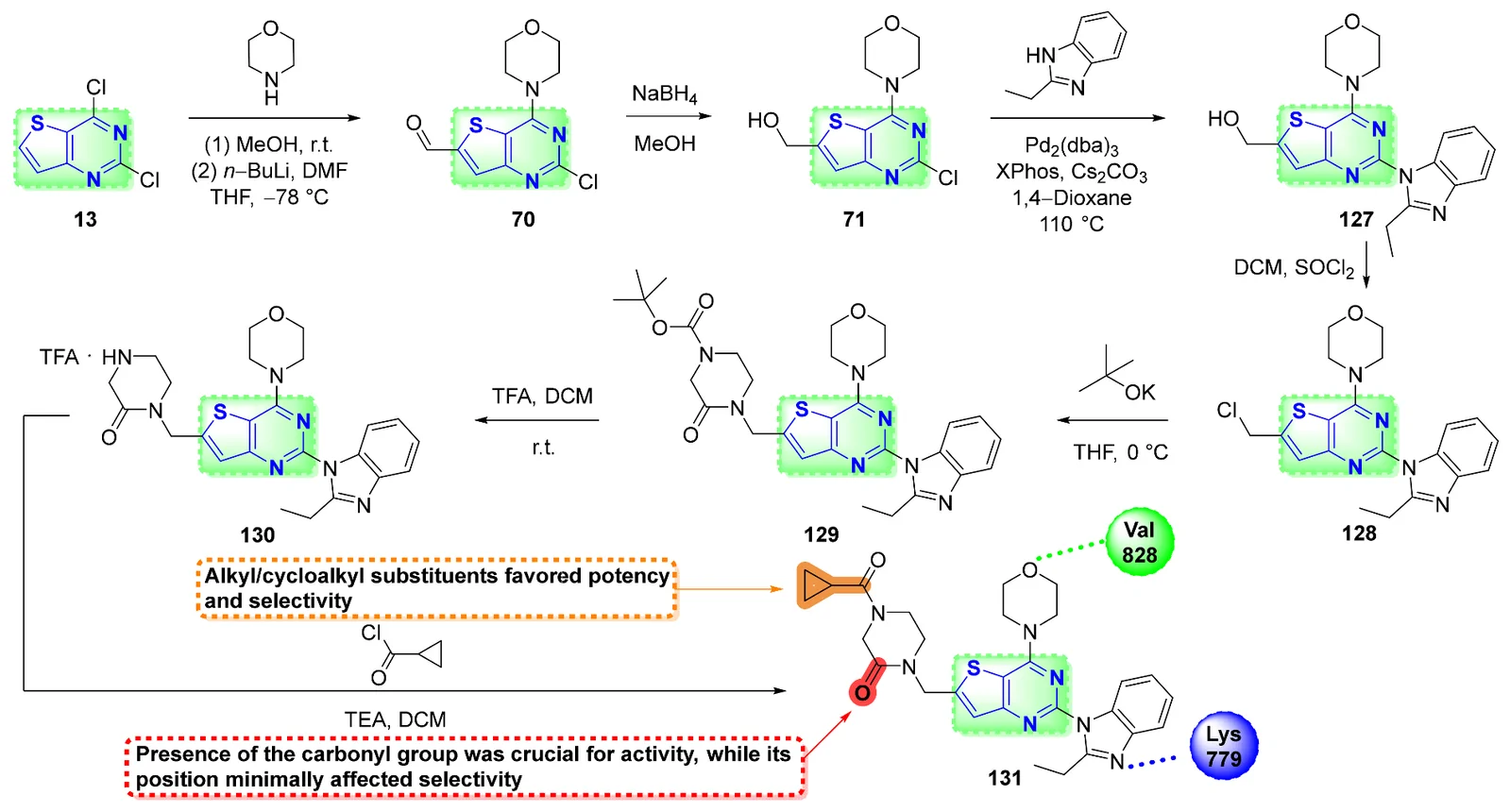
Structure and synthetic route of lead compound **131**.

Phosphoinositide 3-kinase delta (PI3Kδ) inhibitors have emerged as promising targeted anticancer agents due to their ability to selectively suppress malignant B-cell proliferation, survival, and immune signaling pathways [141,142,143]. Wang et al. [144] explored piperazinone-containing thieno[3,2-*d*]pyrimidines as PI3Kδ inhibitors. They identified compound **131**, which has a selectivity index greater than 100 for the delta/beta and delta/gamma isoforms. They evaluated the antiproliferative effects using MTS assays on OCI-LY-3, OCI-LY-10, SU-DHL-6, and JEKO-1. The results showed that **131** had IC_50_ values ranging from 1.1 nM to 2.2 µM, outperforming other compounds. A detailed SAR study revealed that the 1-(thieno[3,2-*d*]pyrimidin-6-ylmethyl)piperazin-2-one derivative exhibited greater potency and selectivity for PI3Kδ (IC_50_ = 1.1 nM). In addition, short alkyl/cycloalkyl substituents provided optimal potency and selectivity, whereas the carbonyl group was essential for activity, but its position had minimal influence on selectivity. The synthesis of compound **131** involved several steps, beginning with nucleophilic substitution and ending with acylation to yield the final product (Scheme 28).

**Scheme 29:**
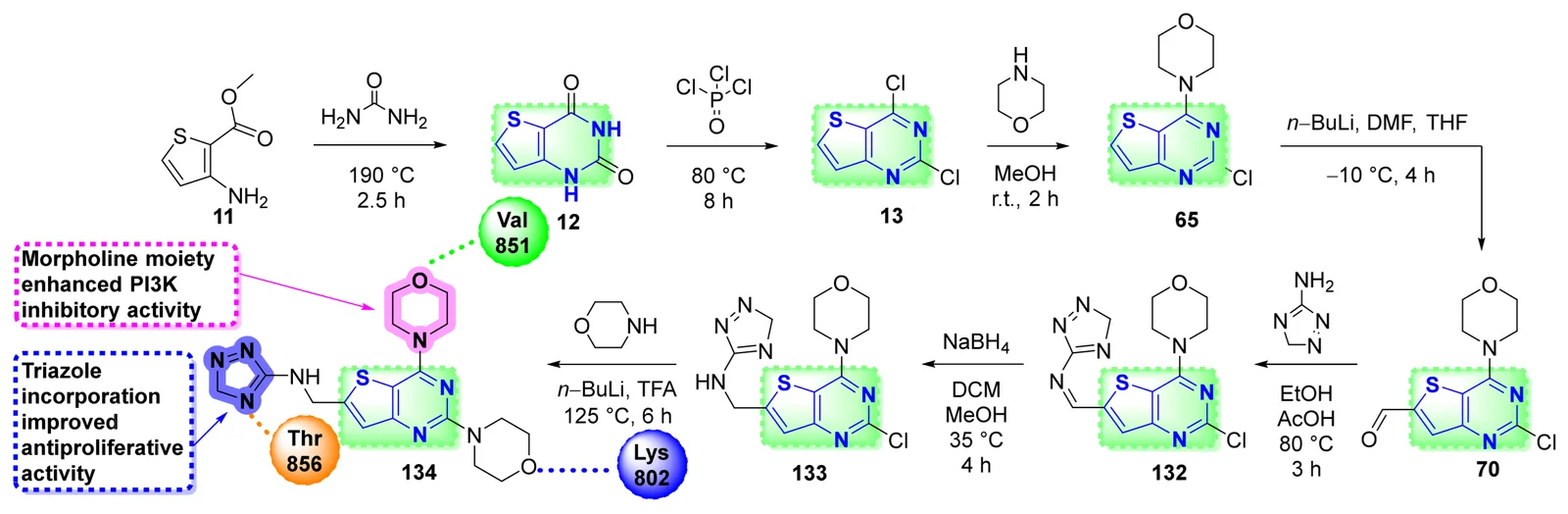
Structure and synthetic route of lead compound **134**.

Phosphoinositide 3-kinase alpha (PI3Kα) inhibitors have gained considerable interest as anticancer agents because they can effectively block cancer cell growth and survival by targeting abnormal PI3K/Akt/mTOR signaling [145,146,147]. Ye et al. [148] designed and synthesized the lead compound **134**, a 2,4-bismorpholinyl-substituted thieno[3,2-*d*]pyrimidine derivative, via a multistep sequence starting from methyl 3-aminothiophene-2-carboxylate. Key steps included urea-mediated cyclization, chlorination, and successive morpholine substitutions with n-BuLi-promoted formylation. Coupling with substituted 3*H*-1,2,4-triazol-5-amine, followed by NaBH_4_ reduction and final morpholine substitution, yielded **134** (Scheme 29). This compound showed potent antiproliferative activity, particularly against HCT116 (IC_50_ = 3.24 µM), and was more effective than other analogs across multiple cell lines. SAR analysis revealed that morpholine incorporation was important for PI3K inhibitory activity, while introduction of the triazole ring enhanced antiproliferative potency. It is suggested that PI3Kα (1 µM, 92.4%) is the likely target, and apoptosis assays confirmed dose-dependent induction of cell death by **134**.

**Scheme 30:**
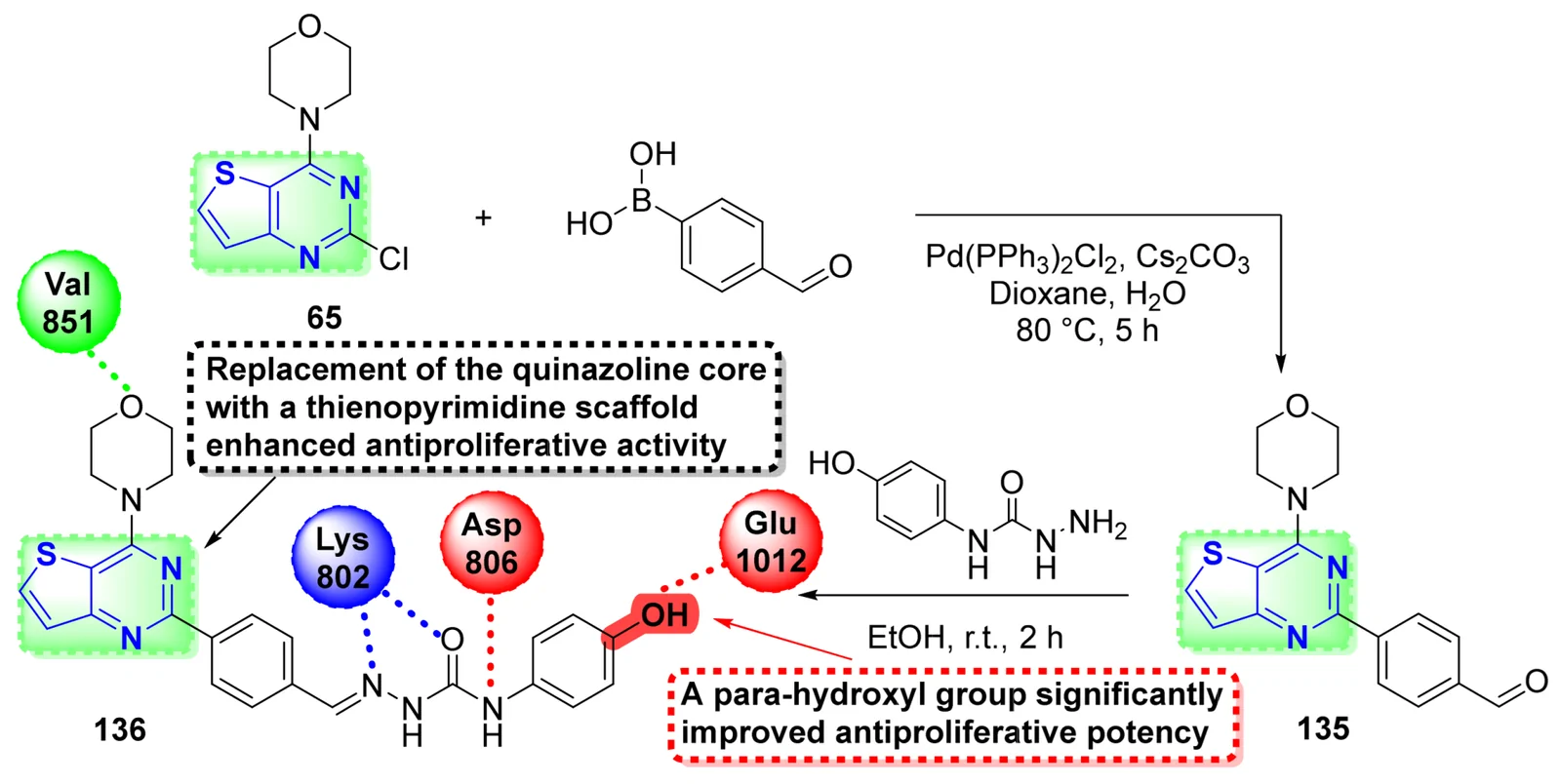
Structure and synthetic route of lead compound **136**.

Hu and co-workers [149] synthesized a series of thieno[3,2-*d*]pyrimidine-based derivatives, including lead compound **136**, via a multistep route starting from methyl-3-aminothiophene-2-carboxylate. Key transformations involved urea cyclization, chlorination, Suzuki coupling with 4-formylphenylboronic acid, and final condensation with a hydrazine-derived intermediate (Scheme 30). Compound **136** demonstrated strong cytotoxic activity against several cancer cell lines, with the highest activity in HCT-116 cells (IC_50_ = 0.22 µM). It effectively inhibited the PI3Kα enzyme (IC_50_ = 0.20 µM) and induced apoptosis and cell-cycle arrest in G1 in cancer cells. SAR analysis revealed that replacement of the quinazoline core with a thienopyrimidine scaffold enhanced antiproliferative activity, while para-hydroxyl substitution further improved potency. Docking studies supported that compound **136** fits well into the active site of PI3Kα, suggesting it could be a selective and promising anticancer candidate.

**Scheme 31:**
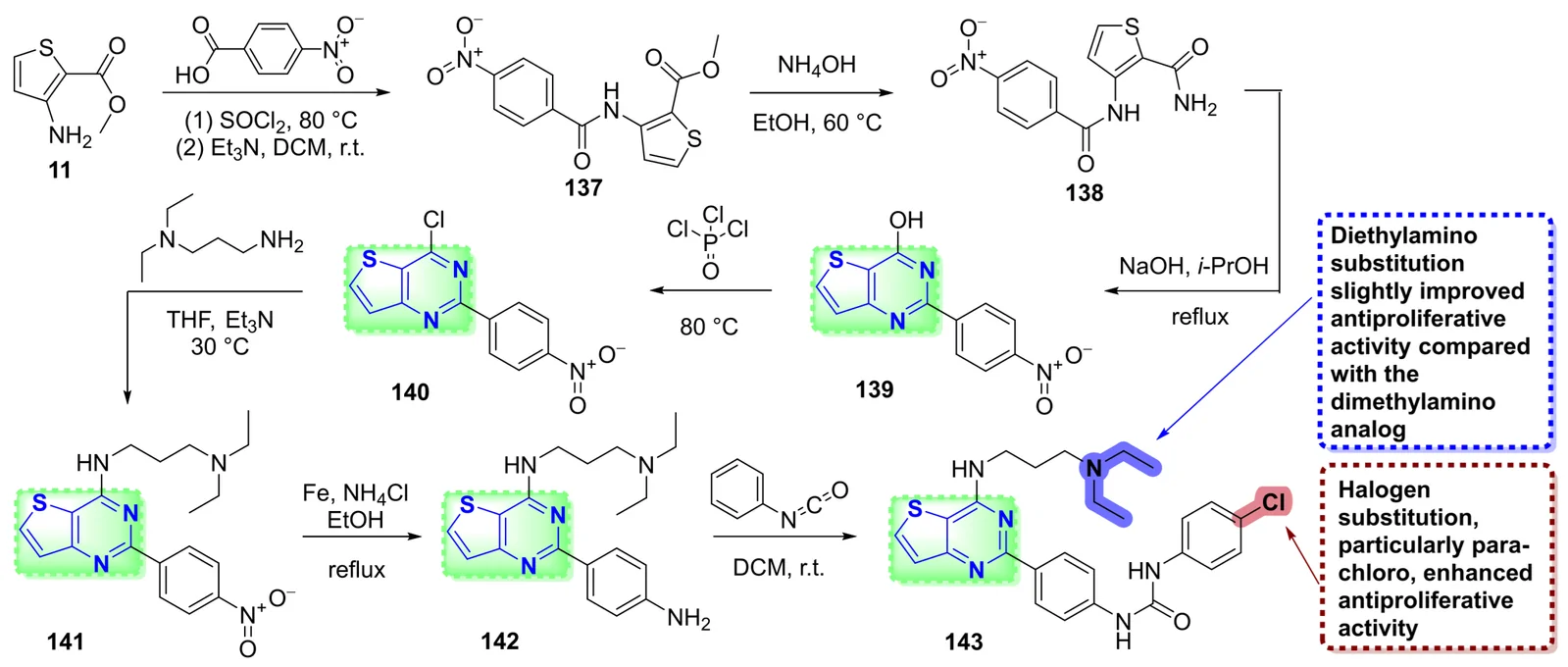
Structure and synthetic route of lead compound **143**.

Two series of thieno[3,2-*d*]pyrimidine derivatives incorporating di-arylurea and C-4 amine fragments were developed by Liu et al. [150]. The synthesis began with the condensation of 4-nitrobenzoic acid and a thiophene derivative, followed by hydroxylamination and base-mediated cyclization to obtain compound **139**. After the chlorination of compound **139**, the formed chloro-derivative was reacted with *N*, *N*-diethylpropane-1,3-diamine to afford intermediate **141**, which, following nitro group reduction, underwent urea formation with isocyanatobenzene to give the final product **143** (Scheme 31). Lead compound **143** exhibited potent antiproliferative activity in H460, HT-29, MKN-45, and MDA-MB-231 cell lines, with IC_50_ values between 0.058 and 0.23 µM. SAR analysis revealed that diethylamino substitution slightly improved antiproliferative activity compared with the dimethylamino analog, while halogen substitution on the phenyl ring, particularly para-chloro, significantly enhanced potency. It should be noted that this derivative also shows marked selectivity for PI3Kα (IC_50_ = 0.13 µM) and induces apoptosis more effectively than reference inhibitors, a result likely influenced by the presence of the DEAPA group at the C-4 position.

**Scheme 32:**
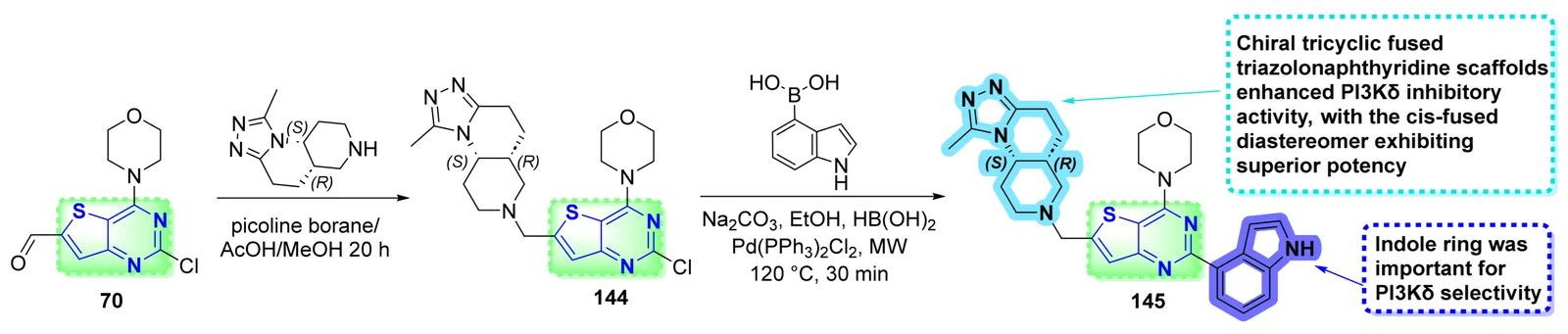
Structure and synthetic route of lead compound **145**.

Schwehm et al. [151] designed and synthesized a series of thieno[3,2-*d*]pyrimidine derivatives via Suzuki–Miyaura cross-coupling, using compound **70** as a key intermediate bearing morpholine groups at C-2 and C-6. Among them, compound **145**, which contains (5aS,9aR)-triazolo [4,3-*a*][1,6]naphthyridine and a (1*H*-indol-4-yl) moiety, emerged as the most potent analogue (Scheme 32). Compound **145** showed strong inhibitory activity against PI3Kδ with an IC_50_ of ~20 nM, while its activity toward PI3Kα, β, and γ remained significantly lower (IC_50_ ≈ 4 µM, 800 nM, and 3.2 µM, respectively). This translated to high selectivity indices, particularly δ/α = 200 and δ/γ = 158, indicating a clear preference for the δ isoform. SAR analysis revealed that the chiral fused triazolonaphthyridine scaffold enhanced PI3Kδ inhibitory activity, with the cis-fused diastereomer showing superior potency, while indole incorporation improved PI3Kδ selectivity. Such a profile highlights compound **145** as a promising PI3Kδ-selective inhibitor with potential therapeutic relevance.

**Scheme 33:**
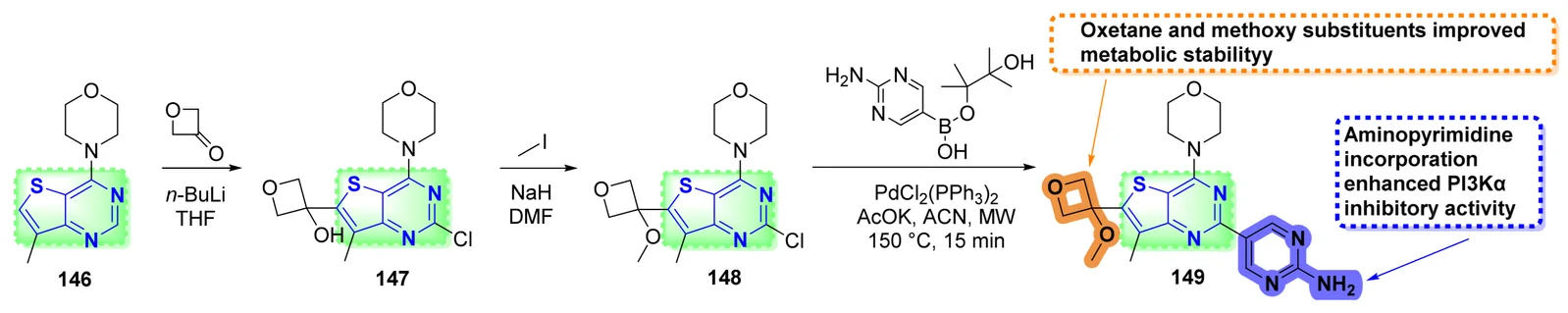
Structure and synthetic route of lead compound **149**.

A series of thienopyrimidine-based derivatives incorporating morpholine and pyrimidine moieties was synthesized by Heffron et al. [152], which preparation started from morpholinopyrimidine **146** and oxetan-3-one to yield intermediate **148**, followed by Suzuki coupling to afford the target compound **149** (Scheme 33). Kinase profiling against 59 kinases (excluding class I PI3Ks) revealed selective inhibition of PI3KC2β (77% at 1 μM). Compound **149** also demonstrated potent cytotoxicity in glioblastoma cell lines, with EC_50_ values as low as 0.14 μM in LN-229 cells. SAR analysis revealed that oxetane and methoxy substituents improved metabolic stability and reduced efflux, while aminopyrimidine incorporation enhanced PI3Kα inhibitory activity. In vivo studies established 40 mg/kg as the optimal oral dose based on tolerability. These findings suggest that compound **149** may serve as a promising PI3KC2β-targeted agent for the treatment of glioblastoma.

### 3.4. Tubulin-Targeted Anticancer Activity

**Scheme 34:**
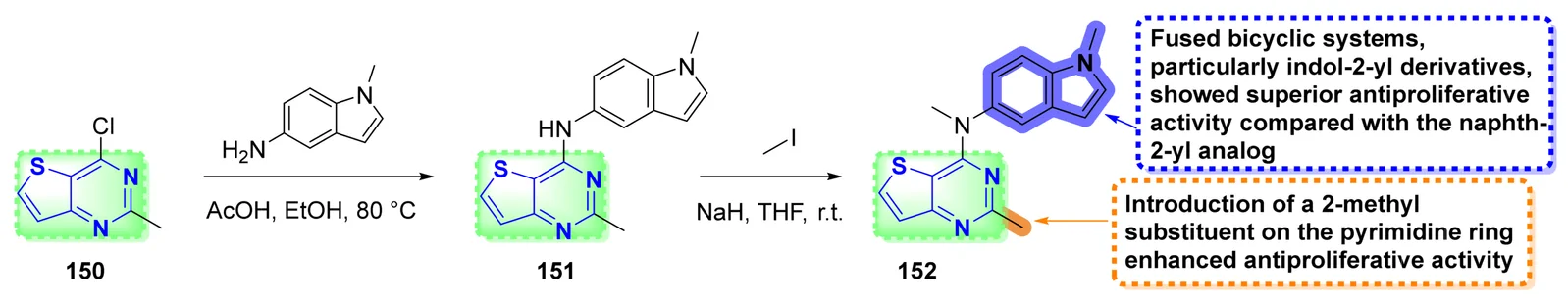
Structure and synthetic route of lead compound **152**.

Tubulin-targeted anticancer agents exhibit potent antiproliferative activity by disrupting microtubule dynamics, leading to mitotic arrest and apoptosis in cancer cells [153,154]. Xu et al. [155] investigated a series of methylated thieno[3,2-*d*]pyrimidines for their antiproliferative and tubulin-inhibitory activity. The lead compound **152** was synthesized via sequential amination and methylation steps (Scheme 34). In vitro assays revealed that **152** exhibited superior potency compared to colchicine across multiple cancer cell lines, including HCT116 (IC_50_ = 0.38 nM), B16–F10 (11.69 nM), HeLa (5.37 nM), and MCF7 (9.53 nM), with 1.1–24.7-fold higher activity. It also showed low-nanomolar IC_50_ values against H23 and HepG2 cells. SAR analysis revealed that fused bicyclic systems, particularly indol-2-yl derivatives, significantly enhanced antiproliferative activity, while a 2-methyl substituent on the pyrimidine ring further improved potency. Tubulin inhibition by **152** was confirmed in biochemical assays (IC_50_ = 2.4 μM). During animal testing with the B16–F10 tumor model, treated groups showed noticeable delays in tumor progression. The addition of NP-19 to the regimen further improved the response.

**Scheme 35:**
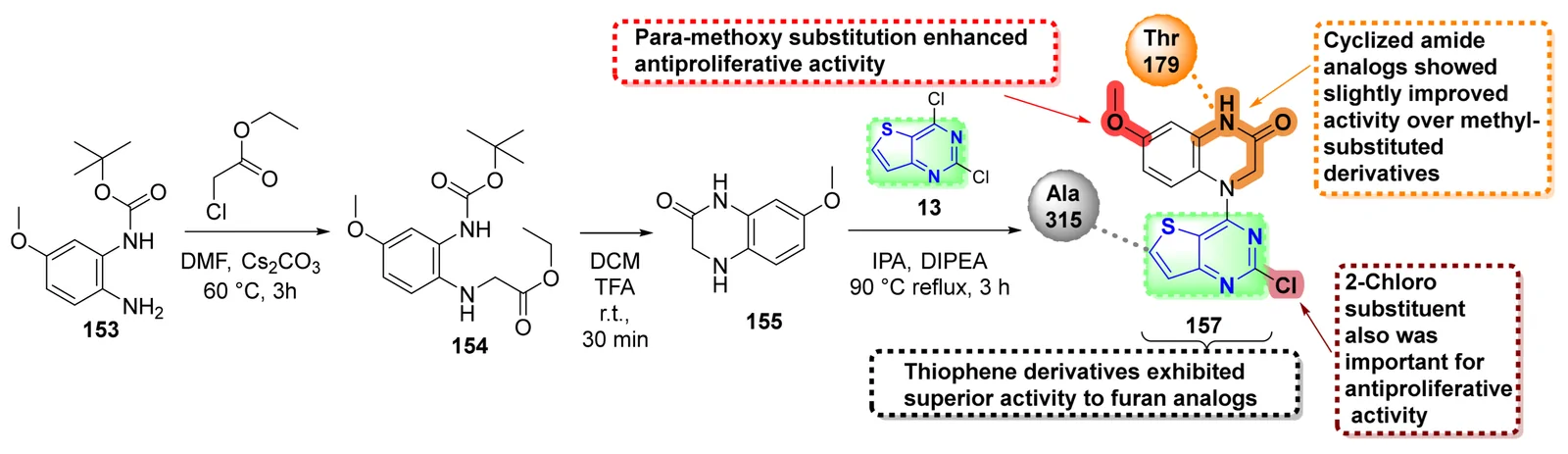
Structure and synthetic route of lead compound **157**.

Wu et al. [156] synthesized a series of thieno[3,2-*d*]pyrimidine-based derivatives incorporating various heterocyclic moieties, and obtained X-ray co-crystal structures with tubulin for compound **157**. The synthesis of the lead molecule followed a three-step sequence: ethyl acetate substitution at position 9, cyclization, and insertion of a thienopyrimidine unit to form compound **157** (Scheme 35). In proliferation assays, **157** demonstrated potent inhibitory activity against A549, A2780, SKOV3, and HCC827 cell lines, with IC_50_ values of 0.84, 0.38, 0.31, and 0.34 nM, respectively, exceeding the activity of CA-4 by up to 6.6-fold. SAR analysis revealed that para-methoxy and 2-chloro substitutions enhanced antiproliferative activity, while cyclized analogs showed slightly improved potency compared with methyl-substituted derivatives. Moreover, thiophene derivatives exhibited superior activity compared with furan and pyrrole analogs. The resistance index (1.97) was significantly lower than that of colchicine and paclitaxel. Compound **157** also suppressed colony formation in SKOV3 cells, induced G2/M arrest, and promoted apoptosis. Tubulin polymerization inhibition was confirmed through binding studies (KD = 24.37 μM), supporting its potential as a candidate for ovarian cancer therapy.

**Scheme 36:**
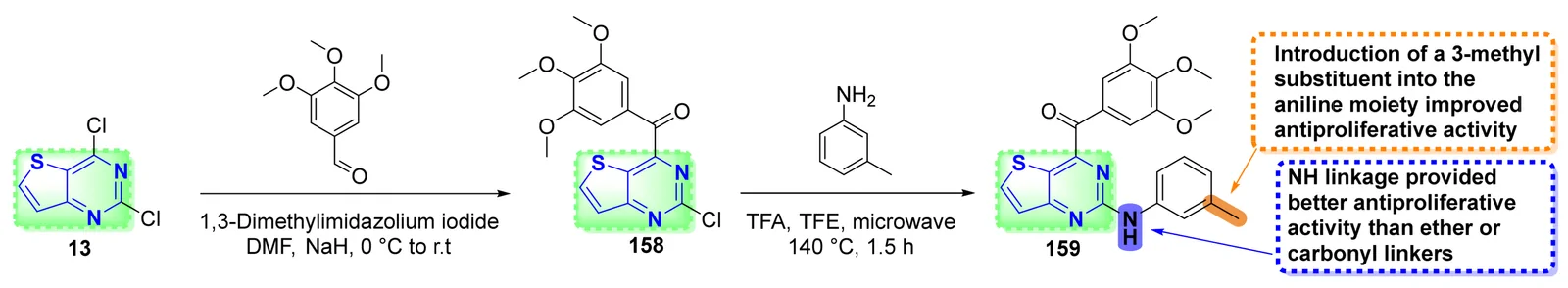
Structure and synthetic route of lead compound **159**.

Tian et al. [157] synthesized compound **159** by introducing 3,4,5-trimethoxybenzoyl and *m*-methylaniline onto the thieno[3,2-*d*]pyrimidine core (Scheme 36). Compound **159** showed strong antiproliferative activity in HL60, HCT116, A549, MCF-7, and HeLa cells (IC_50_ = 34–196 nM). It inhibited tubulin polymerization (IC_50_ = 4.1 µM) and induced dose-dependent apoptosis in HeLa cells, with late-apoptotic rates reaching 78.7% at 500 nM. SAR analysis revealed that electron-donating substituents, particularly a 3-methyl group, increased the antiproliferative activity. The NH linker was also found to be more effective than ether and carbonyl linkages.

### 3.5. c-Met-Targeted Anticancer Activity

**Scheme 37:**
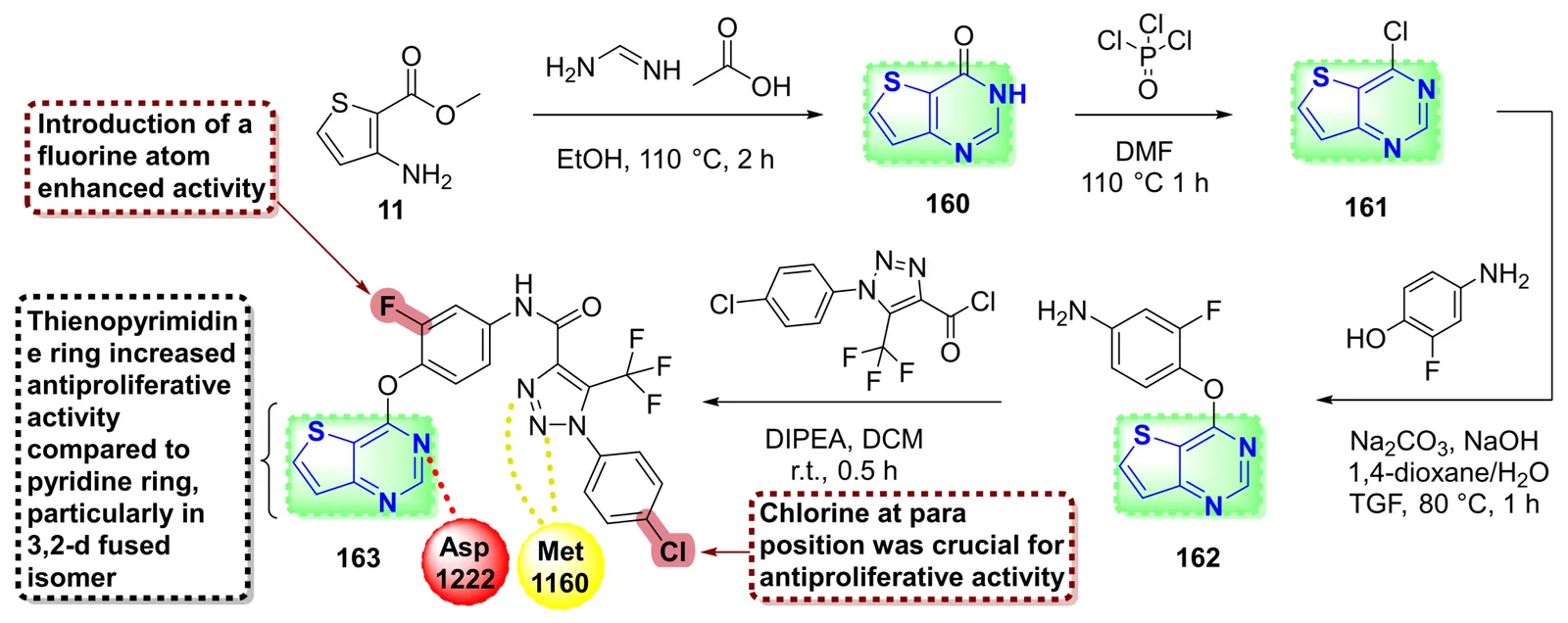
Structure and synthetic route of lead compound **163**.

c-Met inhibitors are considered promising anticancer agents because they can effectively inhibit tumor growth, invasion, and metastasis by targeting abnormal c-Met signaling [158]. Wang et al. [159] developed a series of thienopyrimidine-based derivatives incorporating 1,2,3-triazole and *N*-methylpicolinamide moieties. The synthesis of lead compound **163** began from methyl 3-aminothiophene-2-carboxylate, which underwent cyclization with formamidine acetate and chlorination with POCl_3_ to yield intermediate **161**. Subsequent substitution with 2-fluoro-4-aminophenol provided **162**, followed by acylation with a triazole-containing acid chloride to afford the target compound (Scheme 37). Compound **163** exhibited potent cytotoxicity in A549, HepG2, and MCF-7 cells (IC_50_ = 0.5–1.0 µM) and selectively inhibited c-Met kinase (IC_50_ = 16 nM), with minimal activity against other kinases. The SAR analysis showed that adding fluorine and para-chloro groups increased antiproliferative activity. The thieno[3,2-*d*]pyrimidine scaffold was also more potent than the pyridine analogs. These findings indicate its promise as a selective *c*-Met inhibitor for anticancer therapy.

**Scheme 38:**
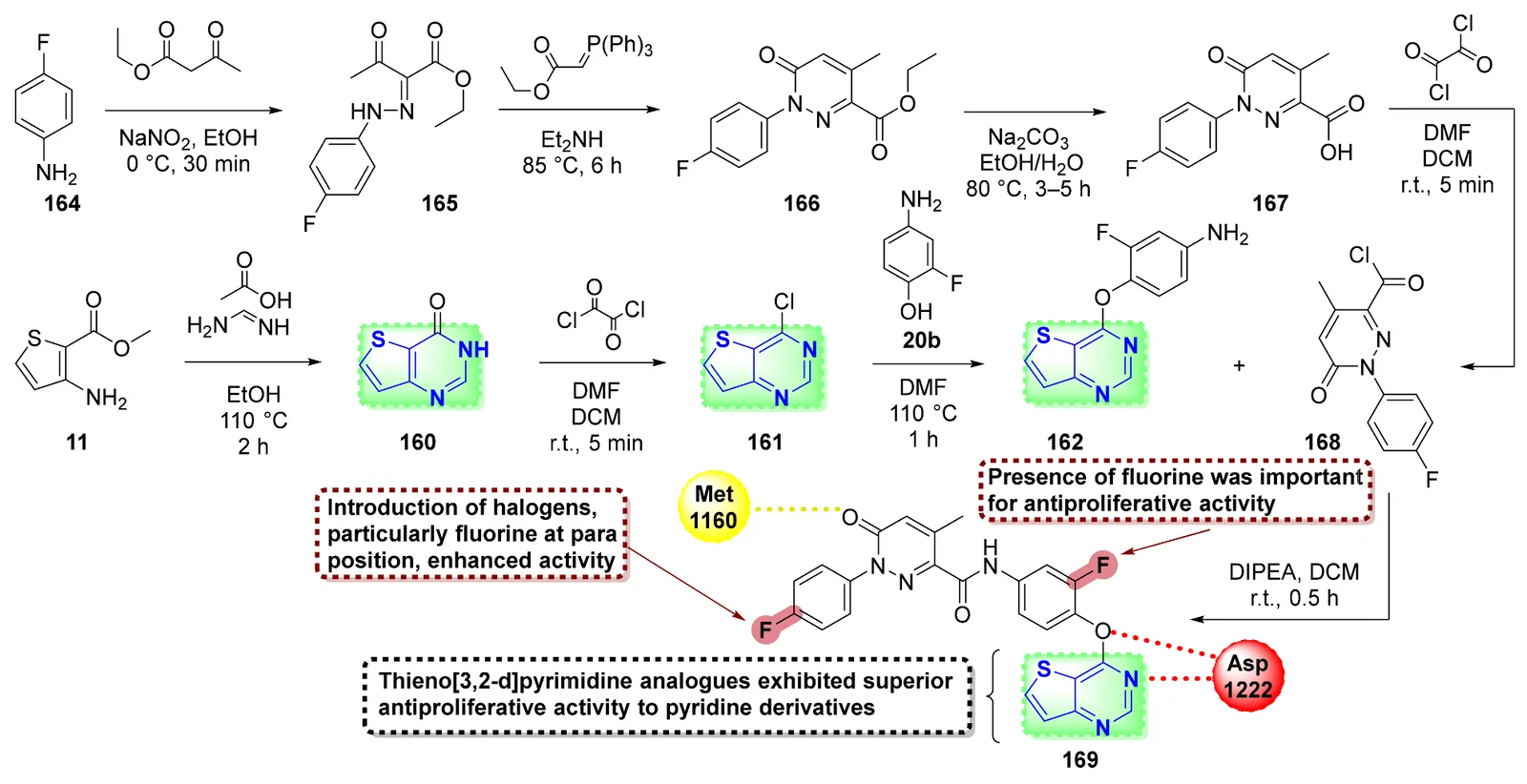
Structure and synthetic route of lead compound **169**.

Wang et al. [160] synthesized a series of pyridazinone-modified thieno[3,2-*d*]pyrimidine derivatives through a multistep synthetic route. The synthesis involved diazotization of 4-fluoroaniline, formation of key pyridazinone intermediates, and coupling with a thieno[3,2-*d*]pyrimidine scaffold to yield the lead compound **169** (Scheme 38). This molecule exhibited vigorous antiproliferative activity against A549, HepG2, and MCF-7 cells (IC_50_ = 0.47–0.74 µM) and selectively inhibited c-Met kinase (IC_50_ = 19 nM) over related kinases. Moreover, **169** induced late apoptosis in a concentration-dependent manner, suggesting its potential as a c-Met-targeted anticancer agent. SAR analysis revealed that para-fluoro substitution enhanced antiproliferative activity, while the thieno[3,2-*d*] fused scaffold exhibited superior potency compared with the pyridine analog.

### 3.6. ATR-Targeted Anticancer Activity

**Scheme 39:**
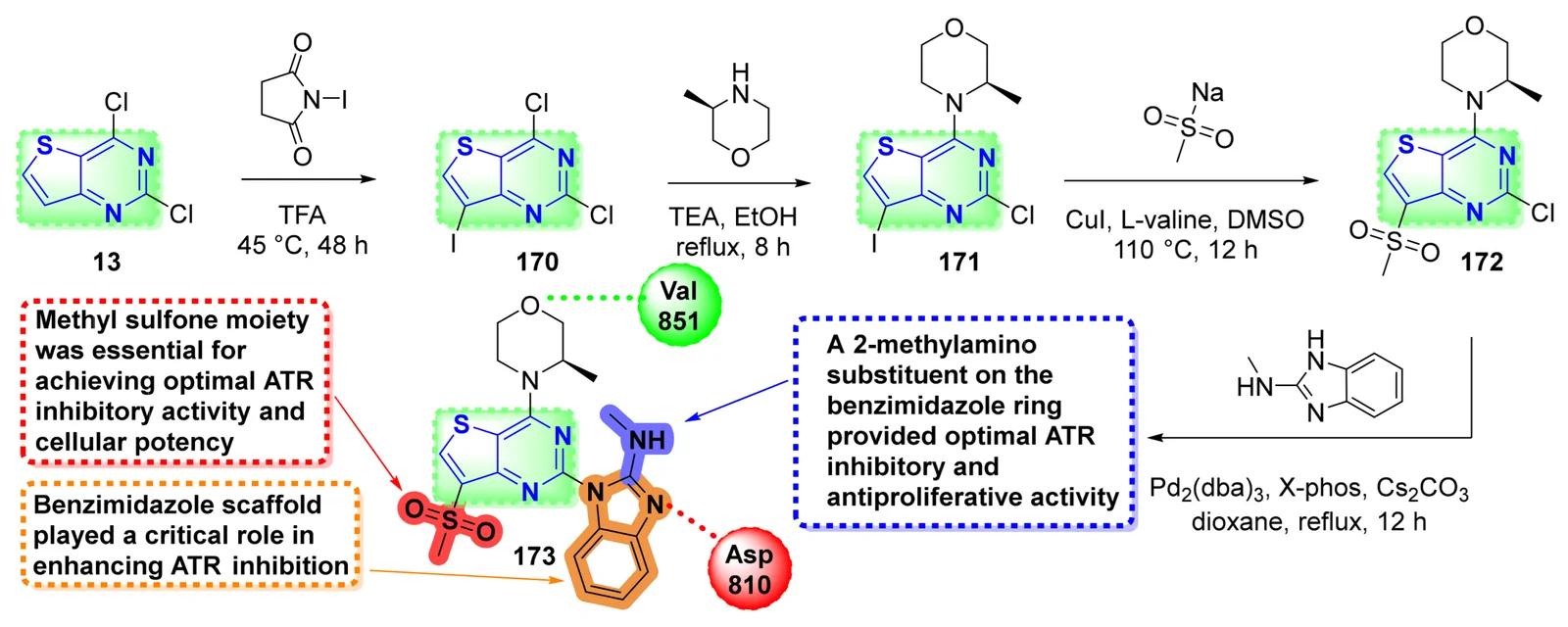
Structure and synthetic route of lead compound **173**.

ATR kinase-targeted anticancer agents have shown strong therapeutic potential by disrupting DNA damage repair and enhancing replication stress-induced cancer cell death [161,162]. Duan et al. [163] designed thieno[3,2-*d*]pyrimidine-based ATR inhibitors using a hybrid approach, leading to compound **173**. Its synthesis included iodination and sulfonation at C-7, morpholine attachment at C-4, and benzimidazole substitution at C-2 (Scheme 39). Compound **173** inhibited ATR with an IC_50_ of 1.5 nM and exhibited vigorous antiproliferative activity in LoVo cells (IC_50_ = 0.073 μM), outperforming AZ20. Binding affinity was attributed to hydrogen bonding from methylamino and benzimidazole groups. In HT-29 cells, the combination with AZD1390 enhanced potency >50-fold. In vivo, **173** achieved 55% TGI at 50 mg/kg without adverse effects, comparable to BAY 1895344. SAR analysis showed that adding methyl sulfone improved ATR inhibitory activity and cellular potency. The benzimidazole scaffold was essential for ATR inhibition. Also, a 2-methylamino group on the benzimidazole ring gave the best ATR inhibition and antiproliferative effects.

**Scheme 40:**
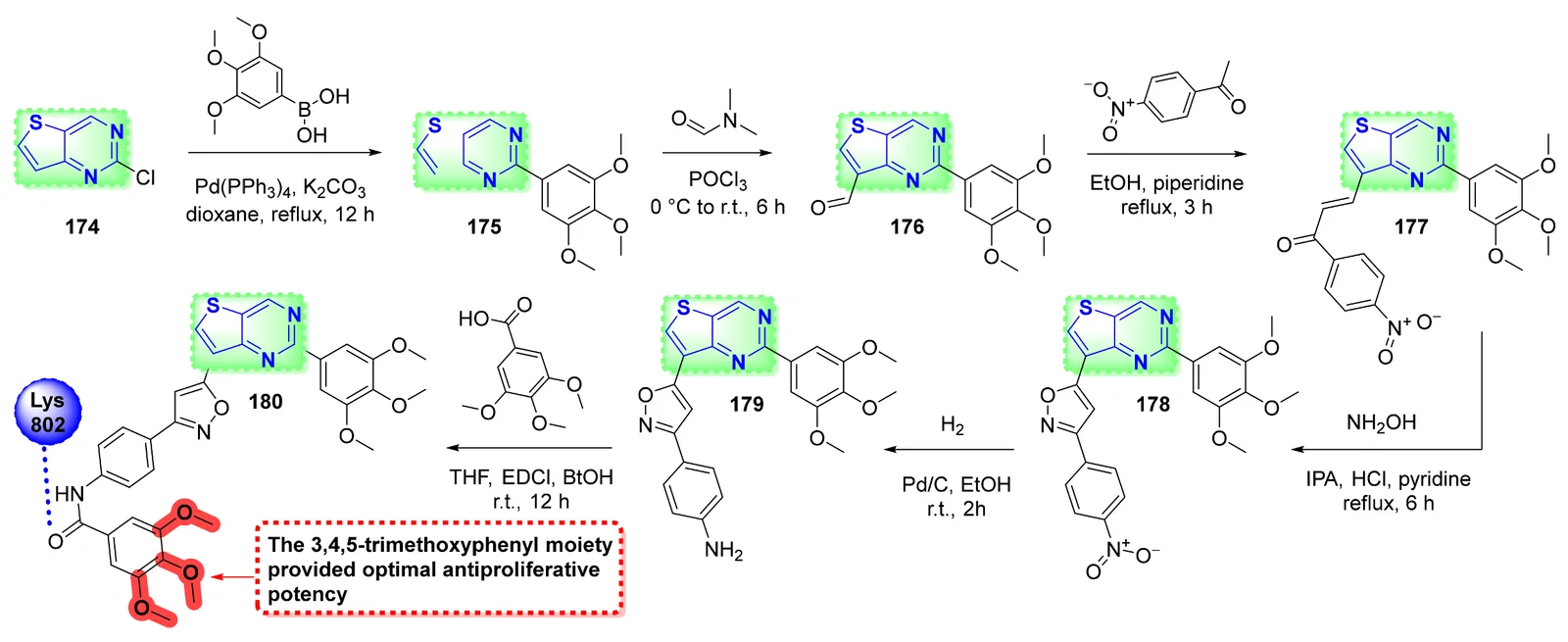
Structure and synthetic route of lead compound **180**.

Babu et al. [164] developed hybrid thienopyrimidine–isoxazole derivatives and identified **180** as the most potent compound. Its synthesis involved Suzuki coupling at C-2, Vilsmeier–Haack formylation at C-7, chalcone formation, isoxazole cyclization, reduction, and amide formation (Scheme 40). Cytotoxic testing of compound **180** gave IC_50_ values of 0.012 μM (PC3), 0.019 μM (MCF-7), 0.11 μM (Colo-205), and 0.87 μM (A549), all superior to etoposide. SAR analysis revealed that increasing methoxy substitution on the phenyl ring enhanced antiproliferative activity, with the 3,4,5-trimethoxy derivative exhibiting the highest potency. Docking analysis indicated binding at the ATR kinase site, consistent with experimental results.

### 3.7. VEGFR-2-Targeted Anticancer Activity

**Scheme 41:**
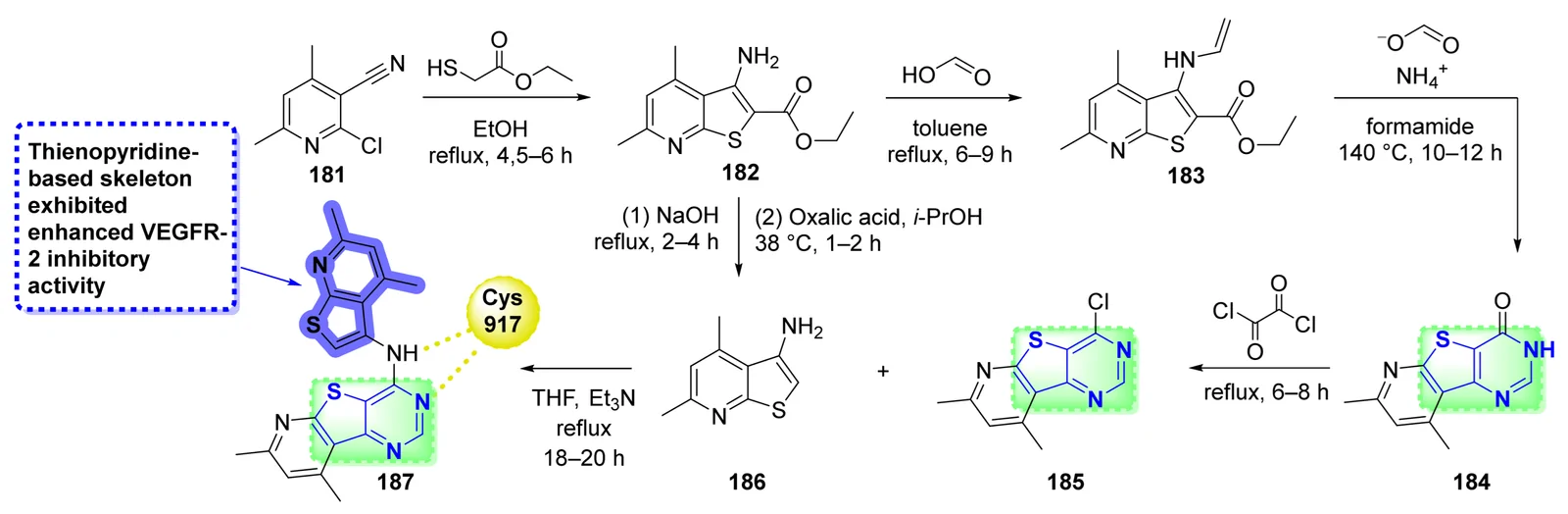
Structure and synthetic route of lead compound **187**.

VEGFR-2-targeted anticancer agents have shown significant potential by inhibiting tumor angiogenesis, thereby suppressing cancer cell growth, metastasis, and tumor progression [165,166,167]. Abdel Aziz et al. [168] synthesized a series of tricyclic pyridothienopyrimidine derivatives bearing bicyclic heteroaryl amines at the C-4 position, inspired by Cediranib, a known VEGFR-2 inhibitor. The scaffold was constructed via a Thorpe–Ziegler cyclization of 2-chloro-4,6-dimethylnicotinonitrile with ethyl-2-mercaptoacetate, followed by formylation and subsequent ring closure using ammonium formate. Key intermediate **185** was chlorinated and coupled with a heteroarylamine to yield the final compound **187** (Scheme 41). In kinase screening, **187** selectively inhibited VEGFR-2 with 67% activity at 10 µM and an IC_50_ of 2.6 µM, while showing minimal effect on EGFR, CDK5, and GSK3 isoforms. SAR analysis revealed that incorporation of the thienopyridine moiety enhanced VEGFR-2 inhibitory activity, likely due to improved hydrophobic interactions and complex stability. The selectivity was attributed to methyl substituents at positions C-7 and C-9, which enhanced hydrophobic interactions with the VEGFR-2 active site.

**Scheme 42:**
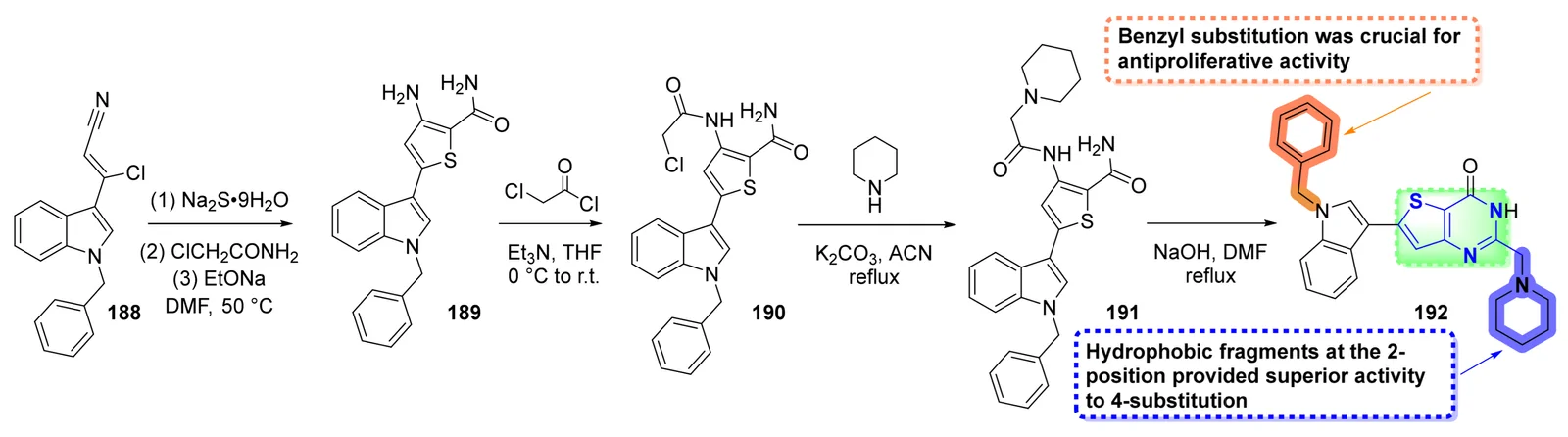
Structure and synthetic route of lead compound **192**.

Perspicace et al. [169] developed a potent thieno[3,2-*d*]pyrimidine-based VEGFR-2 inhibitor, compound **192**, incorporating heteroaryl and piperidine moieties. The synthesis involved a Vilsmeier-induced thiophene formation, followed by condensation with 2-chloroacetamide, chloroacylation, addition of piperidine, and final cyclization (Scheme 42). Compound **192** exhibited potent VEGFR-2 inhibition (99.2%) at 200 µM, with an IC_50_ of 2.25 µM. It also effectively blocked endothelial tube formation, with an EC_50_ of 31 nM, surpassing sunitinib (EC_50_ = 645 nM). The tartaric acid salt of **192** was used to improve aqueous solubility in biological assays. SAR analysis revealed that benzyl substitution was crucial for antiproliferative activity, while hydrophobic substitution at the 2-position of the thienopyrimidine core provided superior potency compared with 4-substitution. These findings highlight **192** as a promising antiangiogenic candidate.

### 3.8. CDK-Targeted Anticancer Activity

**Scheme 43:**
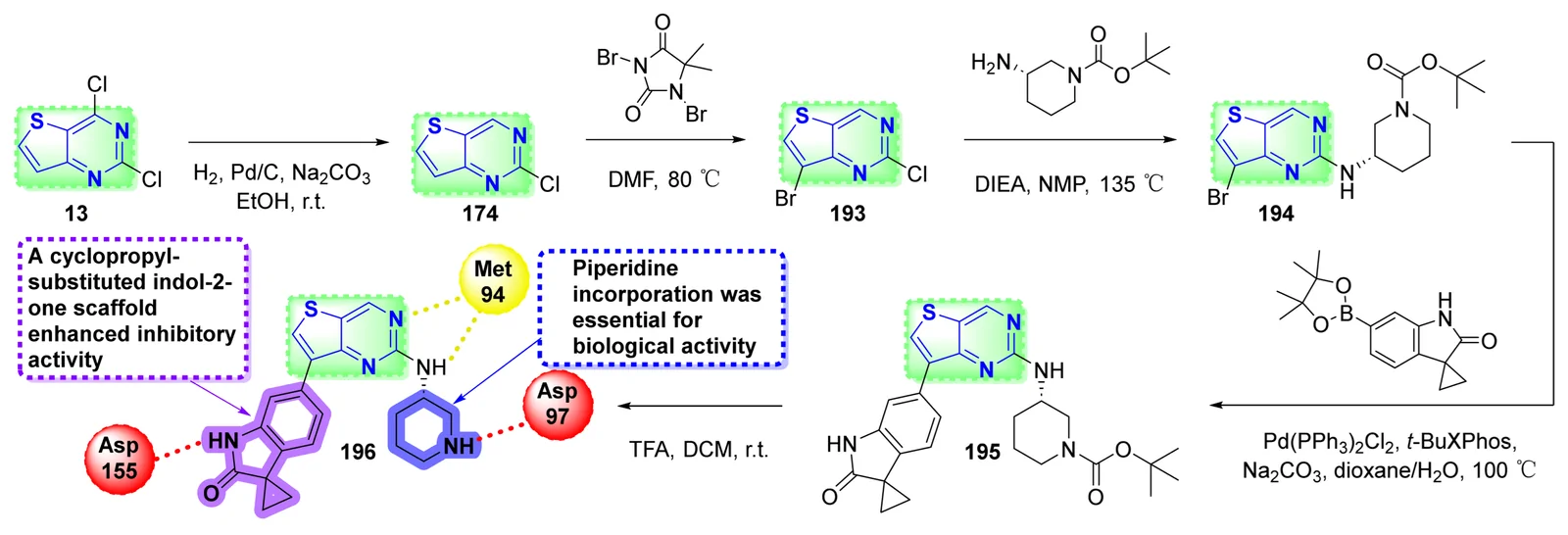
Structure and synthetic route of lead compound **196**.

Cyclin-dependent kinases (CDK)-targeted anticancer agents exhibit potent antitumor activity by blocking cell-cycle progression and suppressing uncontrolled cancer cell proliferation [170,171]. Zhang et al. [172] developed a series of CDK7 inhibitors based on the thieno[3,2-*d*]pyrimidine scaffold through stepwise structural optimization. The synthetic strategy included halogenation, *C*–*N* bond formation, Suzuki coupling at C-7, and Boc group removal, leading to the lead compound **196** (Scheme 43). This molecule showed complete inhibition of the CDK7/CyclinH/MAT1 complex at 100 and 1000 nM concentrations, with an IC_50_ of 1.4 nM. In cytotoxicity assays, compound **196** displayed enhanced potency against MDA-MB-453 cells (EC_50_ = 0.20 μM), outperforming SY-5609. The spiro-cyclopropyl moiety in compound **196** is thought to promote binding by fitting into the hydrophobic pocket near the P-loop region of CDK7. At higher concentrations, this compound reduced phosphorylation of downstream targets including CDK2, RB, and RNA polymerase II, resulting in cell-cycle arrest at the G0/G1 phase and a marked decrease in cell proliferation. Oral bioavailability and supportive pharmacokinetic parameters further reinforce its potential as a selective CDK7-targeted anticancer agent.

**Scheme 44:**
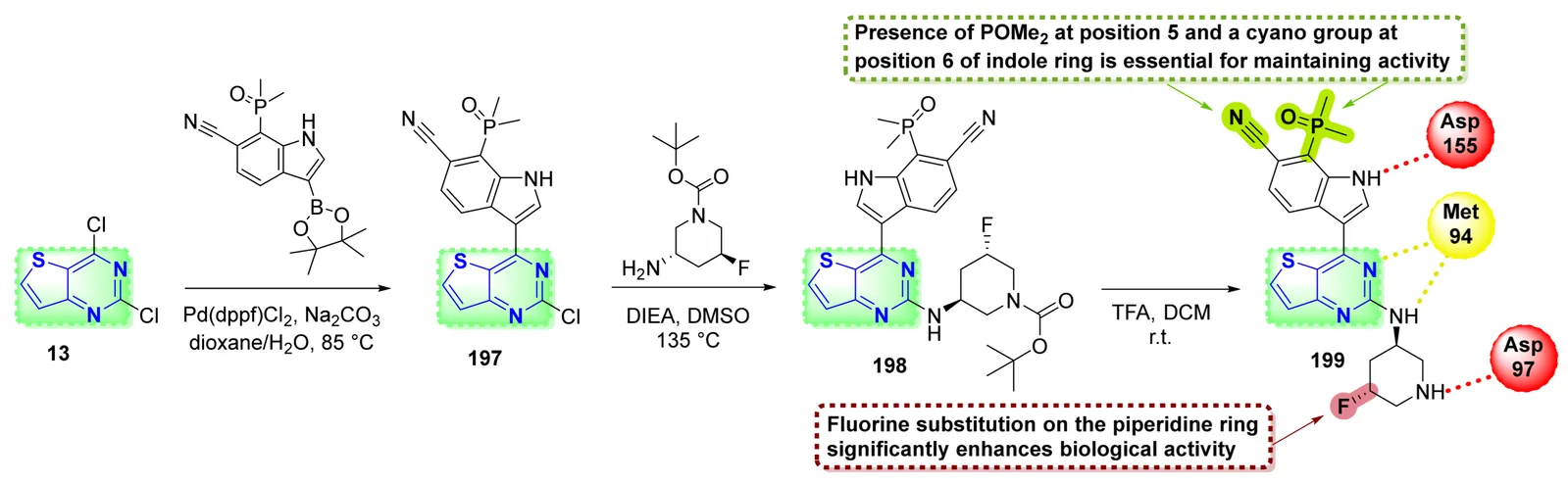
Structure and synthetic route of lead compound **199**.

Zhang et al. [55] synthesized thieno[3,2-*d*]pyrimidine derivatives targeting CDK7 and identified compound **199** as the most active. It was obtained by Suzuki and Buchwald couplings on scaffold **199** (Scheme 44). Compound **199** showed potent inhibition of CDK7/CyclinH/MAT1 (IC_50_ = 6.5 nM) and potent antiproliferative activity in MDA-MB-453 cells (EC_50_ = 0.15 μM), surpassing SY-5609. In vitro metabolism studies indicated moderate stability (T_1/2_ = 72 min in human, 51 min in mouse microsomes). SAR results linked the thiophene core to selectivity and the fluorinated piperidine to metabolic stability. In a TNBC xenograft model, oral treatment with **199** led to tumor regression without evident toxicity.

### 3.9. HDAC-Targeted Anticancer Activity

**Scheme 45:**
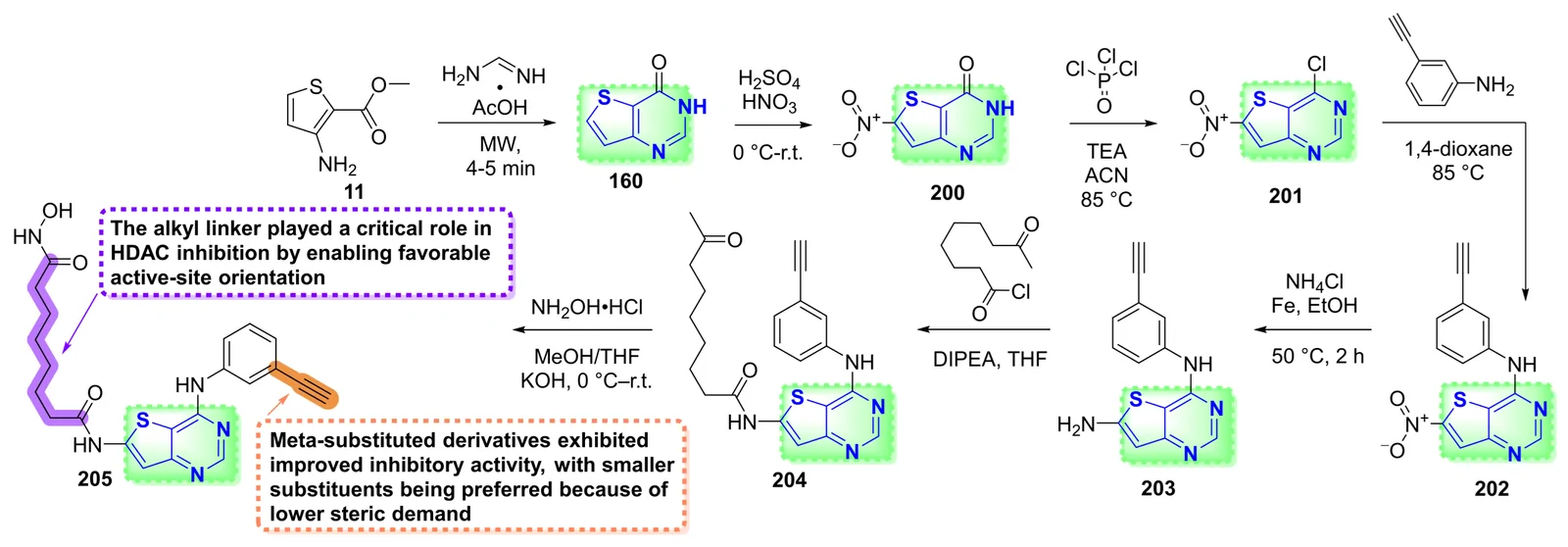
Structure and synthetic route of lead compound **205**.

Histone deacetylases (HDAC)-targeted anticancer agents have shown promising therapeutic potential by regulating gene expression and inducing apoptosis in cancer cells [173,174,175]. Wang and co-workers [176] synthesized a series of thieno[3,2-*d*]pyrimidin-4(3*H*)-one derivatives as potential HDAC inhibitors. The scaffold was constructed via microwave-assisted cyclization, followed by nitration, chlorination, and sequential coupling with 3-ethynylaniline to yield intermediate **203**. Final acylation and oxime formation afforded the target compound **205** (Scheme 45). In enzyme assays, lead compound **205** inhibited HDAC1, HDAC3, and HDAC6 with IC_50_ values of 29.8, 24.7, and 21.3 nM, respectively. It also showed more substantial antiproliferative effects than SAHA in RPMI 8226 and HCT116 cells, with IC_50_ values near 1 µM. At nanomolar levels, **205** significantly increased tubulin acetylation, supporting its potential as a multi-target HDAC inhibitor. SAR analysis indicated that the alkyl linker was essential for HDAC inhibition by enabling favorable positioning within the active site.

**Scheme 46:**
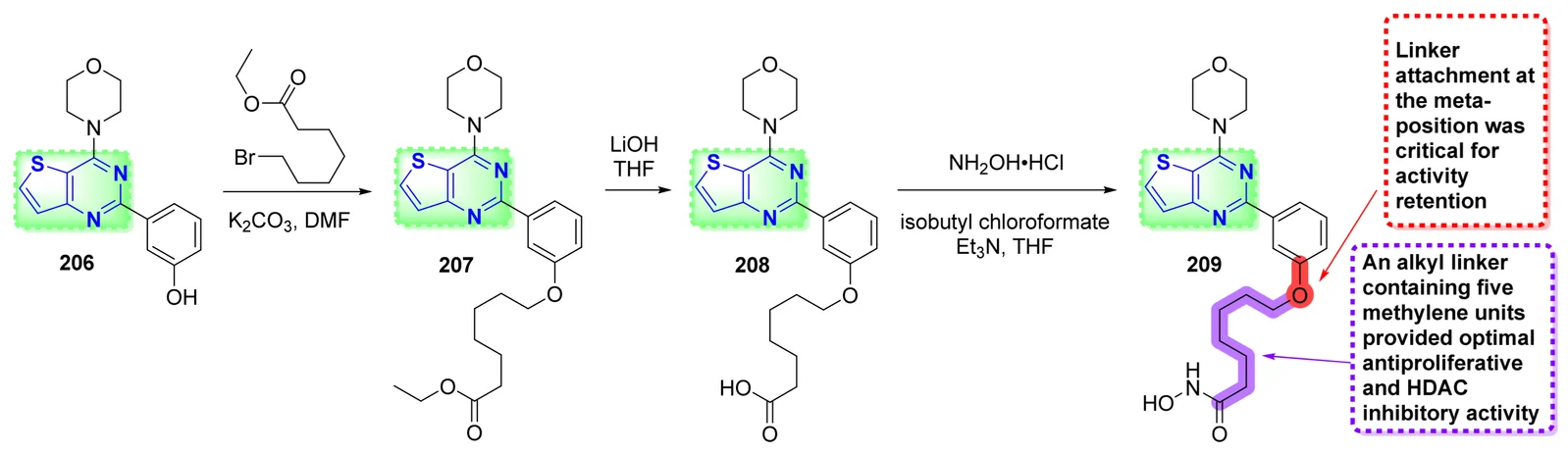
Structure and synthetic route of lead compound **209**.

Tan et al. [177] synthesized a novel thieno[3,2-*d*]pyrimidine derivative **209**, which bears a morpholine ring at C-4 and an aryl group containing a hydroxamic acid at C-2. They accomplished this through the alkylation of intermediate **206** with ethyl 7-bromoheptanoate, followed by hydrolysis and conversion to the hydroxamic acid via reaction with hydroxylamine (Scheme 46). Compound **209** showed potent HDAC inhibition (IC_50_ = 0.38 µM) and cytotoxicity against HCT-116, HeLa, and MCF-7 cells (IC_50_ ≈ 4–4.8 µM). At 1 µM, it inhibited HDAC activity by 76.8%. Flow cytometry revealed G2/M arrest and 44.5% apoptosis in HCT-116 cells at 10 µM. SAR analysis revealed that attaching the linker at the meta-position was critical for maintaining biological activity. Additionally, the length of the alkyl spacer significantly affected potency, with a five-methylene linker demonstrating the most favorable antiproliferative and HDAC-inhibitory activities. Therefore, a meta-substituted aryl group was crucial for activity, suggesting potential for further optimization.

### 3.10. JAK-Targeted Anticancer Activity

**Scheme 47:**
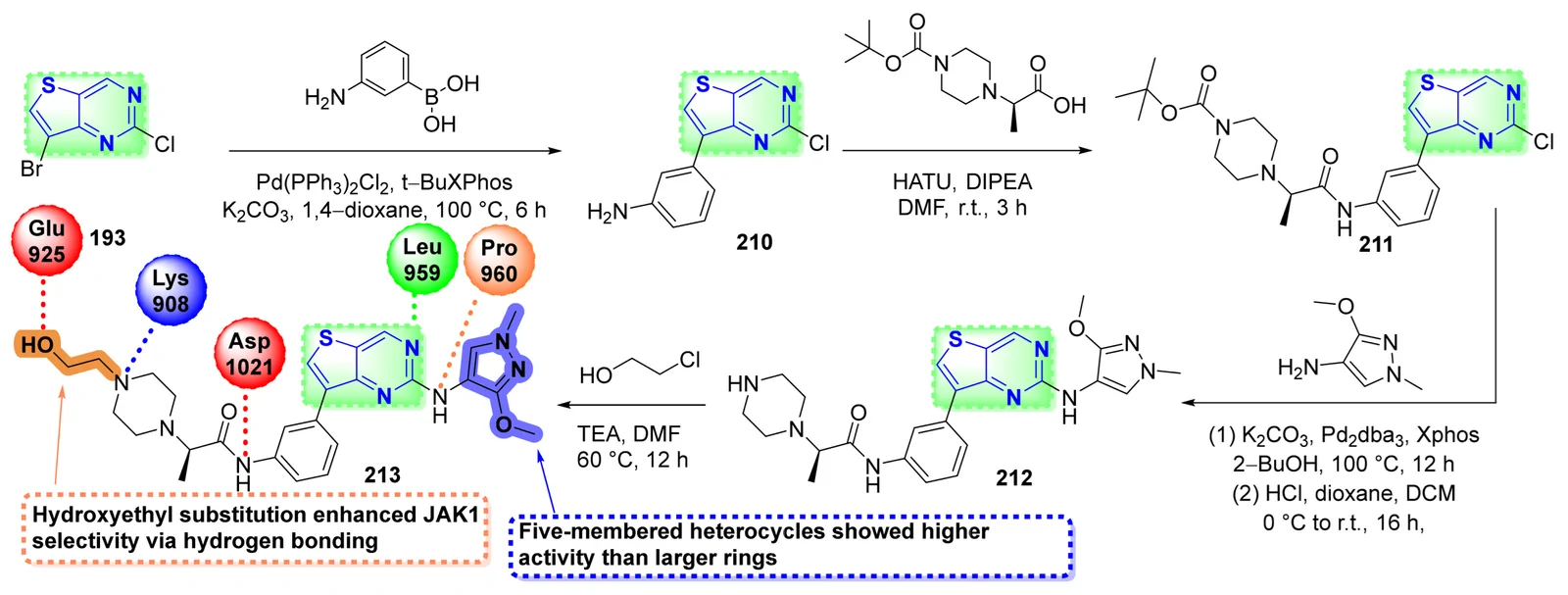
Structure and synthetic route of lead compound **213**.

Janus kinases (JAK)-targeted anticancer agents have attracted considerable interest because they can effectively block abnormal JAK/STAT signaling that promotes cancer cell growth and survival [178,179]. Kim et al. [180] synthesized a series of optically active 2,7-substituted thieno[3,2-*d*]pyrimidines as selective JAK1 inhibitors using a scaffold-morphing approach. Starting from commercially available core **193**, the C-7 position was modified via Suzuki coupling and amidation, followed by Buchwald coupling at C-2, Boc deprotection, and condensation with *N*-ethanolamine to yield the target compound **213** (Scheme 47). This molecule exhibited potent and selective inhibition of JAK1 (IC_50_ = 0.022 μM), with markedly lower activity against JAK2 and JAK3. Selectivity ratios (JAK2/JAK1 = 35, JAK3/JAK1 = 73) confirmed strong isoform preference. Compound **213** also showed notable activity in JAK1-TEL Ba/F3 cells (IC_50_ = 0.193 μM) and moderate effects in EGFR- and KRAS-mutant lines, while sparing parental Ba/F3 cells. SAR analysis showed that the hydroxyethyl substituent improves JAK1 selectivity, likely due to additional hydrogen-bonding interactions in the active site. Five-membered pyrazole moieties demonstrated greater activity than larger rings, indicating that reduced steric bulk supports more effective target binding. These findings support the development of compound **213** as a promising JAK1-targeted anticancer agent.

**Scheme 48:**
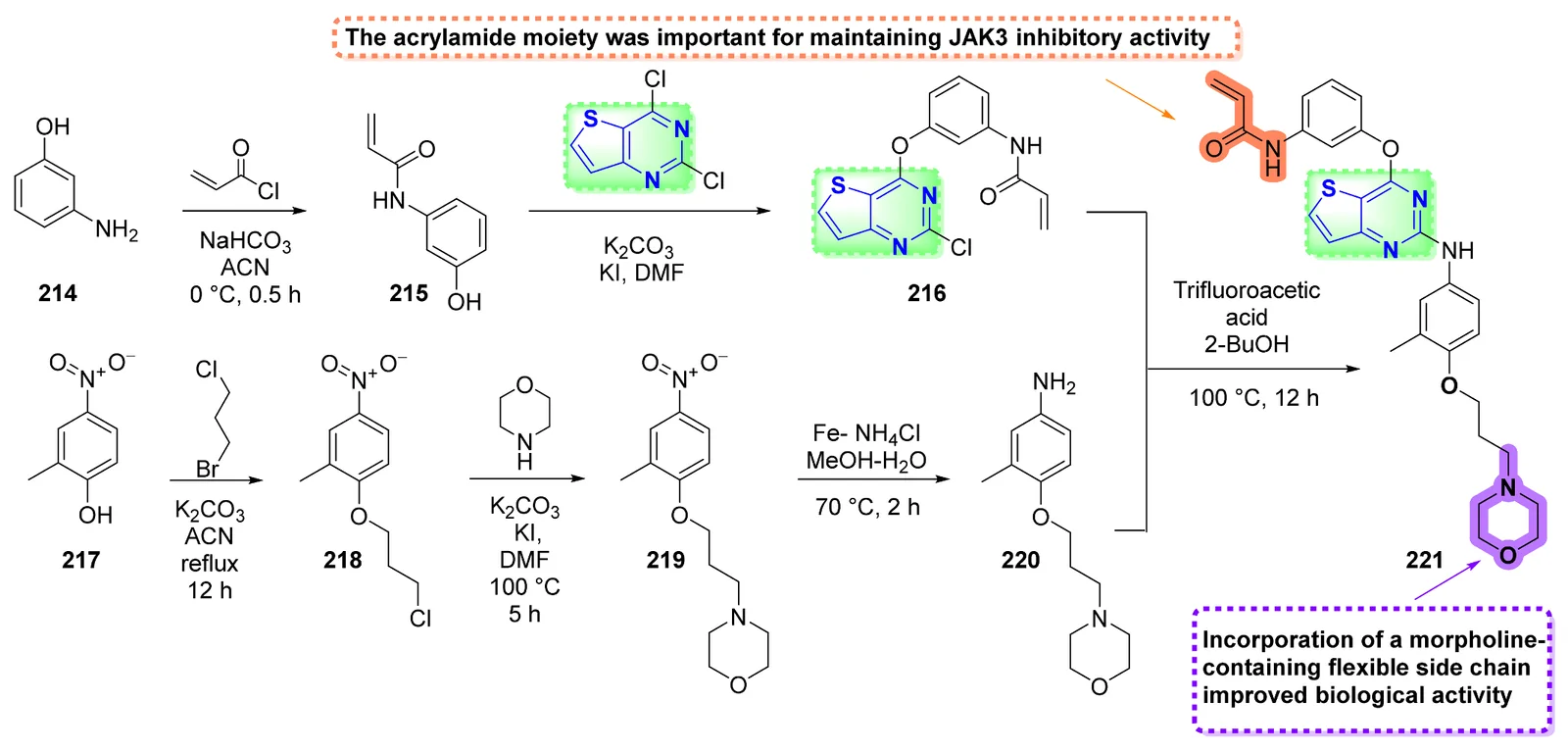
Structure and synthetic route of lead compound **221**.

Chi et al. [181] synthesized compound **221** as a selective JAK3 inhibitor targeting B-cell lymphomas. The synthesis involved acylation of 3-aminophenol, substitution on the thieno[3,2-*d*]pyrimidine ring, and final coupling under TFA to yield **221** (Scheme 48). The compound showed potent JAK3 inhibition (IC_50_ = 1.9 nM) and selective antiproliferative activity against B-cell lines Ramos, Raji, and Namalwa (IC_50_ ≈ 9–10 µM). SAR analysis showed that the morpholine-containing flexible side chain is crucial for biological activity, likely by increasing molecular adaptability within the JAK3 binding region. The acrylamide linker is also essential for maintaining strong JAK3 inhibition and selective antiproliferative effects against B-cell lymphoma cells. Viability assays confirmed its cytotoxic effect on malignant B-lymphocytes, underscoring its potential in hematologic cancers.

### 3.11. Dual/Multi-Target Anticancer Activity of Thieno[3,2-d]pyrimidines

#### 3.11.1. Dual mTOR/PI3K-Directed Anticancer Activity

**Scheme 49:**
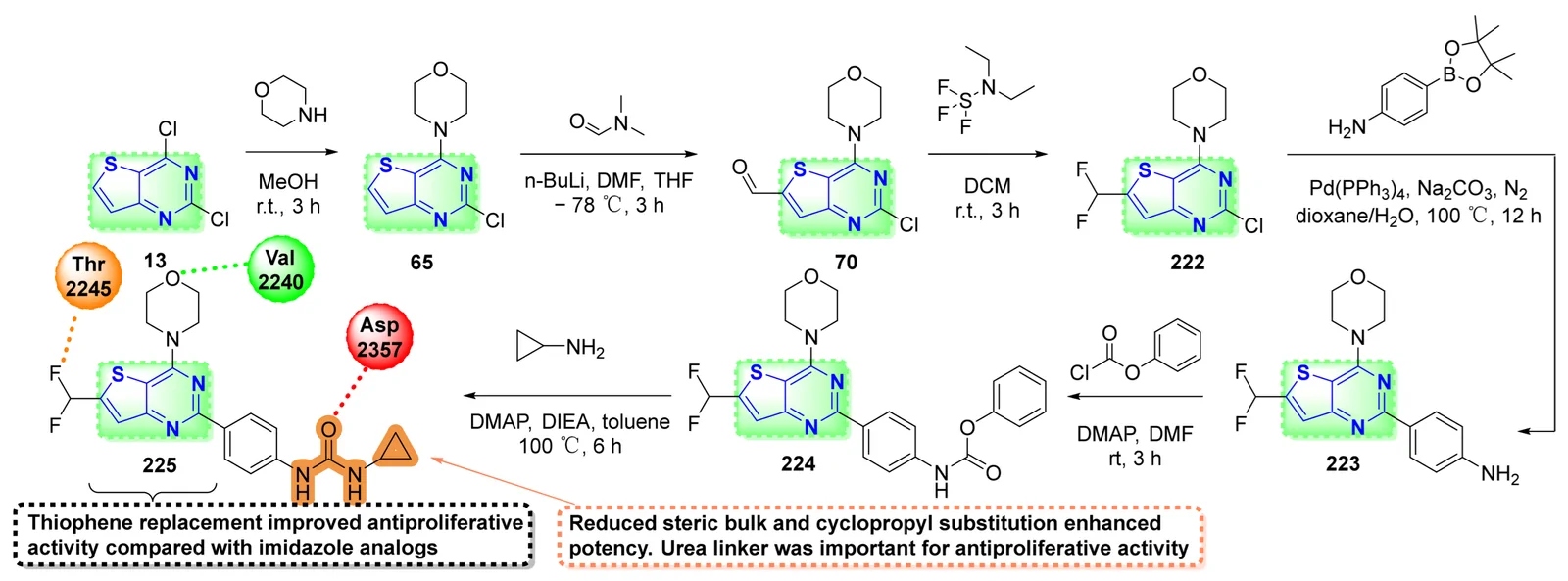
Structure and synthetic route of lead compound **225**.

Dual and multi-target anticancer agents work by affecting several cancer-related pathways at once [182,183,184,185,186]. This approach can make treatment more effective and help prevent resistance that often develops with drugs targeting only one pathway [187]. Yang et al. [188] developed thieno[3,2-*d*]pyrimidine derivatives targeting mTOR, identifying compound **225** as the lead. Its synthesis involved morpholine substitution at C-4, followed by formylation, fluorination, Suzuki coupling, and aminolysis (Scheme 49). Compound **225** inhibited mTOR with IC_50_ = 1.7 nM (99% at 100 nM), outperforming GDC-0349 and showing 142-fold selectivity over PI3Kα. It also showed superior antiproliferative activity in MCF-7, PC-3, A549, and MDA-MB-231 (IC_50_ = 0.08, 0.38, 0.47, and 0.47 µM) cells. In other breast cancer lines (MDAMB-468, MDAMB-435), its potency was comparable to GDC-0349. Mechanistically, **225** induced G0/G1 arrest, suppressed migration, and reduced AKT and P70S6K phosphorylation in a dose-dependent manner. Replacing the imidazole ring with thiophene significantly increased antiproliferative activity. The urea linker was crucial for potency, likely due to favorable hydrogen bonding. Reducing steric bulk further improved activity, with a cyclopropyl group yielding the best profile.

**Scheme 50:**
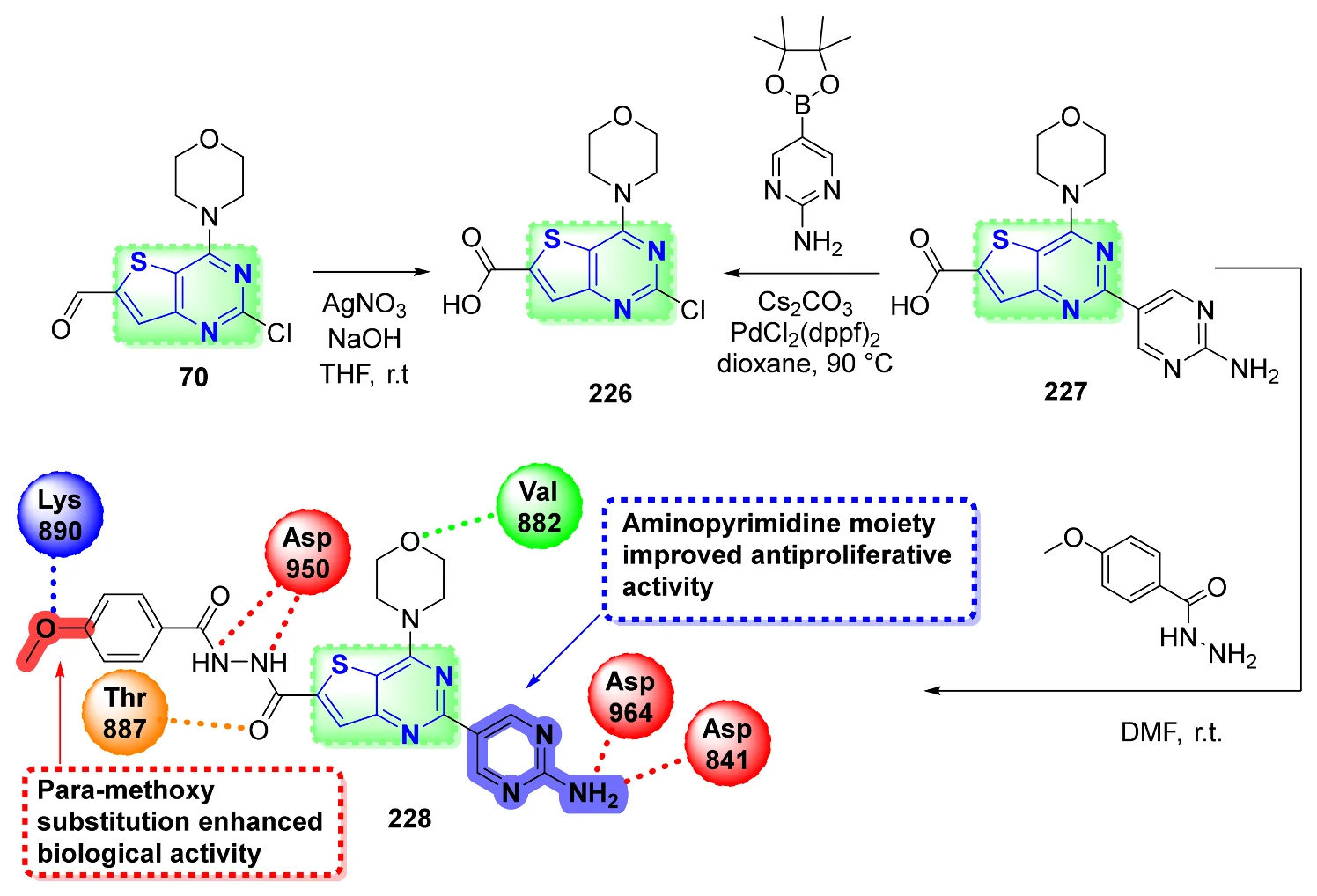
Structure and synthetic route of lead compound **228**.

Han et al. [189] synthesized compound **228** by replacing indazole with 2-aminopyrimidine, which improved its anti-proliferative activity against various cancer cell lines, including PC-3, HCT-116, A549, and MDA-MB-231 (IC_50_ = 1.55–2.02 μM), while maintaining its inhibitory activity against PI3Kα. SAR analysis showed that adding a 2-aminopyrimidine group greatly improved antiproliferative activity and kept strong PI3K/mTOR inhibition. The arylhydrazide fragment at the C-6 position was also found to be important for keeping activity. Adding a para-methoxy group to the phenyl ring further increased biological activity, likely due to favorable electronic and hydrophobic effects. In vitro assays showed potent inhibition of PI3K (IC_50_ = 0.46–55 nM) isoforms and mTOR (IC_50_ = 12 nM). The synthetic preparation involved several steps, starting with the reaction of compound **11** with urea, followed by Suzuki coupling and acylation to yield the final product (Scheme 50).

**Scheme 51:**
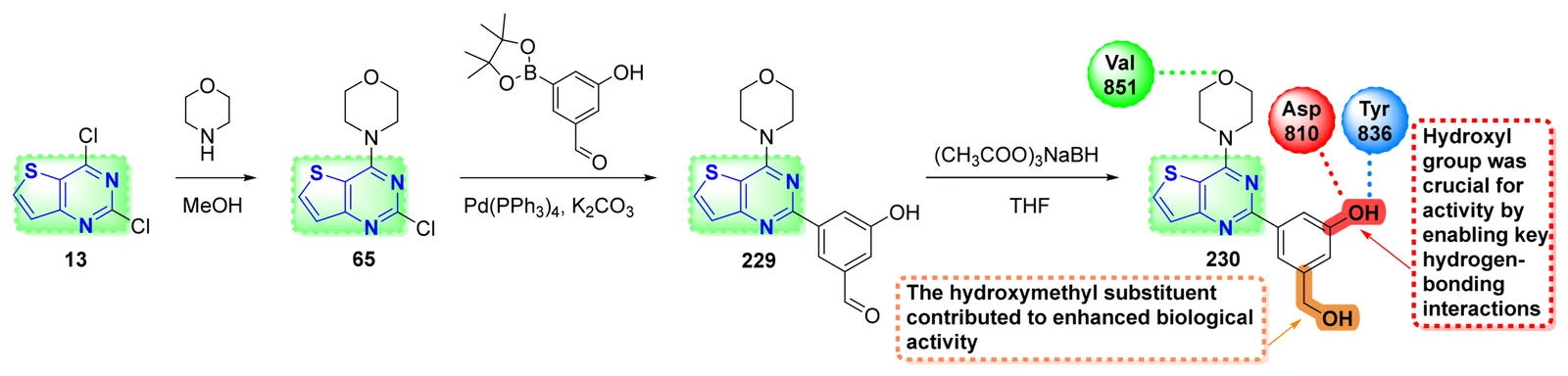
Structure and synthetic route of lead compound **230**.

A novel series of thieno[3,2-*d*]pyrimidine derivatives were prepared through Suzuki coupling of the morpholine-substituted intermediate, followed by reduction to yield compound **230** (Scheme 51) [190]. This compound showed potent inhibition of PI3Kα and mTOR (IC_50_ = 12.5 and 12.5 nM) and moderate activity against PI3Kβ, δ, γ (IC_50_ = 47.2–276 nM). Compound **230** inhibited the growth of PC-3, Rh30, and SKOV-3 cells more effectively than PI103, with IC_50_ values ranging from 0.15 to 0.26 µM. SAR analysis indicated that the hydroxymethyl substituent contributed to enhanced biological activity, likely through favorable hydrogen-bonding interactions within the binding site. In addition, small substituents with low steric demand were better tolerated and resulted in improved PI3Kα/mTOR inhibitory potency. Its activity profile suggests selective targeting of PI3Kα/δ in tumor cells.

**Scheme 52:**
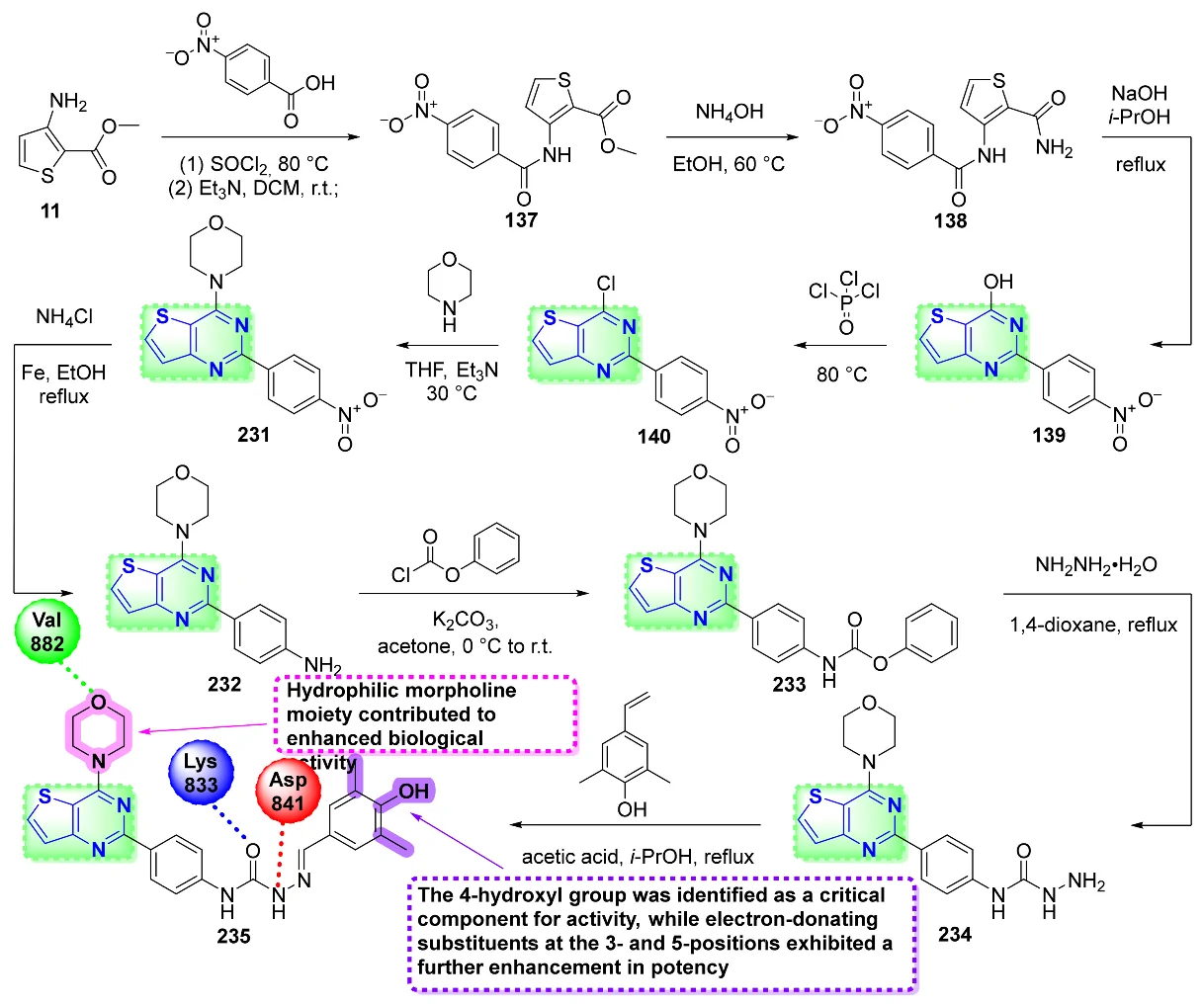
Structure and synthetic route of lead compound **235**.

Liu et al. [191] have synthesized a novel series of diaryl semicarbazone derivatives based on the thieno[3,2-*d*]pyrimidines. The synthesis began with the condensation of 4-nitrobenzoic acid and 3-amino-2-thiophenecarboxylate, followed by hydroxylamination and cyclization, affording compound **139**. Subsequent steps included chlorination, morpholine substitution, nitro reduction, and coupling with phenyl carbonochloridate to give intermediate **233**, which was converted into target compound **235** via hydrazinolysis and condensation with 2,6-dimethyl-4-vinylphenol (Scheme 52). Compound **235** showed potent cytotoxicity against H460, HT-29, MKN-45, and MDA-MB-231 cells (IC_50_ = 0.039–0.25 µM). It also selectively inhibited PI3Kα (IC_50_ = 0.027 µM) and mTOR (IC_50_ = 0.77 µM), and induced apoptosis in HT-29 cells, highlighting its relevance for further study in lung and colorectal cancers. SAR analysis showed that the hydrophilic morpholine group improved biological activity, possibly by facilitating the compound’s interaction with the binding site. The hydroxyl group at the 4-position was essential for strong activity, and the introduction of electron-donating groups at the 3- and 5-positions further increased antiproliferative potency.

**Scheme 53:**
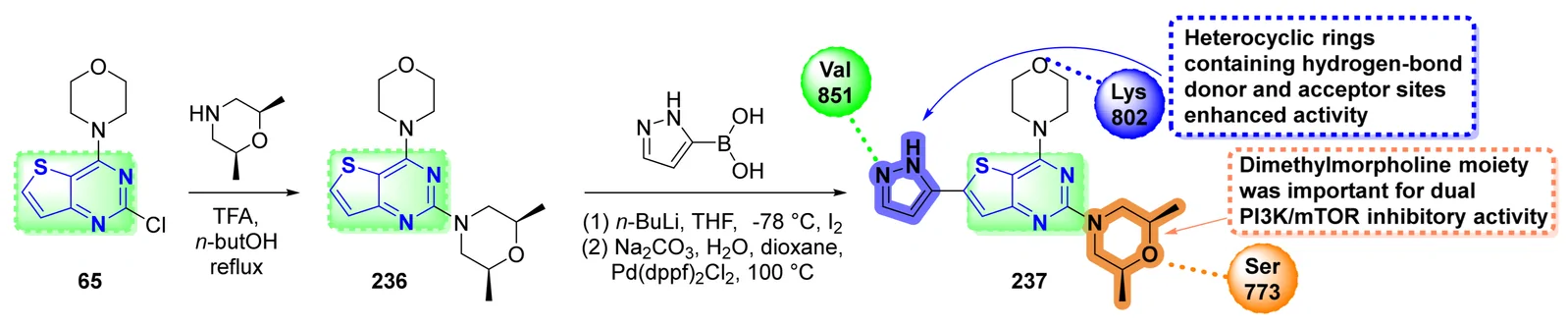
Structure and synthetic route of lead compound **237**.

A dual PI3K/mTOR inhibitor **237** through structural optimization of the known agent pictilisib was presented by Zhan and co-workers [192]. The target compound was synthesized by introducing a (2R,6S)-2,6-dimethylmorpholine moiety at C-2 and a pyrazole group at C-6 of the thienopyrimidine core via Suzuki coupling (Scheme 53). The hybrid molecule **237** demonstrated markedly improved inhibition of PI3Kα (IC_50_ = 15 nM) and mTOR (IC_50_ = 16 nM) compared to pictilisib. **237** inhibited PI3Kβ, PI3Kγ, and PI3Kδ with IC_50_ values of 175, 75, and 10 nM, indicating moderate activity compared to pictilisib. In addition, it exhibited strong antiproliferative effects in various cancer cell lines, such as PC-3 and U87MG, with IC_50_ values below 0.5 µM. In xenograft models, **237** demonstrated robust tumor growth inhibition with minimal toxicity, highlighting its promise as a lead candidate for further development. SAR analysis showed that incorporation of the dimethylmorpholine moiety significantly improved dual PI3K/mTOR inhibitory activity. In addition, heterocyclic fragments containing hydrogen-bond donor and acceptor functionalities further enhanced potency through favorable interactions within the kinase binding pocket.

**Scheme 54:**
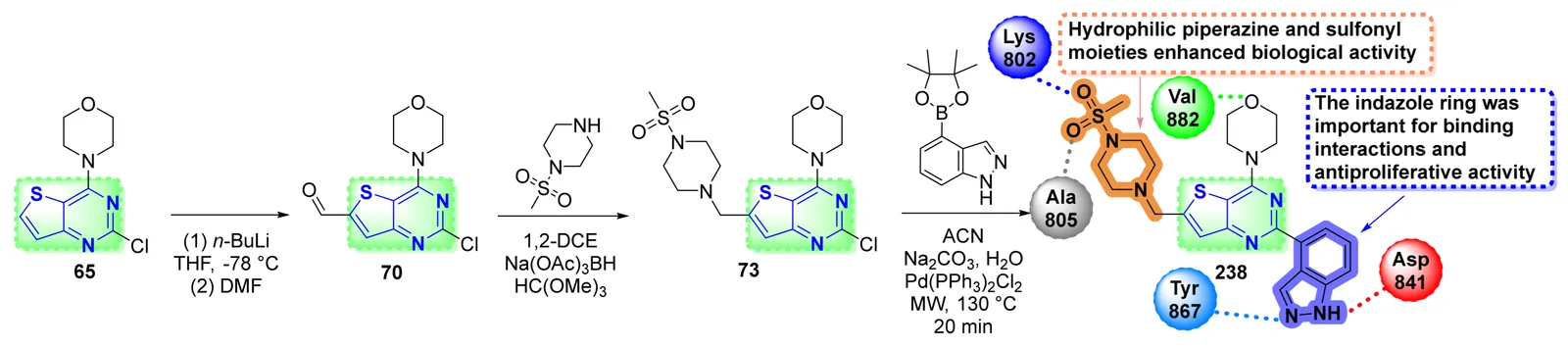
Structure and synthetic route of lead compound **238**.

Folkes et al. [193] designed and synthesized thienopyrimidine derivatives as selective PI3K inhibitors, with compound **238** (Pictilisib™) emerging as a potent lead. Its synthesis involved lithiation and aldehydation at C-6 of the thienopyrimidine core, followed by piperazine coupling and Suzuki reaction (Scheme 54). Compound **238** showed exceptional activity against p110α (IC_50_ = 0.003 μM) and maintained submicromolar IC_50_ values in U87MG and A2780 (IC_50_ = 0.95 and 0.14 µM) cells. It also inhibited multiple PI3K isoforms and mTOR, with broad antiproliferative effects in U87MG, PC3, and MDA-MB-361 (IC_50_ = 0.95, 0.28, and 0.72 μM), and reduced pAkt473 levels in the same lines (IC_50_ = 46, 37, and 28 nM, respectively). In vivo assays demonstrated 83% tumor inhibition in U87MG xenografts without body weight loss, supporting its development as an orally active anticancer agent. SAR analysis showed that adding hydrophilic piperazine and sulfonyl groups enhanced antiproliferative activity by improving polarity and target interactions. The indazole moiety also sustained potent activity through favorable kinase binding.

**Scheme 55:**
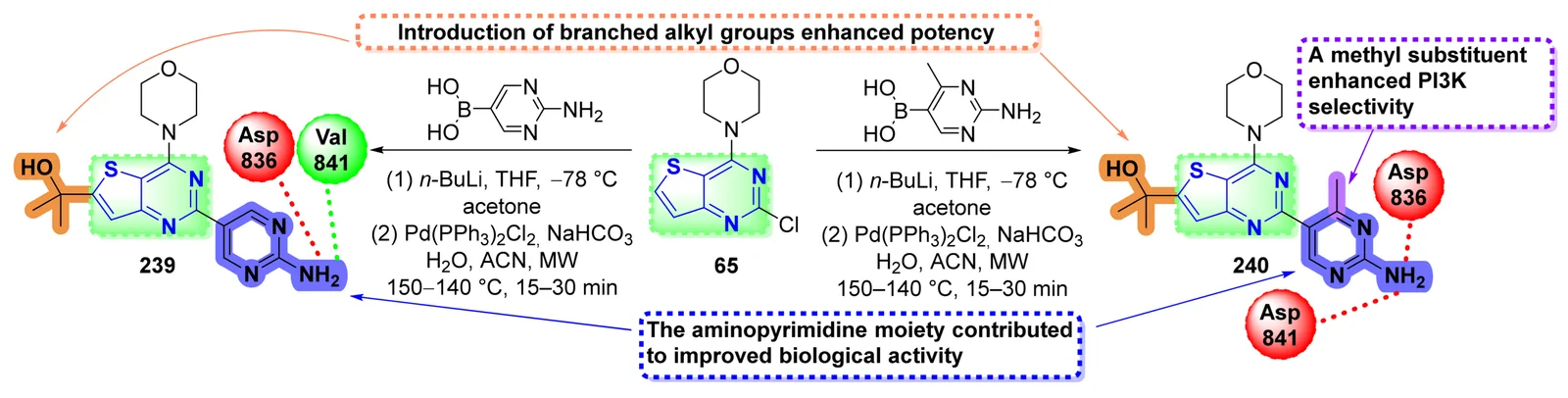
Structure and synthetic route of lead compounds **239** and **240**.

A series of thienopyrimidines bearing morpholine and pyrimidine moieties was designed by Sutherlin and co-workers [194] as PI3K and dual PI3K/mTOR inhibitors. The target compounds, **239** and **240**, were synthesized via Suzuki coupling using the corresponding boronic acids (Scheme 55). Compound **239** exhibited high selectivity for Aurora A, MLK1, and SYK kinases, with IC_50_ values of greater than 10 μM, 591 nM, and 371 nM, respectively. It also exhibited 65% and 78% inhibition of PI3KC2α and PI3KC2β at 1 μM, while compound **240** inhibited PI3KC2β by 81%. In in vivo xenograft models, compound **239** showed 2.5-fold greater exposure than **240** at 5 mg/kg and demonstrated superior efficacy against PC3 and MCF7.1 cell lines. Both compounds displayed potent enzymatic activity against PI3Kα/β/δ/γ/mTOR (IC_50_ (**239**) = 3.4/12/16/16/32 nM and IC_50_ (**240**) = 3.5/25/5.2/15/740 nM), and **239** (GNE-493) and **240** (GNE-490). SAR analysis showed that adding branched alkyl groups increased inhibitory potency by strengthening hydrophobic interactions in the binding pocket. The aminopyrimidine moiety was essential for biological activity, and the addition of a methyl group improved PI3K selectivity.

**Scheme 56:**
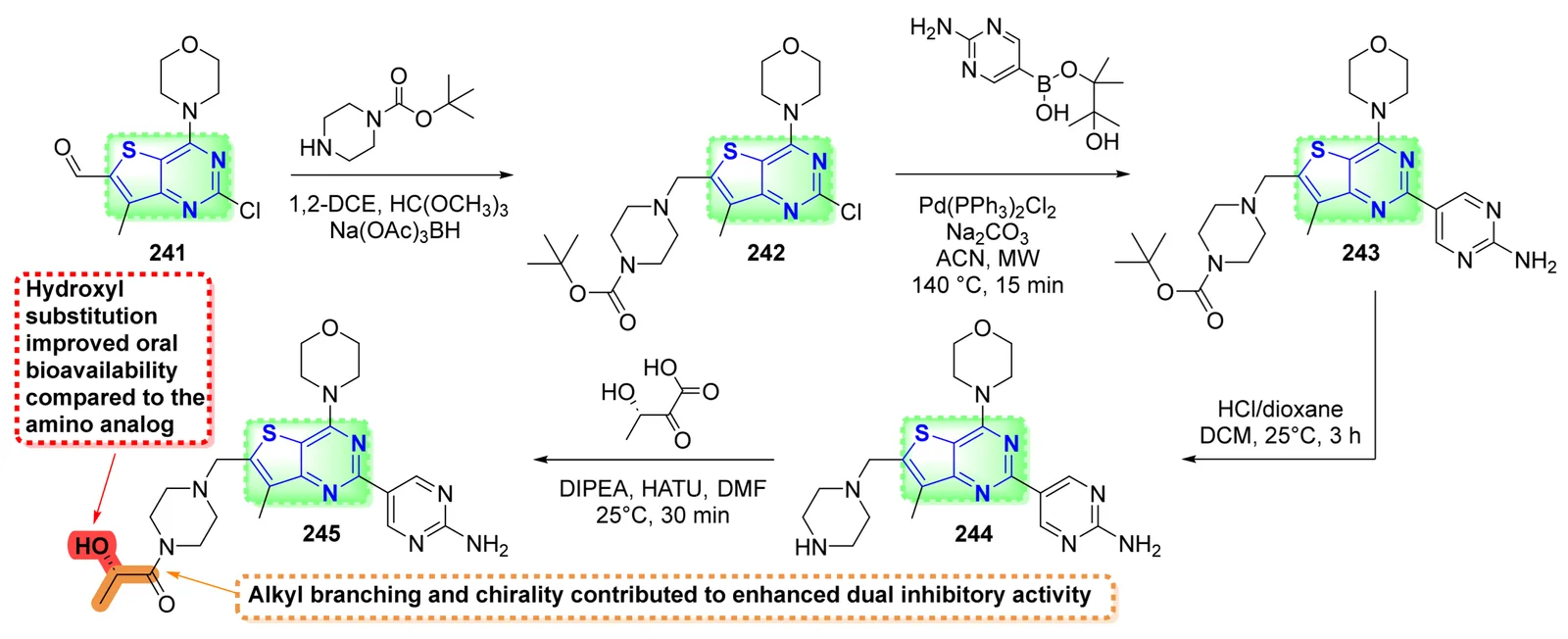
Structure and synthetic route of lead compound **245**.

The same group [195] reported the development of **238** (Pictilisib, GDC-0980) analog based on a thienopyrimidine scaffold fused to tert-butylpiperazine and further modified via Suzuki coupling to afford compound **245** (Scheme 56). This lead molecule demonstrated potent inhibition of PI3K p110α/β/δ/γ isoforms (IC_50_ = 5/27/7/14 nM) and mTOR (K_i_ = 17 nM), while maintaining selectivity across the PIKK family. Compound **245** also inhibited C2α, C2β, VPS34, and DNA-PK with moderate selectivity. Pharmacokinetic studies in mice (oral F = 56–66%) and protein binding (PPB = 71%) supported favorable bioavailability. In in vivo xenograft assays, compound **245** induced tumor stasis or regression in PC-3 and MCF-7 neo/HER2 models at 7.5 mg/kg, confirming its therapeutic potential as a dual PI3K/mTOR inhibitor (GDC-0980). SAR analysis indicated that replacing the amino group with a hydroxyl group improved oral bioavailability. In addition, alkyl branching and molecular chirality positively influenced dual PI3K/mTOR inhibitory activity, suggesting the importance of stereochemical and hydrophobic effects for optimal target binding.

#### 3.11.2. EGFR/ErbB-2-Directed Anticancer Activity

**Scheme 57:**
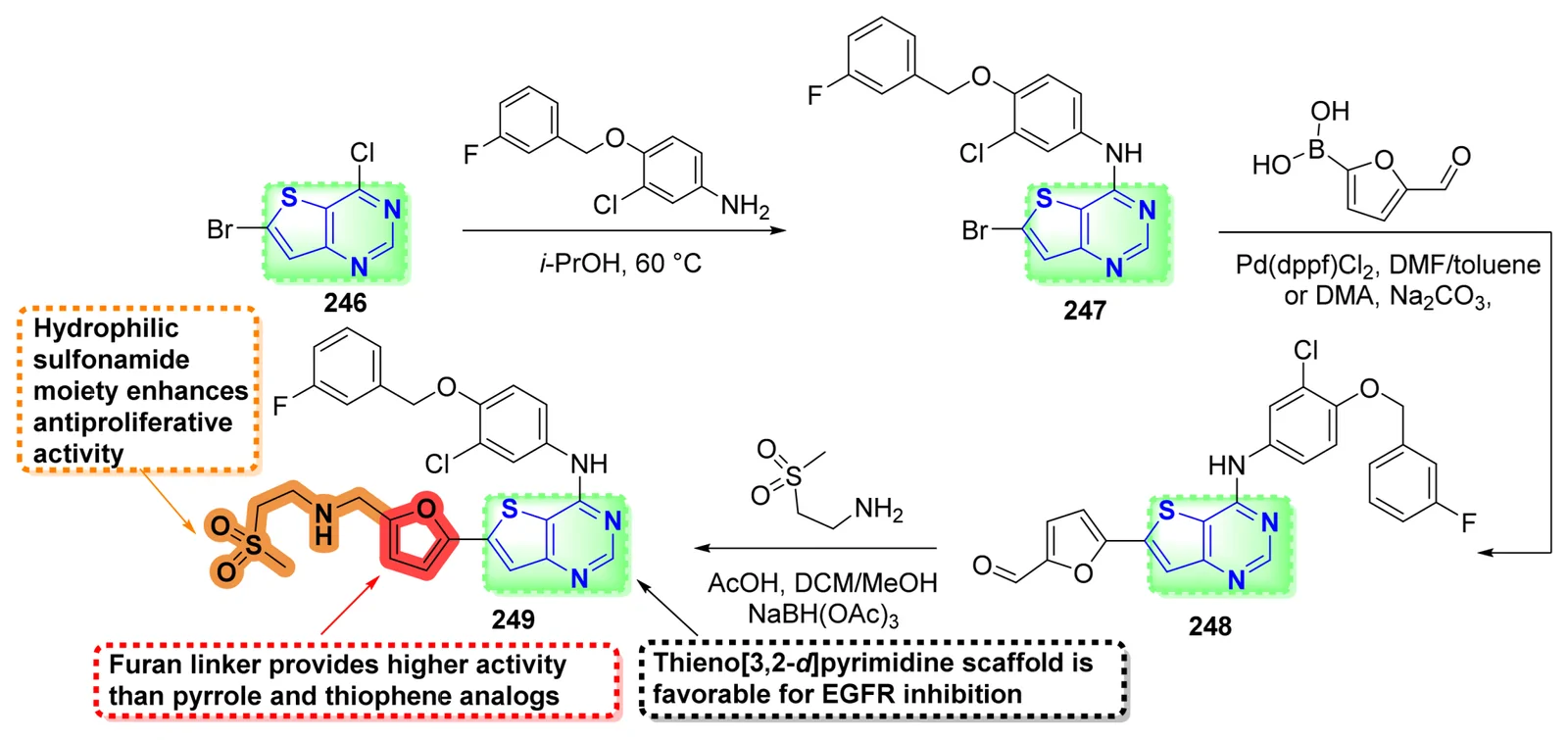
Structure and synthetic route of lead compound **249**.

EGFR/ErbB-2-targeted anticancer compounds have demonstrated promising therapeutic potential by simultaneously inhibiting two important signaling pathways involved in tumor growth, proliferation, and cancer cell survival [196,197]. Rheault et al. [198] developed a series of thienopyrimidine analogs featuring furan and thiophene linkers to target the EGFR and ErbB-2 receptors. The synthesis of lead compound **249** involved C-4 substitution of 6-bromo-4-chlorothieno[3,2-*d*]pyrimidine with an aniline derivative, followed by Suzuki coupling at C-6 and addition of a sulfonylamine moiety (Scheme 57). Hybrid derivative **249** inhibited EGFR-driven proliferation 20-, 2-, and 11-fold more potently than gefitinib, erlotinib, and lapatinib (IC_50_ = 20, 2, and 10.8 nM), respectively, having an IC_50_ of 1 nM. It also showed moderate activity against ErbB-2 (IC_50_ = 71 nM). SAR analysis showed that adding a hydrophilic sulfonamide improved antiproliferative activity by enhancing polarity and target interactions. Furan linkers outperformed pyrrole and thiophene. The thieno[3,2-*d*]pyrimidine scaffold was most favorable for EGFR inhibition. These results suggest that furan-linked thienopyrimidines could be helpful as dual EGFR/ErbB-2 inhibitors.

**Scheme 58:**
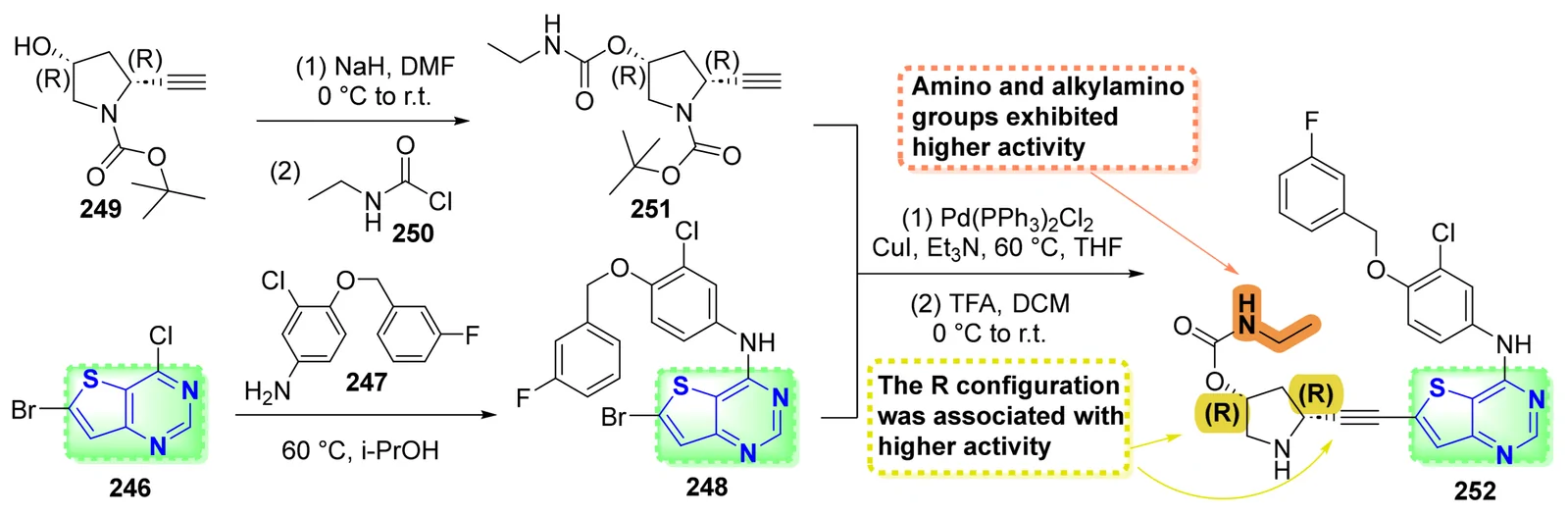
Structure and synthetic route of lead compound **252**.

Stevens and colleagues [199] introduced a new series of thienopyrimidines featuring a pyrrolidine group, aimed at dual inhibition of EGFR and ErbB-2 kinases. The lead compound, **252**, was synthesized by coupling intermediate **248**, formed by reacting thienopyrimidine **246** with a substituted aniline, with a functionalized pyrrolidine derivative (Scheme 58). Among the tested derivatives, thienopyrimidine hybrid **252** demonstrated the most potent activity. It inhibited EGFR and ErbB-2 with IC_50_ values of 32 nM and 20 nM, respectively. It significantly reduced the proliferation of BT474 and ErbB-2 Delfia cancer cell lines, with IC_50_ values of 20 nM and 10 nM. SAR studies revealed that amino and alkylamino substituents were more favorable for antiproliferative activity than other analogs. Stereochemistry played an important role, with compounds bearing the R configuration showing significantly enhanced biological activity. Additionally, pharmacokinetic studies in mice demonstrated good oral absorption. These findings underscore compound **252** as a promising lead for the development of dual-targeted anticancer therapies.

#### 3.11.3. Dual Tubulin/EGFR-Directed Anticancer Activity

**Scheme 59:**
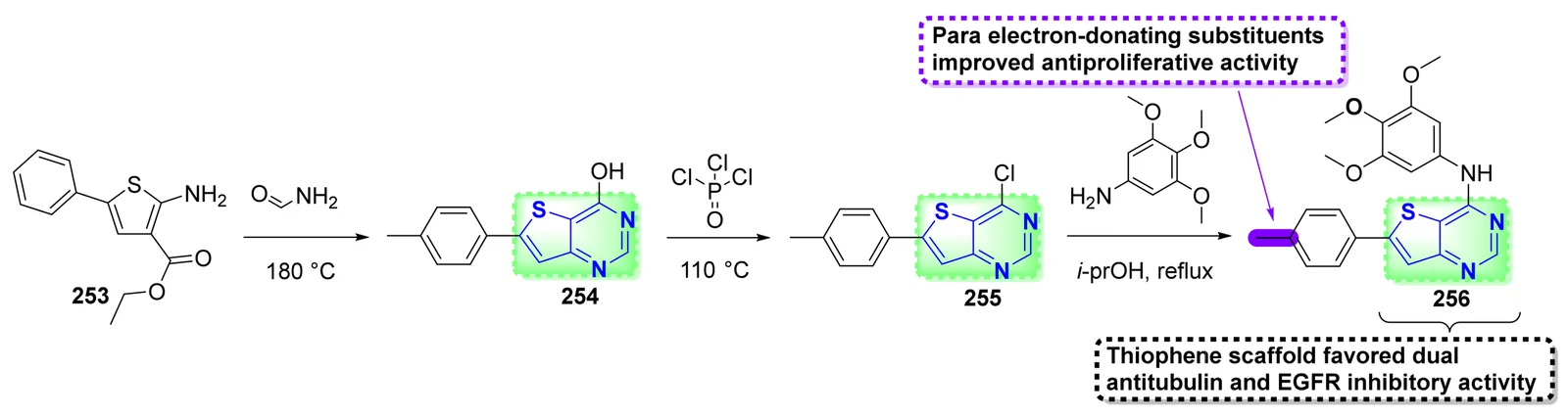
Structure and synthetic route of lead compound **256**.

Dual tubulin/EGFR-targeted compounds have shown strong potential in cancer therapy because they can block EGFR signaling while disrupting tubulin polymerization, thereby improving anticancer activity and helping overcome drug resistance [200,201]. Romagnoli and colleagues [202] developed a series of benzene-fused pyrimidines with multitarget anticancer potential. The synthesis of the lead compound **256** began from ethyl 2-amino-5-phenylthiophene-3-carboxylate **253**, which underwent cyclization with formamide, halogenation via POCl_3_, and subsequent coupling with 3,4,5-trimethoxyaniline (Scheme 59). Compound **256** exhibited superior antiproliferative activity against A549 (IC_50_ = 0.019 μM), HeLa (IC_50_ = 0.001 μM), HT-29 (IC_50_ = 0.02 μM), and Jurkat (IC_50_ = 0.001 μM) cell lines, outperforming CA-4 in all cases. It also showed potent antitubulin activity (76% inhibition, IC_50_ = 0.71 μM), surpassing CA-4 (IC_50_ = 1.2 μM). Compound **256** showed potent EGFR inhibition with an IC_50_ of 30 nM, possibly due to the para-methyl group at position C-6. In vivo tests on B16 melanoma cells confirmed this activity, with EGFR kinase inhibition (IC_50_ = 23.4 ± 3.8 nM) and a 52.5% reduction in tumor growth at 7.5 mg/kg, which was more effective than CA-4P (34.9% at 30 mg/kg), without any signs of toxicity. SAR studies showed that electron-donating groups at the para-position of the aryl ring positively influenced antiproliferative activity. In addition, thiophene-containing analogs exhibited higher activity than the corresponding thiazole derivatives, indicating that the thiophene scaffold is favorable for dual antitubulin and EGFR inhibitory activity. These findings suggest that aryl-substituted thienopyrimidines, such as **256**, are promising candidates for anticancer drug development.

#### 3.11.4. Dual EGFR/PI3K-Directed Anticancer Activity

**Scheme 60:**
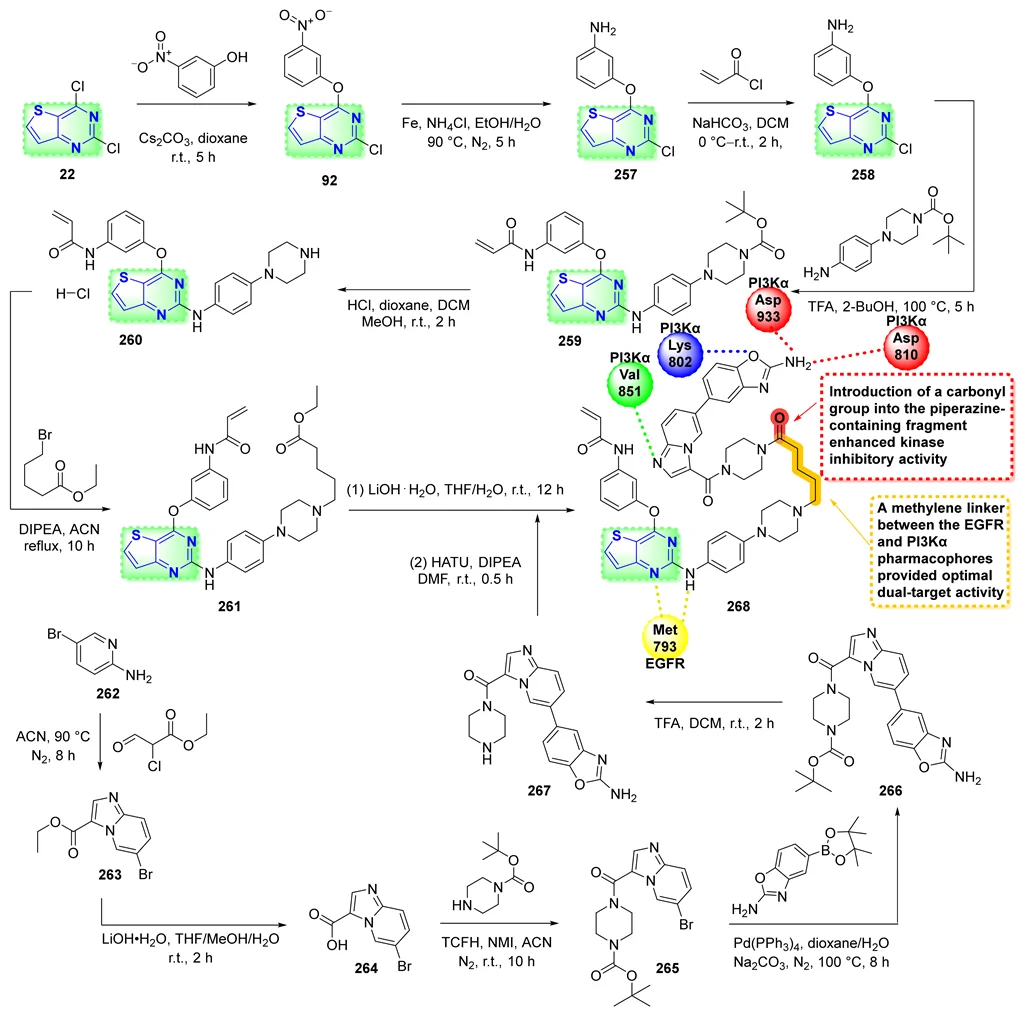
Structure and synthetic route of lead compound **268**.

Dual targeting of EGFR/PI3K has emerged as an effective anticancer strategy by simultaneously suppressing tumor growth, survival, and resistance-related signaling pathways [203]. Wang et al. [204] developed hybrid thienopyrimidine-based inhibitors inspired by Olmutinib and Cerabelisib, identifying **268** as the lead compound. Its synthesis involved two converging routes: the first included etherification, reduction, and amidation at C-4, followed by Buchwald–Hartwig coupling at C-2 to yield intermediate **261** (Scheme 60).

The second pathway used intercyclization, Suzuki, and Buchwald–Hartwig couplings to construct the complementary fragment. Final coupling afforded the target compound **268**. It exhibited strong inhibition of PI3Kα (IC_50_ = 30 nM) and EGFR^L858R/T790M^ (IC_50_ = 3.6 nM), outperforming TAK-117 and Olmutinib by over 100-fold. Antiproliferative assays in A549 and NCI-H1975 cells confirmed superior efficacy and 20-fold selectivity for mutant EGFR. SAR studies revealed that incorporation of a carbonyl group into the piperazine-containing fragment significantly improved kinase inhibitory potency. In addition, the linker length played a critical role in dual-target activity, with a methylene spacer between the EGFR and PI3Kα pharmacophores providing the most favorable inhibitory profile. These findings support **268** as a potent dual-target inhibitor.

#### 3.11.5. ATR/mTOR-Directed Anticancer Activity

**Scheme 61:**
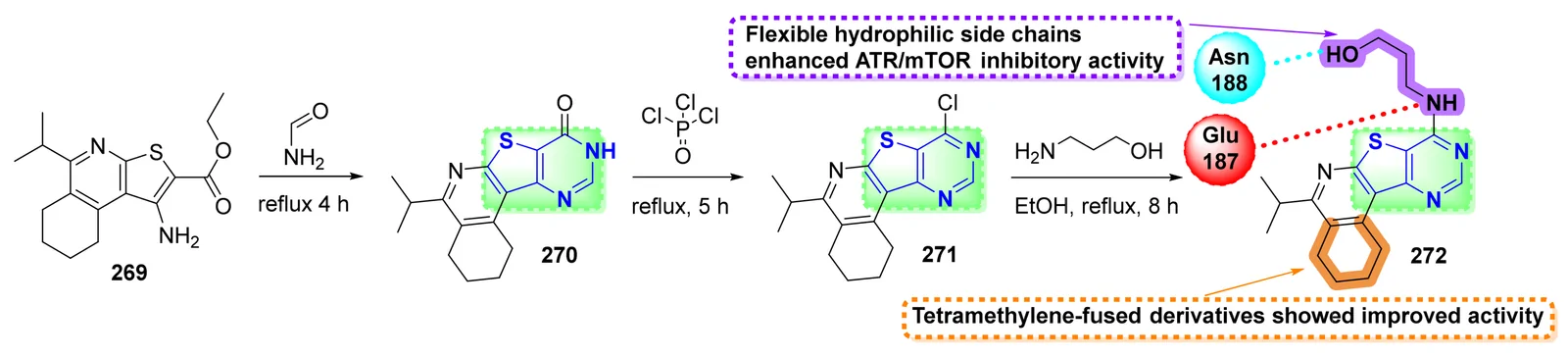
Structure and synthetic route of lead compound **272**.

Sirakanyan et al. [56] designed a series of tetracyclic thienopyrimidines as dual ATR/PIKK inhibitors through intercyclization, halogenation, and amination steps (Scheme 61). Among them, compound **272** displayed the most vigorous activity, with IC_50_ values of 7.16 μM (HCT116) and 4.80 μM (HeLa). Docking showed that **272** interacts with ATR via π–π stacking and hydrogen bonds involving Trp87. The thienopyrimidine core played a key role in target binding, as confirmed by SAR and hydrophobic interactions. Also, SAR studies showed that flexible hydrophilic side chains enhanced ATR/mTOR inhibitory activity, likely by promoting more favorable interactions with the target proteins. Moreover, tetramethylene-fused derivatives exhibited improved biological activity compared with related analogs, indicating a beneficial effect of the fused ring system. Compound **272** also showed signs of influencing mTOR and ATM, making it a promising anticancer scaffold.

#### 3.11.6. BET/PI3K-Directed Anticancer Activity

**Scheme 62:**
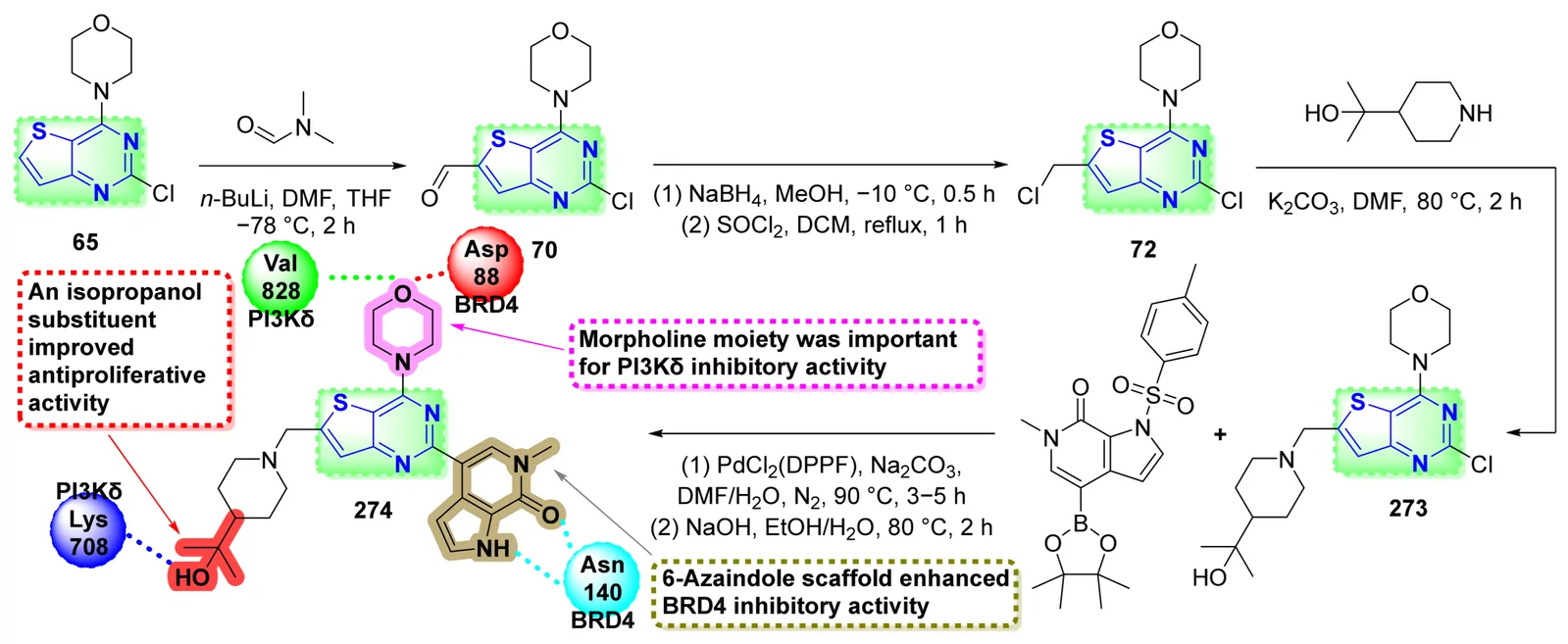
Structure and synthetic route of lead compound **274**.

Ran et al. [205] synthesized bifunctional PI3Kδ–BET inhibitors, identifying compound **274** as the lead. Its synthesis involved formylation, chlorination, *C*–*N* coupling, and Suzuki–Miyaura coupling of compound **65** (Scheme 62); **274** showed moderate inhibition of PI3Kδ (41.6%) at 100 nM and BRD4-BD1 (33.7%) at 10 nM. With IC_50_ values of 112 nM (PI3Kδ), 19 nM (BRD4-BD1), and antiproliferative activity against SU-DHL-4 and SU-DHL-6 cells, it exhibited strong selectivity for BET (9.5–117.5-fold over SF2523). In vivo, **274** showed 74.8% oral bioavailability and 70.8% TGI in the SU-DHL-6 xenograft model, positioning it as a promising dual inhibitor for DLBCL. SAR studies revealed that incorporation of an isopropanol-containing substituent improved antiproliferative activity. The morpholine fragment was important for maintaining potent PI3Kδ inhibition, whereas the 6-azaindole scaffold contributed to enhanced BRD4 inhibitory activity, supporting the dual-target profile of compound **274**.

#### 3.11.7. PARP/PI3K-Directed Anticancer Activity

**Scheme 63:**
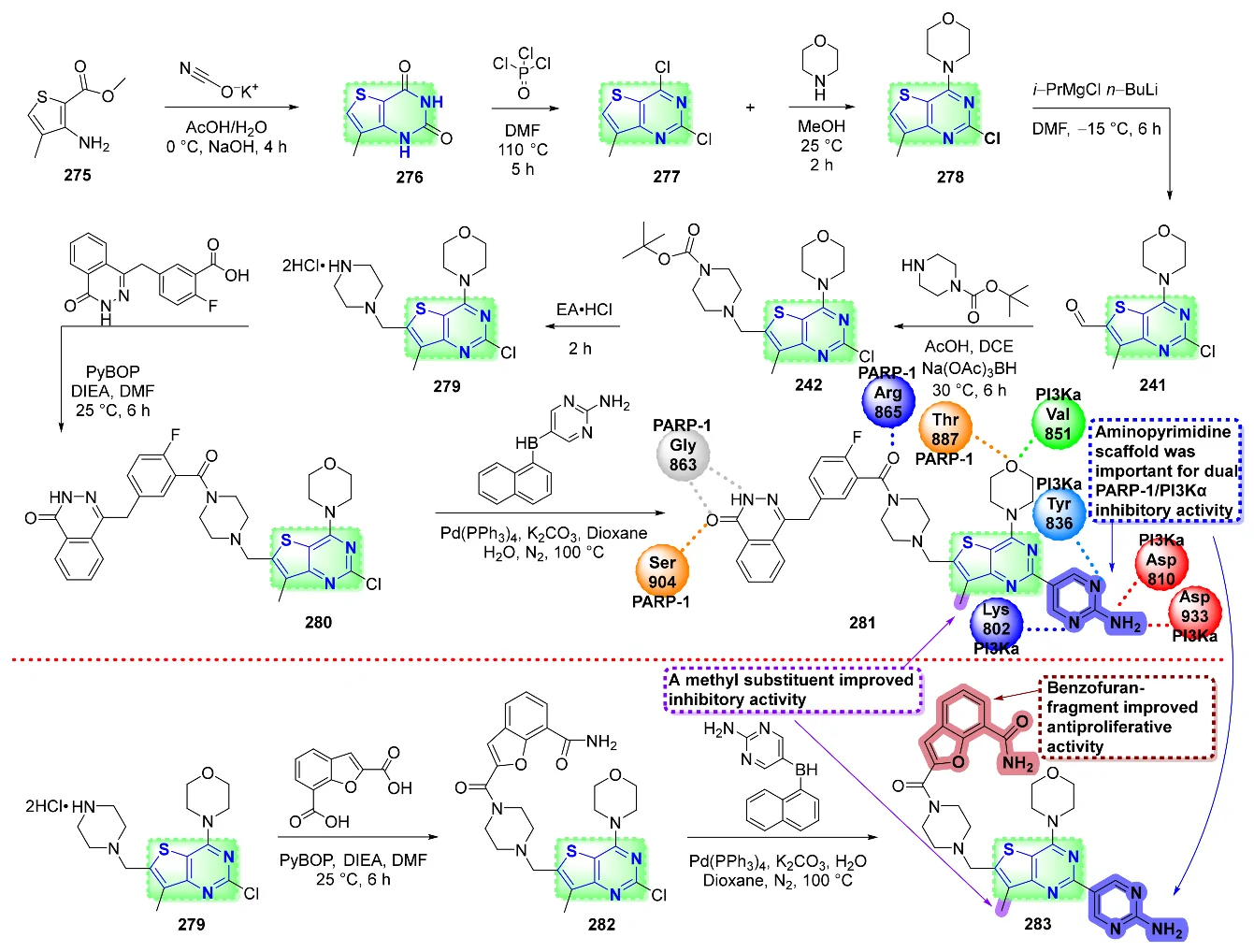
Structure and synthetic routes of lead compounds **281** and **283**.

Wu et al. [206] developed thienopyrimidine-based dual inhibitors of PARP and PI3K. They found that compounds **281** and **283** effectively inhibited PARP-1 (IC_50_ = 1.57 and 0.91 nM) and PI3Kα (IC_50_ = 2.0 and 1.5 nM) in vitro. In mouse models, both compounds suppressed tumor growth more effectively than olaparib, with TGI values of 56.39% and 48.77%, respectively, versus 27.91% for olaparib. No adverse effects were observed in the mice. Compound **281** was synthesized from methyl-3-amino-4-methylthiophene-2-carboxylate, and compound **283** was derived from a derivative of benzoic acid via amidation followed by Suzuki coupling (Scheme 63). Both compounds show potential for cancer therapy. SAR analysis indicated that the aminopyrimidine scaffold played an important role in maintaining dual PARP-1/PI3Kα inhibitory activity. Introduction of a methyl substituent slightly improved inhibitory potency, whereas incorporation of the benzofuran moiety in compound **283** further enhanced both antiproliferative and enzymatic inhibitory activities compared with compound **281**.

#### 3.11.8. JAK/BTK-Directed Anticancer Activity

**Scheme 64:**
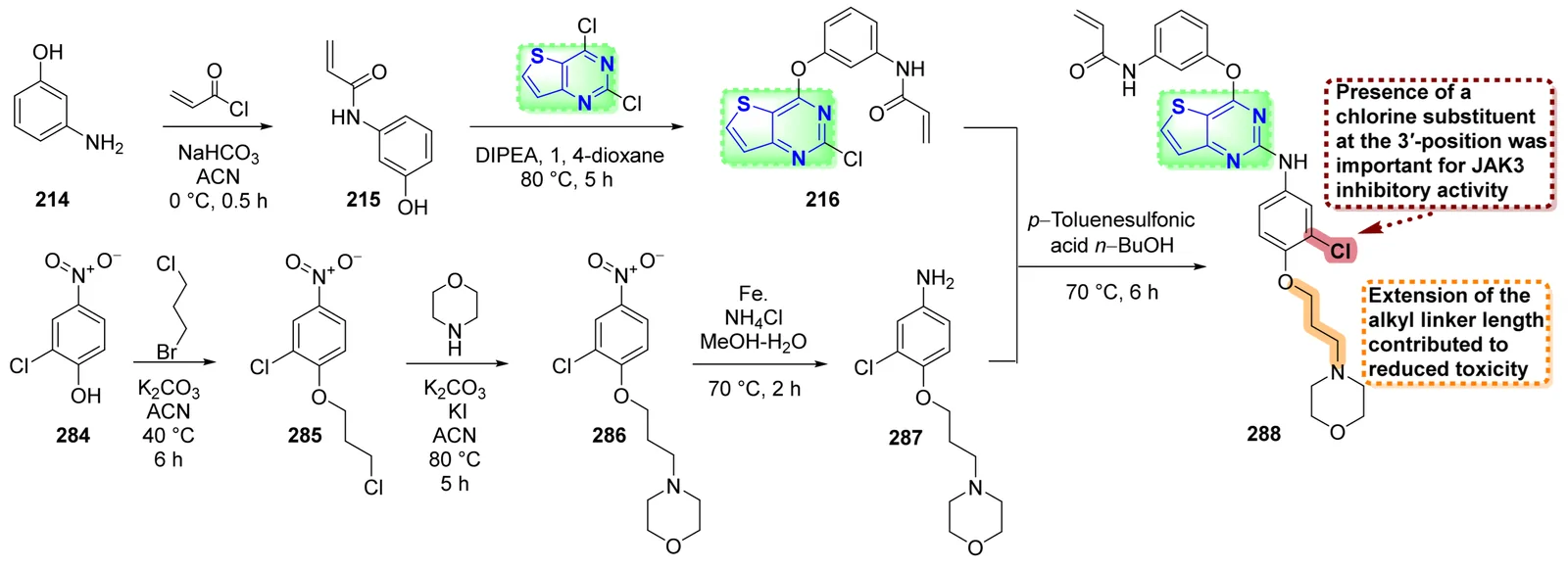
Structure and synthetic route of lead compound **288**.

Derivative **288** was synthesized via stepwise construction involving aryl acrylate formation, heterocyclic substitution on a dichloropyrimidine core, and final condensation of intermediates **216** and **287** (Scheme 64) [77]. The molecule exhibited potent inhibition of JAK3 (IC_50_ = 1.38 nM) and BTK (IC_50_ = 62.4 nM), kinases implicated in hematologic malignancies and immune-regulated tumors. Fibrosis was assessed in mice using a BLM-induced lung model. Compound **288** lowered collagen deposition compared to the control group. This effect complements its kinase inhibition profile. SAR analysis showed that adding a chlorine group at the meta-position of the aniline fragment increased JAK3 inhibitory activity. Extending the alkyl linker reduced toxicity and improved the overall biological profile, suggesting that optimizing the linker is important for balancing potency and tolerability.

#### 3.11.9. PI3K/PIM/mTOR-Directed Anticancer Activity

**Scheme 65:**
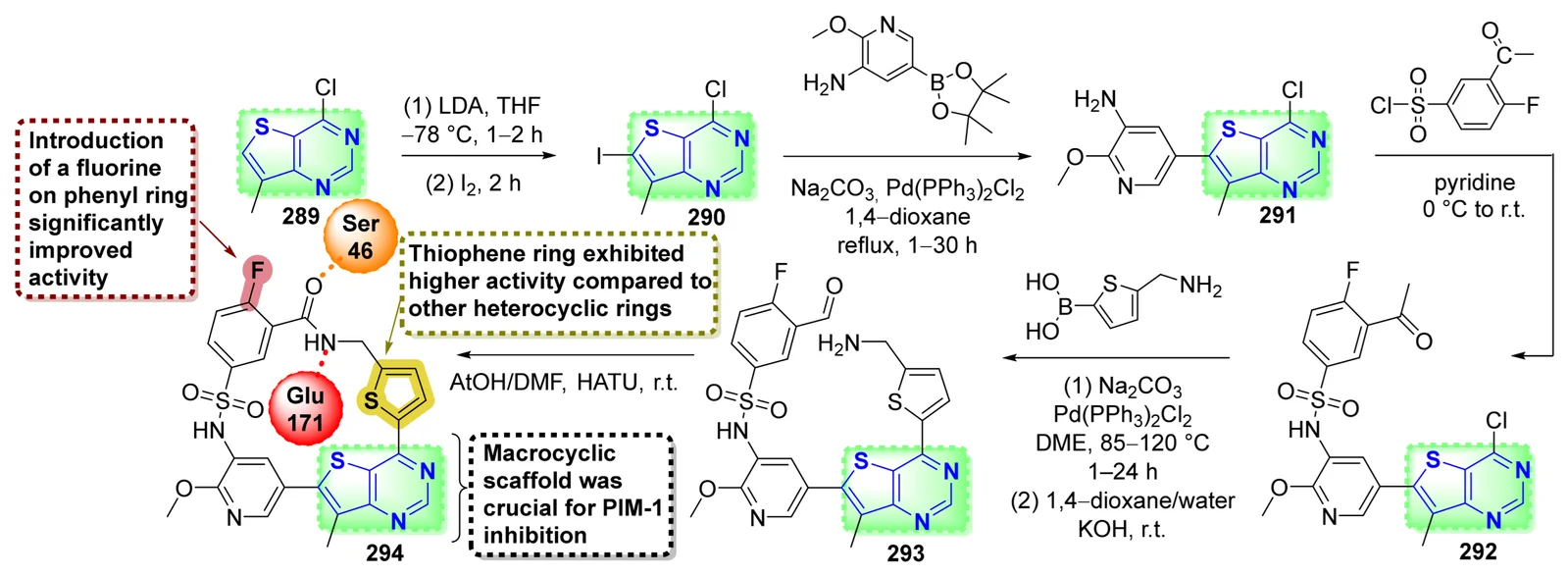
Structure and synthetic route of lead compound **294**.

Martínez-González et al. [207] designed and synthesized a macrocyclic thieno[3,2-*d*]pyrimidine scaffold, culminating in the identification of the lead compound **294**. The synthetic route involved initial halogenation of compound **289** at the C-6 position, followed by Suzuki couplings to introduce a 5-amino-6-methoxypyridine unit and a thiophenyl moiety. Subsequent macrocyclization yielded compound **294** bearing a lactam-bridged structure (Scheme 65). Anticancer activity revealed that compound **294** exhibited exceptional dual-target inhibitory activity, particularly against PI3Kα (IC_50_ = 0.13 nM) and PIM-1 (IC_50_ = 22.27 nM), outperforming the reference inhibitor omipalisib. Additionally, it showed potent antiproliferative activity against mTOR (IC_50_ = 74.53 nM) and maintained broad efficacy across related PI3K and PIM isoforms. Notably, **294** demonstrated excellent metabolic stability in human liver microsomes (>98%), suggesting favorable pharmacokinetic properties. Although its in vivo stability in rodent models was moderate (63–65%), the compound’s potency and selectivity highlight its promise as a lead structure for further development of anticancer drugs. SAR analysis indicated that incorporation of a fluorine substituent on the phenyl ring improved inhibitory activity, while the thiophene-containing analogs displayed superior potency compared with other heterocyclic derivatives. In addition, the macrocyclic scaffold appeared to play an important role in achieving potent PIM-1 inhibitory activity and maintaining the overall dual-target profile.

#### 3.11.10. Multi-Target Anticancer Activity

**Scheme 66:**
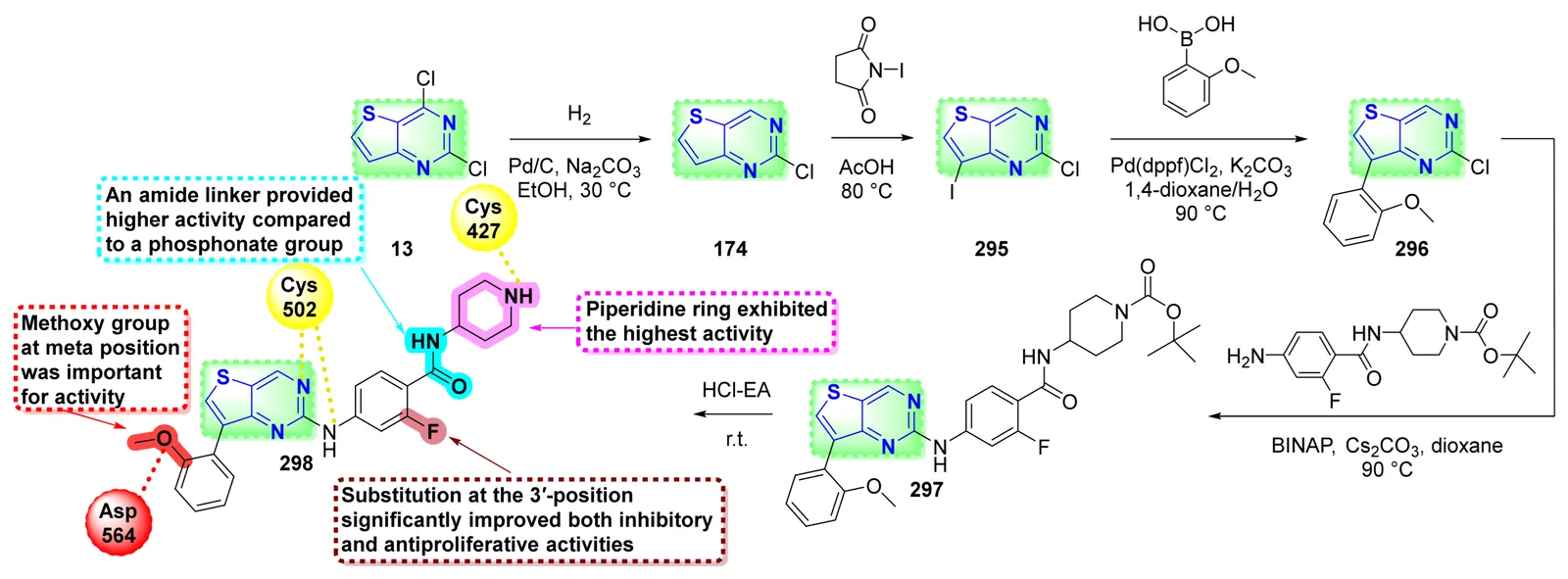
Structure and synthetic route of lead compound **298**.

A lead compound **298** was synthesized based on a 2,7-disubstituted-thieno[3,2-*d*]pyrimidine scaffold, employing regioselective dechlorination, iodination, and Suzuki coupling with (2-methoxyphenyl)boronic acid [208]. Subsequent Buchwald cross-coupling and acidic Boc deprotection afforded the final product (Scheme 66). In comparison to TAE-226, compound **298** showed markedly stronger growth-inhibitory effects in U-87MG, A549, and MDA-MB-231 cell lines, with IC_50_ values ranging from 0.16 to 0.27 µM. At 1 µM, it suppressed the activity of several oncogenic kinases—ALK, BTK, CDK2, FAK, and RET—by over 80%, indicating broad-spectrum kinase inhibition. Notably, compound **298** showed strong enzymatic inhibition toward FAK, as evidenced by its IC_50_ value of 28.2 nM. In MDA-MB-231 cells, **298** increased apoptosis with rising doses. Flow cytometry showed more cells in G0/G1 and fewer in G2/M. These effects align with its role in disrupting cell growth. SAR analysis showed that replacing the phosphonate group with an amide linker improved biological activity. Piperidine-containing analogs demonstrated the highest potency. A meta-methoxy substituent further increased activity, and substitution at position 3 of the phenyl on the pyrimidine ring significantly enhanced both inhibitory and antiproliferative effects.

### 3.12. Apoptosis-Related Anticancer Activity (Caspase-9)

**Scheme 67:**
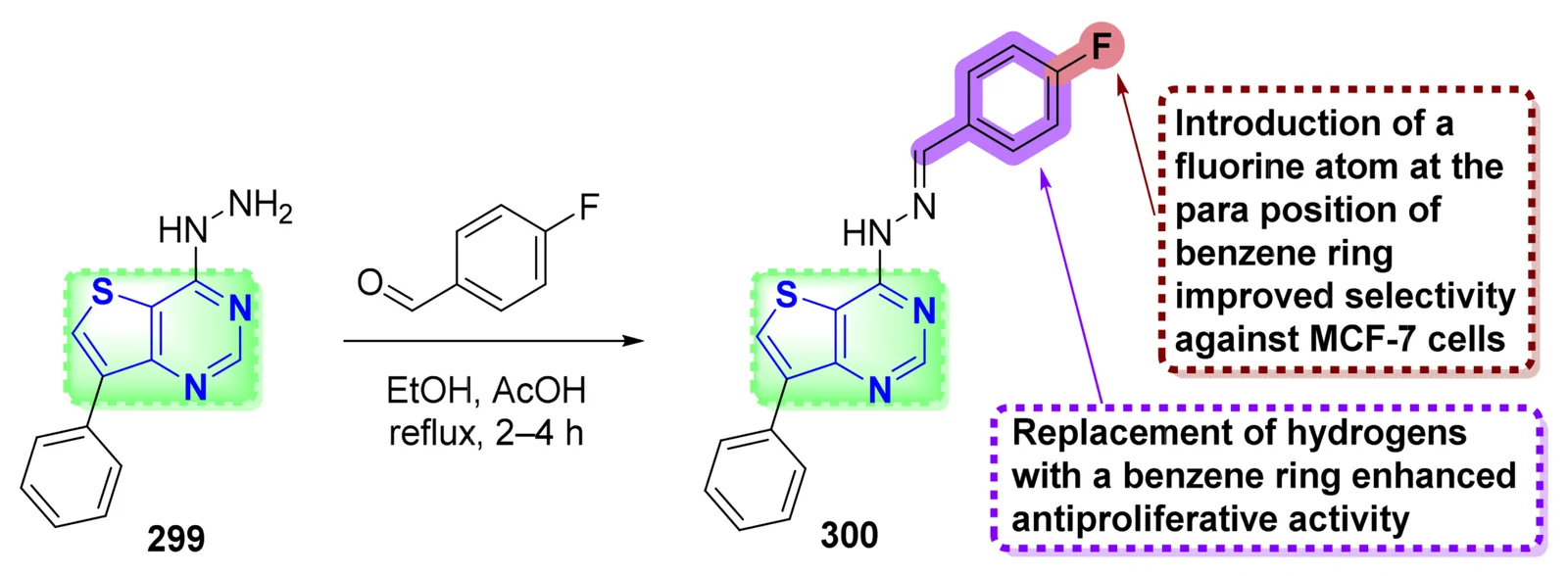
Structure and synthetic route of lead compound **300**.

Farghaly et al. [63] investigated thieno[3,2-*d*]pyrimidine derivatives for their ability to inhibit the proliferation of the MCF-7 breast cancer cell line. Compound **300**, which was formed by adding 4-fluorobenzaldehyde to the hydrazine of thienopyrimidine (Scheme 67), exhibited higher activity. It showed an IC_50_ of 0.43 μM and had the most significant effect on caspase-9 activation. Treatment with compound **300** increased caspase-9 levels to 0.066 μM and increased the percentage of cells in the G2/M phase from 18.7% to 49.1%. Furthermore, the percentage of apoptotic cells increased from 2.15% to 29.24%. Annexin V-FITC/PI assay results confirmed that **300** treatment resulted in significant apoptosis, suggesting that **300** could be a promising candidate for developing new apoptosis inducers for cancer therapy. SAR analysis demonstrated that incorporation of a benzene ring markedly enhanced antiproliferative activity compared with unsubstituted analogs. Furthermore, the introduction of a para-fluoro substituent on the benzene ring improved selectivity toward MCF-7 cells, indicating that electronic modification of the aromatic moiety positively influenced biological performance.

### 3.13. Miscellaneous Anticancer Targets of Thieno[3,2-d]pyrimidine Leading Compounds

**Scheme 68:**
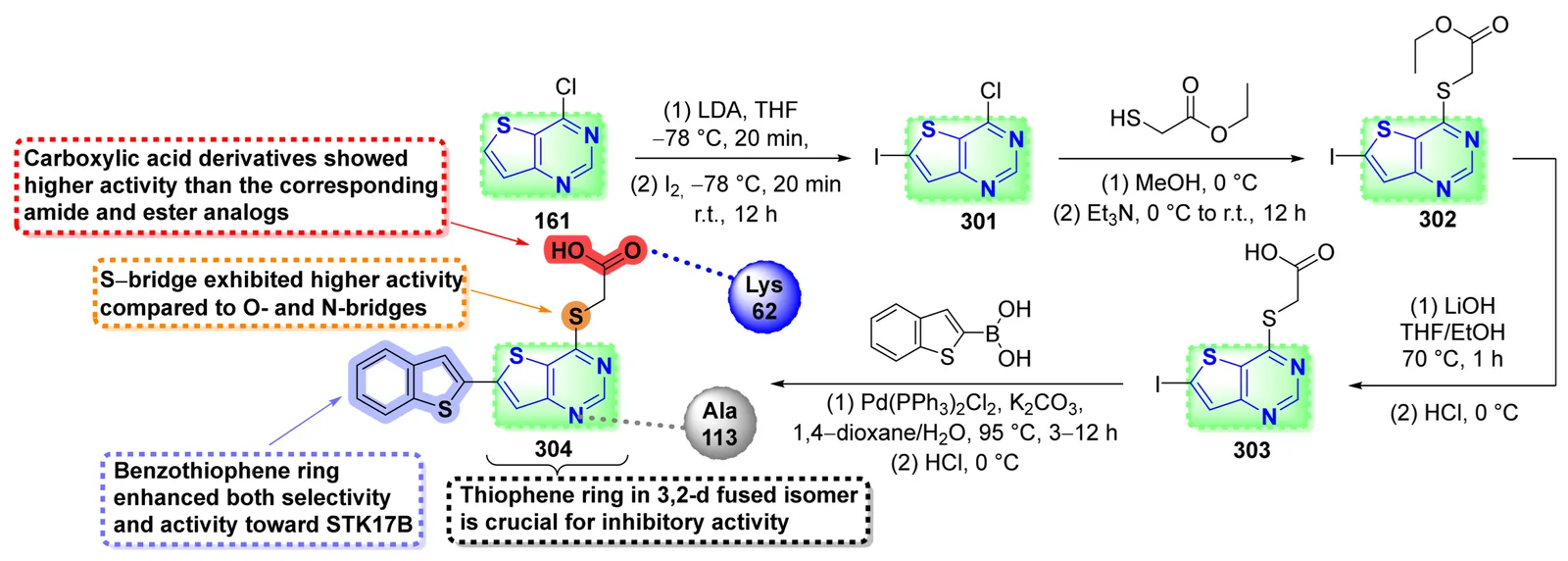
Structure and synthetic route of lead compound **304**.

A series of thienopyrimidine derivatives bearing fused aryl and heterocyclic moieties were synthesized and evaluated for their kinase selectivity by Picado and colleagues [209]. Starting from 4-chlorothieno[3,2-*d*]pyrimidine **161**, iodination at the C-6 position followed by reaction with ethyl 2-mercaptoacetate yielded intermediate **302**. Subsequent hydrolysis and Suzuki coupling with benzo[*b*]thiophen-2-ylboronic acid afforded the target compound **304** (Scheme 68). This molecule displayed potent inhibition of STK17B, with an IC_50_ of 190 nM in the NanoBRET assay and remarkable selectivity (IC_50_ = 34 nM) over related kinases. Broad screening revealed >75% ligand displacement at 1 μM for eight kinases, but **304** maintained strong selectivity, showing IC_50_ > 10 μM for most off-targets except CAMKK2 (IC_50_ = 2.4 μM). SAR analysis found carboxylic acid derivatives were more active than amide or ester analogs. Sulfur-bridged compounds were also more potent, indicating better target interactions. Adding benzothiophene improved selectivity and STK17B inhibition, while the thieno[3,2-*d*]pyrimidine scaffold was essential for potency.

**Scheme 69:**
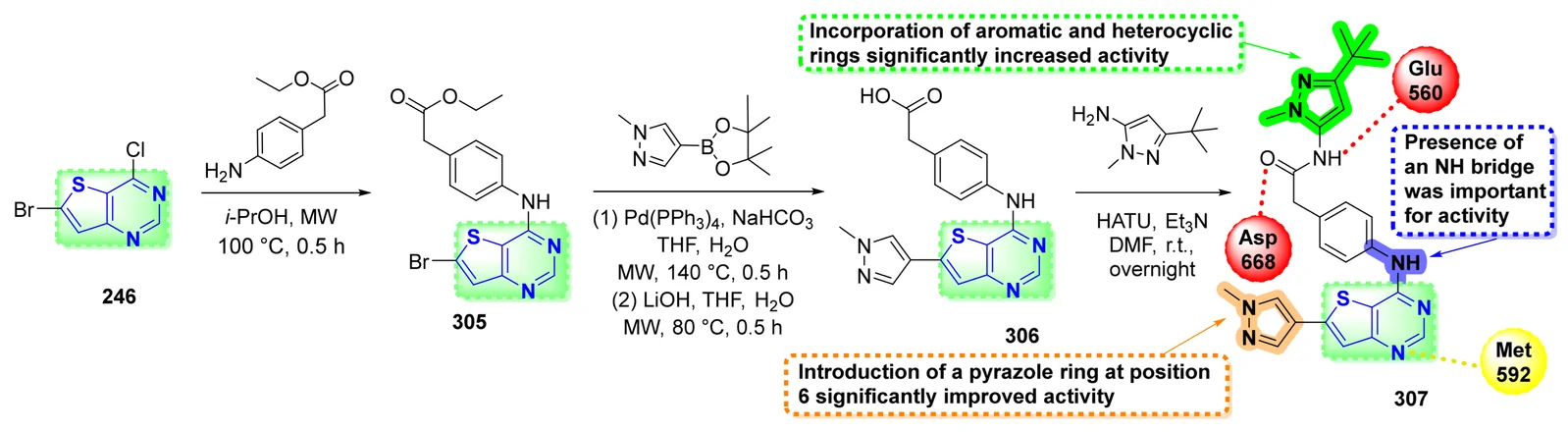
Structure and synthetic route of lead compound **307**.

Yan’s group [210] investigated the KM12 cell line to identify new lead compounds that inhibit TRK oncoproteins, especially TRKA autophosphorylation. Compound **307**, which contains a methylpyrazole, showed significant improvements in enzymatic (20-fold) and cellular (150-fold) potencies. Compound **307** effectively inhibited TRKA autophosphorylation at low concentrations and demonstrated selectivity, with IC_50_ values of 0.075 µM for TRKA and 0.074 µM for TRKB; it exhibited reduced activity against other kinases. Moreover, compound **307** demonstrated antiproliferative activity against KM12 cells (IC_50_ = 0.14 mM). An in vitro blood–brain barrier (BBB) assay revealed that entrectinib (Pe = 1.02 × 10^−3^ cm/min) and compound **307** (Pe = 3.03 × 10^−3^ cm/min) can cross the BBB. Compound **307** exhibited higher permeability due to its lower molecular weight (500.6 Da) and higher LogP (5.15). The synthesis of **307** involved reactions starting from compounds **246** and ethyl 2-(4-aminophenyl)acetate to yield compound **304** using anhydrous DMFA, HATU, and microwave irradiation (Scheme 69). SAR analysis showed that adding aromatic and heterocyclic groups significantly improved biological activity. Introducing a pyrazole ring at the C-6 position further enhanced inhibitory potency. The NH linker was also important for maintaining favorable target interactions and overall activity. In summary, a new series of 2-(4-(4-(thieno[3,2-*d*]pyrimidin-4-ylamino)phenyl)acetamide) derivatives has been established as effective pan-TRK inhibitors.

**Scheme 70:**
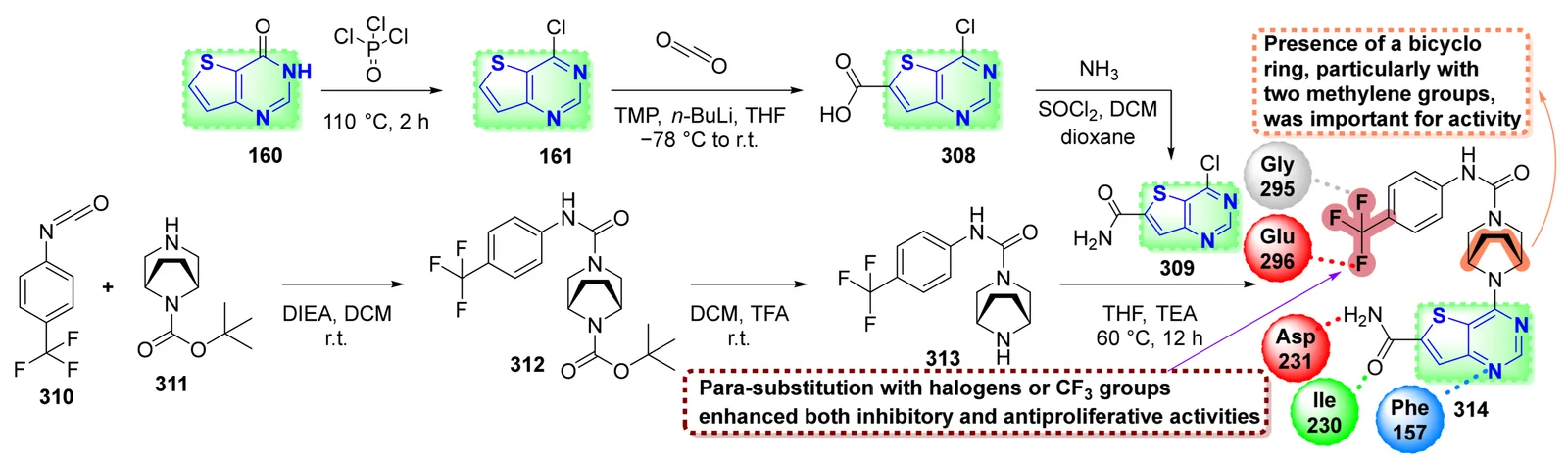
Structure and synthetic route of lead compound **314**.

SIRT3-targeting thienopyrimidine derivatives have been developed for acute myeloid leukemia (AML) therapy, and as a result, compound **314** was identified as a lead [211]. Its synthesis involved C-6 carboxylation and amidation or preparation via optical isomer intermediates (Scheme 70). Compound **314** showed potent SIRT3 inhibition (IC_50_ = 0.043 μM) and up to 98% activity at 10 μM. Compound **314** showed more potent cytotoxicity than 3-TYP in AML cell lines. In vivo studies revealed that it reduced tumor growth in HL-60-Luc models without causing adverse effects. SAR analysis showed that the bicyclic scaffold was important for biological activity. Derivatives with two methylene groups were more potent. Adding halogen or CF_3_ groups at the para position improved both inhibitory and antiproliferative effects, suggesting electron-withdrawing groups had a positive impact.

**Scheme 71:**
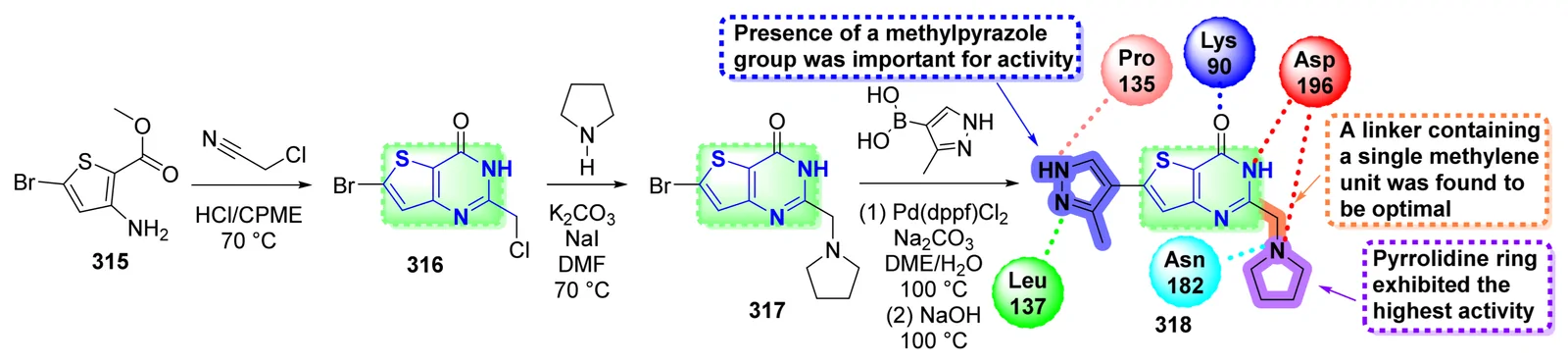
Structure and synthetic route of lead compound **318**.

Kurasawa et al. [212] synthesized a series of thieno[3,2-*d*]pyrimidin-4(3*H*)-one derivatives as selective Cdc7 kinase inhibitors. The scaffold was assembled by cyclization of methyl-3-amino-5-bromothiophene-2-carboxylate with chloroacetonitrile, followed by amination with pyrrolidine and Suzuki coupling with a pyrazolyl boronic ester to yield compound **318** (Scheme 71). Compound **318** showed potent inhibition of Cdc7 kinase (IC_50_ = 0.7 nM) with high selectivity over Cdk2/Cdc7 (14,000) and ROCK1/Cdc7 (200). Introduction of a 2-methyl group on the pyrrolidine ring improved both activity and selectivity. In cellular assays, derivative **318** reduced phosphorylation of MCM2 at Ser40 and inhibited COLO205 cell growth, with IC_50_ values of 250 nM and 1100 nM, respectively. SAR analysis showed that adding a methylpyrazole improved inhibitory activity. A single methylene linker gave the best results, while longer linkers reduced potency. Pyrrolidine-containing derivatives outperformed other cyclic amines, underscoring the importance of the pyrrolidine moiety for effective Cdc7 inhibition.

**Scheme 72:**
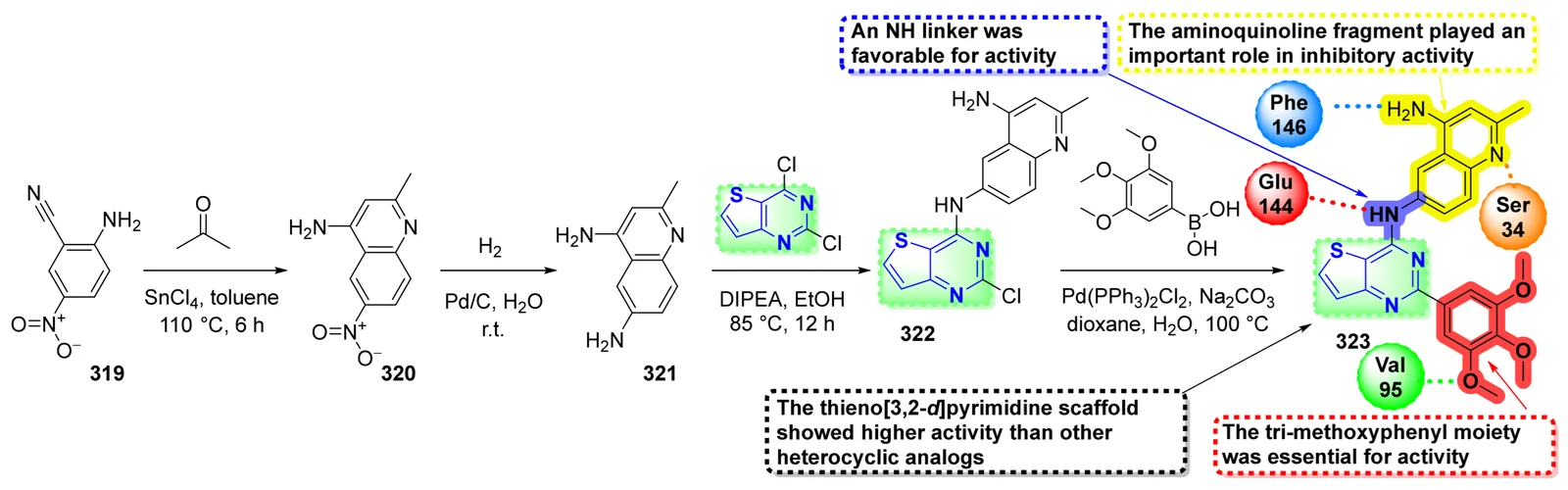
Structure and synthetic route of lead compound **323**.

A novel PRMT7 inhibitor, **323**, was synthesized by Li and co-authors [213] for prostate cancer therapy. The compound was prepared via cyclization of aminonitrobenzonitrile with acetone, followed by reduction, C-4 amide formation, and Suzuki coupling at C-2 (Scheme 72). **323** exhibited potent PRMT7 inhibition (IC_50_ = 0.50 μM), nearly 20 times more potent than JS1310. Tight binding was confirmed by KD = 0.32 μM, with π–π and π-sulfur interactions involving Tyr35 and Met38. In PCa cells, **323** showed IC_50_ values of 0.5–0.7 μM and almost complete colony suppression at 10 μM. It also induced G0/G1 arrest, apoptosis, and immunomodulatory effects. In vivo, **323** suppressed tumor growth by 62.4% with no toxicity up to 1000 mg/kg, highlighting its therapeutic potential. SAR studies showed that the presence of an NH linker was beneficial for activity. Introduction of the aminoquinoline fragment further improved inhibitory potency, while the thieno[3,2-*d*]pyrimidine scaffold displayed better activity than related heterocyclic analogs. In addition, the trimethoxyphenyl moiety was necessary to retain activity.

**Scheme 73:**
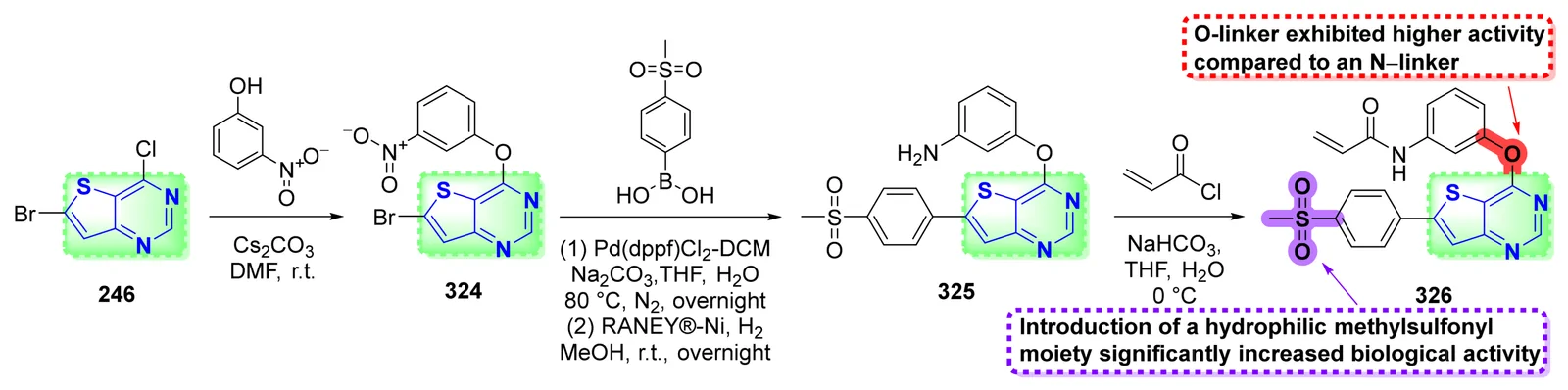
Structure and synthetic route of lead compound **326**.

A series of 4,6-substituted thieno[3,2-*d*]pyrimidines were synthesized via nucleophilic substitution, Suzuki coupling, nitro group reduction, and final acylation with acryloyl chloride to obtain compound **326** (Scheme 73) [214]. In biochemical assays, compound **326** showed potent inhibitory activity against Bruton’s tyrosine kinase (BTK), with an IC_50_ of 29.9 nM, approaching the potency of olmutinib (13.9 nM). It selectively suppressed B-cell proliferation (IC_50_ = 284 nM, SI = 188) with minimal effect on T cells and low cytotoxicity (CC_50_ > 50 µM). Compounds containing an *O*-linker displayed higher activity than the corresponding *N*-linked analogs, while incorporation of a hydrophilic methylsulfonyl moiety further enhanced biological activity. These results support compound **326** as a promising and selective BTK inhibitor with potential immunosuppressive applications.

**Scheme 74:**
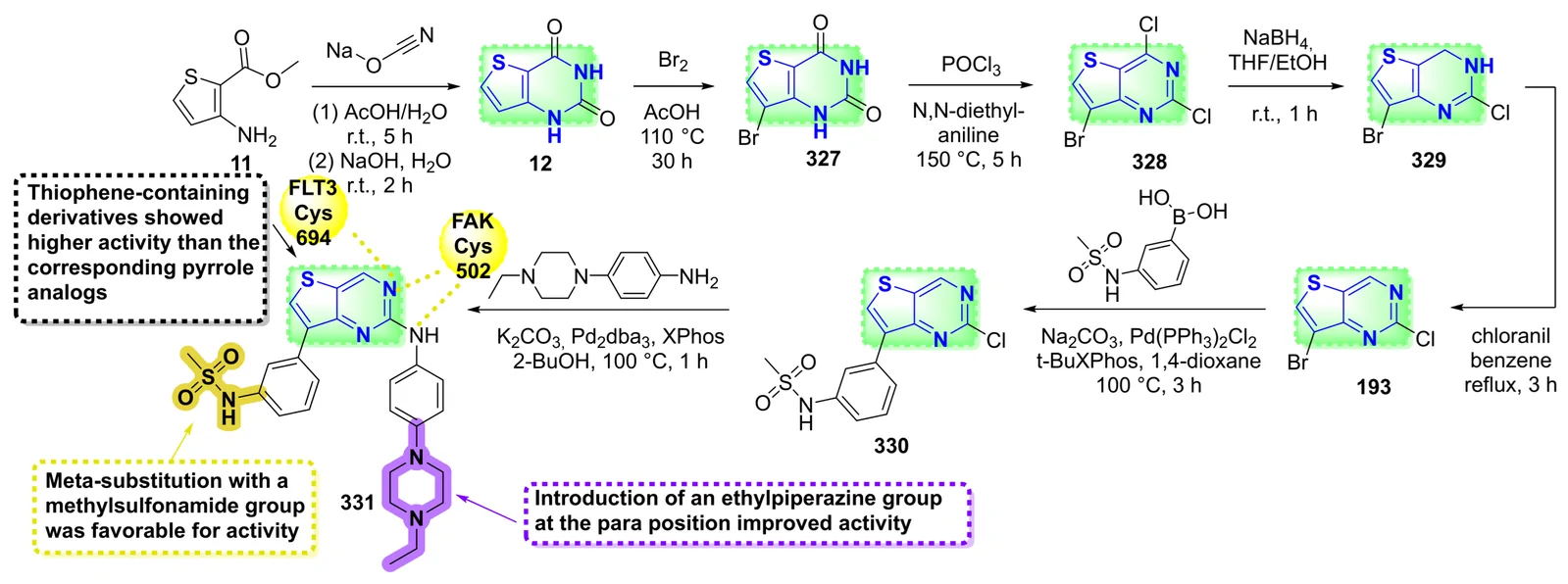
Structure and synthetic route of lead compound **331**.

Cho et al. [215] developed a series of thienopyrimidine derivatives starting from methyl 3-aminothiophene-2-carboxylate. Through cyclization, halogenation, and Suzuki coupling, they synthesized compound **331** by attaching aryl and piperazine groups (Scheme 74). This lead compound showed potent FAK inhibition (IC_50_ = 7 nM) and excellent metabolic stability. Hybrid **331** showed potent antiproliferative effects on human cancer cell lines, including HCT-116 (IC_50_ = 0.16 µM), MDA-MB-231 (IC_50_ = 0.47 µM) and A375 (IC_50_ = 0.37 µM). Additionally, it was effective against multiple FLT3 mutations, including D835Y and ITD. SAR analysis showed that thiophene-containing derivatives were generally more active than the corresponding pyrrole analogs. Activity was further improved by introducing a methylsulfonamide group at the *meta* position. In addition, para-substitution with an ethylpiperazine moiety provided the most favorable activity profile among the tested derivatives.

**Scheme 75:**
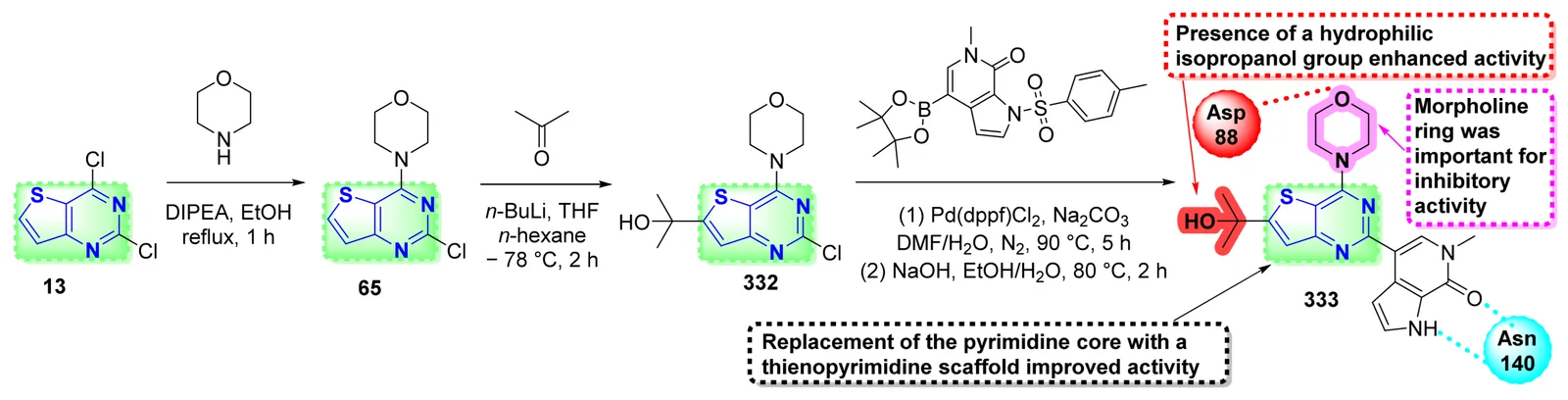
Structure and synthetic route of lead compound **333**.

Ran et al. [216] developed a series of 2- and 6-substituted morpholinothienopyrimidines as BET inhibitors, focusing on BRD4 selectivity. The synthesis of the lead compound **333** began with the deprotection of the C-6 position of morpholino-substituted intermediate **65** using n-BuLi and acetone to obtain compound **332**. This was followed by coupling with an *N*-Boc-protected heterocycle and subsequent deprotection to afford **333** (Scheme 75). The compound showed 95.0% inhibition of BRD4-BD1 at 20 nM and exhibited potent activity against BRD2-BD1, BRD3-BD1, BRD4-BD1, BRD4-BD2, and BRDT-BD1, with IC_50_ values ranging from 3.3 to 42.0 nM. Compound **333** also inhibited Jurkat and SU-DHL-4 lymphoma cells with an IC_50_ of 52.3 and 8.6 nM. Its potency was attributed to π–π stacking with Trp81, enabled by the pyrrolopyridone–morpholine scaffold. These findings support its potential as a BRD4-targeted anticancer agent. SAR studies indicated that the introduction of a hydrophilic isopropanol group improved activity, while the morpholine ring played an important role in inhibitory potency. In addition, thienopyrimidine-based derivatives displayed superior activity compared with the corresponding pyrimidine-containing analogs.

**Scheme 76:**
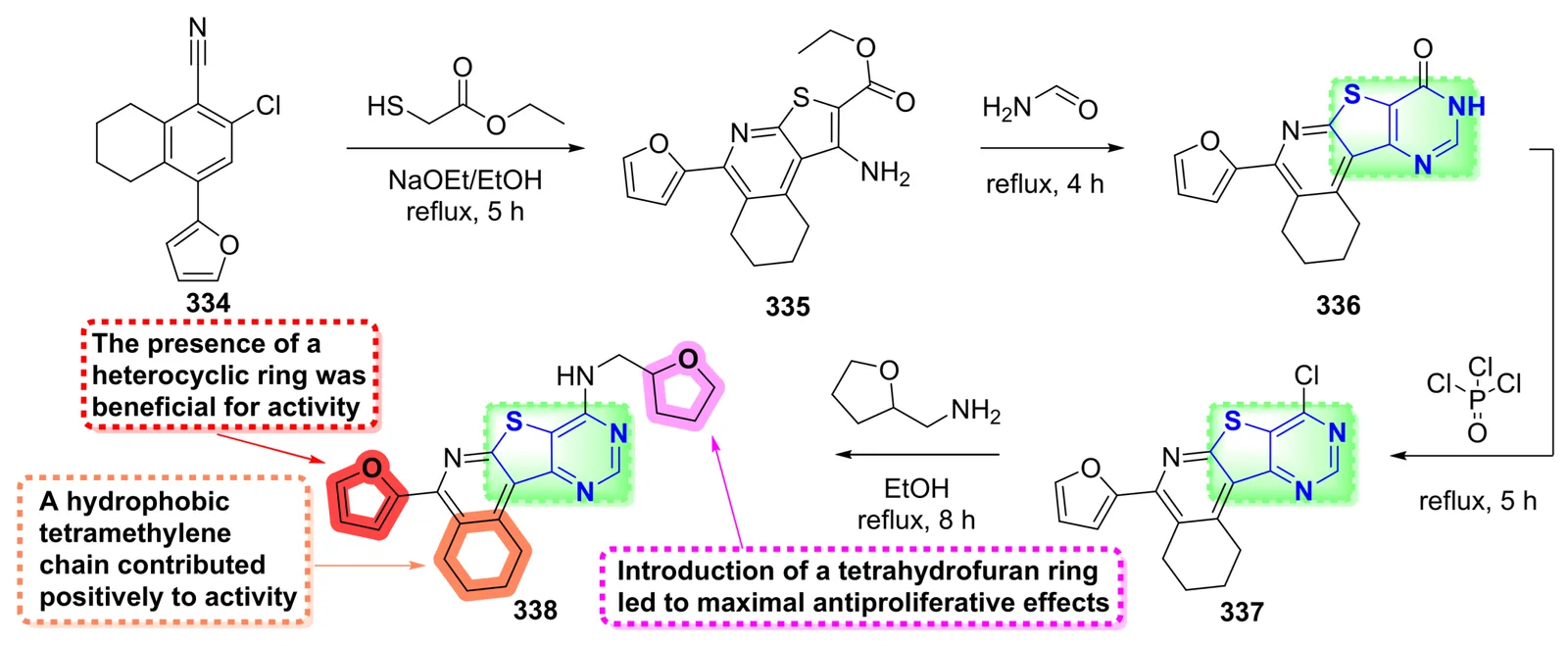
Structure and synthetic route of lead compound **338**.

Sirakanyan et al. [217] reported the synthesis of lead compound **338** via a multistep route starting from 3-chloro-1-(2-furyl)-5,6,7,8-tetrahydroisoquinoline-4-carbonitrile. Key transformations included substitution with ethylmercaptoacetate, cyclocondensation with formamide, chlorination, and final amination with various amines to yield **338** (Scheme 76). Compound **338** significantly reduced the methylation level of tumor DNA by 89.1%, exceeding the effect of doxorubicin (67.2%). It also showed notable cytotoxicity against HeLa cells (IC_50_ = 10.8 µM), indicating its potential as an antitumor agent with epigenetic activity. SAR analysis showed that incorporation of a heterocyclic ring was favorable for activity. Increased hydrophobicity provided by the tetramethylene chain further enhanced the overall activity profile. In addition, the introduction of a tetrahydrofuran moiety afforded the most potent antiproliferative effects among the evaluated derivatives.

**Scheme 77:**
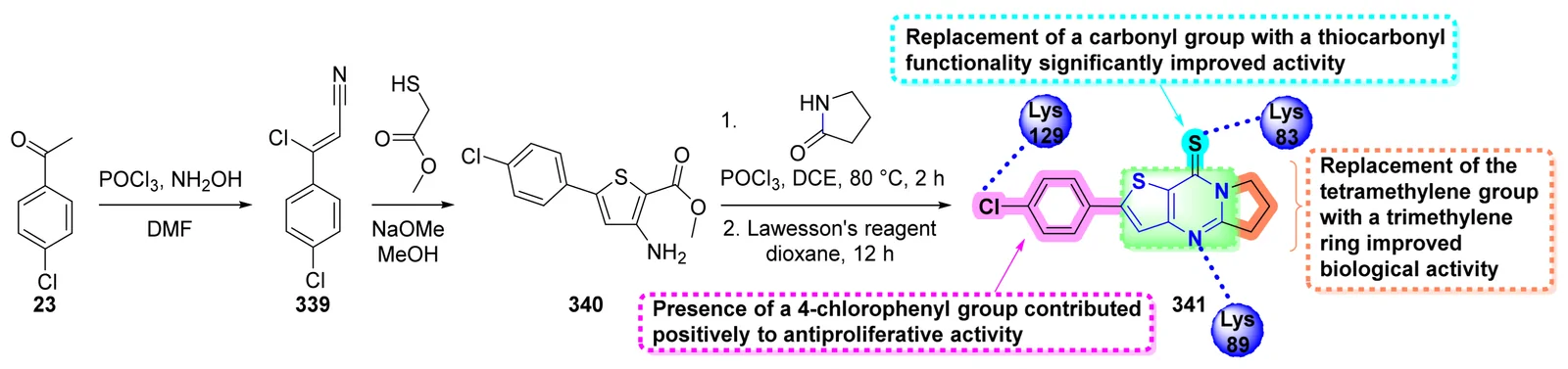
Structure and synthetic route of lead compound **341**.

Our research team recently studied the anticancer effects of new thieno[3,2-*d*]pyrimidin-4-one compounds [218]. To make these, we halogenated the carbonyl group, converted it to an acrylonitrile with hydroxylamine, and then used Lawesson’s reagent for intercyclization and thiation to create compound **341** (Scheme 77). This lead compound, which contains a 4-chlorophenyl group, showed the greatest cytotoxicity, reducing the growth of HeLa and HT-29 cells by 86% and 81%, respectively. The antiproliferative effect of **341** depended on both concentration and time (IC_50_ = 0.591 µM), and it caused G2/M phase arrest, increased reactive oxygen species, and disrupted microtubules. SAR analysis indicated that replacement of the carbonyl group with a thiocarbonyl moiety enhanced activity. The presence of a 4-chlorophenyl substituent was also important for antiproliferative effects. In addition, the incorporation of a tri-methylene ring further improved the biological activity of the synthesized derivatives. Molecular docking revealed that the lead derivative **341** forms stable hydrogen bonds and hydrophobic interactions with the ATP-binding sites of CDK2 and CDK5, suggesting that **341** is a selective CDK-targeted antiproliferative agent.

### 3.14. Mechanistic Insights and Target-Based Analysis of Thieno[3,2-d]pyrimidine Anticancer Agents

Studies on thieno[3,2-*d*]pyrimidine-based lead compounds over the past twenty years have shown several key mechanistic trends in anticancer drug development. Despite their structural variety and range of biological targets, these compounds mainly interact with ATP-dependent enzymes that play roles in cancer cell growth, survival, blood vessel formation, and DNA repair. As a result, thieno[3,2-*d*]pyrimidines are now seen as highly versatile scaffolds for kinase-targeted drug design in medicinal chemistry.

A major report in the literature is the focus on designing drugs that target kinases. Most active thieno[3,2-*d*]pyrimidine derivatives work by inhibiting protein kinases such as EGFR, PI3K, ATR, c-Met, VEGFR-2, JAK, and CDK family members. The core structure of thieno[3,2-*d*]pyrimidine closely resembles the adenine part of ATP, allowing it to fit well into kinase ATP-binding sites. Studies using docking, co-crystal structures, and enzyme tests have shown these compounds interact well with key amino acids in the catalytic domains, which explains their strong activity. Adding features like hydrogen-bond donors and acceptors, morpholine groups, urea linkers, and other ring structures has often improved how well these compounds bind and inhibit their targets.

EGFR is one of the most studied targets among these compounds. Some derivatives, including compounds **87**, **95**, **100**, **106**, and **118**, were made to address resistance caused by important EGFR mutations like T790M, L858R, Del19/T790M, and C797S. To achieve this, researchers often added acrylamide groups that can form irreversible bonds with the target. Docking studies showed these compounds (for example, lead compound **87**) make strong hydrophobic and hydrogen-bond interactions with residues such as MET790, GLN791, MET793, and SER797. As a result, many of these compounds are highly selective for mutant EGFR over the normal form, showing that thieno[3,2-*d*]pyrimidine scaffolds are well-suited for overcoming resistance to earlier EGFR inhibitors.

Particular attention has been devoted to modulation of the PI3K/AKT/mTOR cascade, one of the most frequently investigated signaling pathways among the reviewed studies. Many of the compounds, including leads **126**, **134**, **136**, **145**, and **149**, were designed to selectively inhibit PI3Kα, PI3Kδ, or both PI3K and mTOR. Herein, derivatives with morpholine groups were especially successful, often showing strong enzyme inhibition and anti-cancer activity at very low concentrations. Blocking PI3K signaling led to reduced activation of AKT and mTOR, which in turn lowered cancer cell survival and growth. Some compounds also reduced phosphorylation of AKT and P70S6K, confirming they effectively block this cancer-related pathway. These results show that thieno[3,2-*d*]pyrimidines fit well with PI3K targets and are promising for developing new pathway-focused cancer drugs.

Another common mechanism seen in these studies is cell-cycle arrest and the induction of apoptosis. Many lead compounds caused programmed cell death through various pathways and also disrupted the cell cycle. Depending on their targets, these compounds caused cells to stop at different points in the cycle, such as G0/G1, G1, or G2/M, which helped prevent uncontrolled cancer cell growth. For example, compound **136** primarily induced G1-phase arrest, while compounds **157**, **209**, **300**, and **341** were associated with accumulation of cells in the G2/M phase. ATR inhibitors blocked DNA repair and responses to replication stress, while compounds targeting tubulin interfered with microtubule formation and mitotic spindle assembly, leading to cell death. Several compounds increased the number of apoptotic cells and activated caspase pathways, showing that triggering apoptosis is a common result of thieno[3,2-*d*]pyrimidine target inhibition.

Although kinase inhibition dominates the field, several investigations have expanded the biological scope of thieno[3,2-*d*]pyrimidines toward non-kinase targets. For example, tubulin inhibitors from this group (leads **152**, **157**, and **159**) showed strong antimitotic and antiproliferative effects at very low concentrations, while HDAC inhibitors (**205** and **209**) affected gene regulation and promoted cell death. Other examples include inhibitors of BET bromodomains, PRMT7, SIRT3, PARP, and other new cancer-related proteins. These results suggest that thieno[3,2-*d*]pyrimidines have much broader potential and could help develop new treatments that target a range of cancer processes.

There is also a growing focus on designing drugs that target more than one protein at the same time. Some of the most active compounds were able to inhibit two or more important targets, such as EGFR/PI3K, EGFR/ErbB-2, PI3K/mTOR, ATR/mTOR, PARP/PI3K, BET/PI3K, JAK/BTK, and PI3K/PIM/mTOR. These multitarget strategies are meant to block several signaling pathways at once, improve treatment results, and lower the risk of drug resistance. The success of these hybrid compounds shows that the thieno[3,2-*d*]pyrimidine core is flexible and can include different functional groups without losing its activity.

Thus, the studies reviewed show that thieno[3,2-*d*]pyrimidines are valuable scaffolds for anticancer drugs because they can affect many cancer-related pathways. Although most work so far has focused on kinase inhibition, new research on epigenetic regulators, proteins involved in cell death, and multitarget approaches suggests there are many more possibilities for future drug development. Exploring new targets, allosteric effects, covalent inhibitors, and multitarget designs could further increase the potential of thieno[3,2-*d*]pyrimidines in cancer therapy.

## 4. Comparative SAR Analysis and Medicinal Chemistry Perspectives

Analysis of the lead compounds shows that the anticancer activity of thieno[3,2-*d*]pyrimidines depends on attached heterocycles, linker units, and substituents (Table 2). Nitrogen- and sulfur-containing heterocycles like morpholine, piperazine, piperidine, imidazole, triazole, indazole, and quinazoline are common in the most active derivatives. These parts often improve solubility and promote favorable interactions within kinase ATP-binding pockets and enzyme sites. Linker unit design is important for the scaffold-hopping strategy. In the thieno[3,2-*d*]pyrimidine molecule, fragments such as an amide, urea, carbamate, hydrazide, and ester groups acted as both connectors and pharmacophoric units, demonstrating hydrogen bonds and electrostatic interactions. In various series of thieno[3,2-*d*]pyrimidine scaffolds, methylene bridges (-CH_2_-) and alkyl spacers connect the thieno[3,2-*d*]pyrimidine core to other heterocyclic systems, providing structural flexibility for different targets. In addition, substituent effects revealed that electron-withdrawing groups such as halogens and a cyano group improve potency and selectivity, due to electronic effects and stability. Fluoro- (F) and trifluoromethyl (CF_3_) analogs are demonstrated to have better potency among the other studied derivatives. Electron-donating groups such as methoxy (OCH_3_), hydroxy (OH), and alkyl enhanced activity by boosting hydrophobic contacts or hydrogen bonds, depending on their position. In addition, acrylamide and propenone fragments, often linked to covalent or semi-covalent inhibition, are significant in kinase-targeted drug design. Among the sulfur (-S-) containing substituents or fragments, sulfonyl and sulfonamide groups are common in active analogs, providing selectivity and ligand stability.

Indeed, hybridizing the thieno[3,2-*d*]pyrimidine core with aromatic and heteroaromatic or other heterocyclic ring systems is a major and needed point in medicinal chemistry. In these scaffolds, introducing indole, quinoline, isoquinoline, benzofuran, and benzo[*d*][1,3]dioxole fragments increases rigidity and π-conjugation, enhancing interactions with hydrophobic and allosteric sites of appropriate targets. Some studies show amino acid-derived and bicyclic amine fragments further improve cellular selectivity.

Beyond the compound-specific SAR summarized in Table 2, comparison of different thieno[3,2-*d*]pyrimidine series reveals several common trends. The fused thieno[3,2-*d*]pyrimidine core provides a suitable framework for ATP-site binding, while peripheral substituents play an important role in determining target preference and selectivity. This combination has allowed the scaffold to be adapted to different kinase targets, including EGFR, PI3K, CDKs, ATR, VEGFR, and JAK.

The PI3K-targeted series provides a clear example. Morpholine groups are present in many active PI3K inhibitors, but they do not determine isoform selectivity alone. Compound **126**, containing morpholine and indazole groups together with hydrophilic substitution, showed potent PI3Kα inhibition. In contrast, the piperazinone-containing compound **131** showed marked PI3Kδ selectivity, with short alkyl or cycloalkyl substituents favoring its activity. Compound **145** also showed a clear preference for PI3Kδ, where the chiral fused triazolonaphthyridine system and indole group contributed to potency and selectivity. These examples show that PI3K isoform selectivity depends on the overall arrangement, stereochemistry, hydrophobicity, and hydrogen-bonding properties of groups surrounding the thieno[3,2-*d*]pyrimidine core.

A similar trend is observed in the EGFR-directed series, where peripheral modification has been used to address resistance-associated mutations. In particular, appropriately positioned acrylamide groups provide covalent binding in several mutant EGFR inhibitors, demonstrating how modification outside the heteroaromatic core can adapt the scaffold to resistance-associated changes in the binding site.

Overall, these SAR trends show that high potency alone does not guarantee selectivity. Since ATP-binding sites are highly conserved among kinases, interactions with less-conserved regions around the ATP pocket can play an important role in improving selectivity. Hydrophobic substituents may strengthen interactions within these regions, while polar groups exposed to the solvent can help improve physicochemical properties. In the case of resistant kinase variants, additional functional groups, such as covalent warheads, may also be useful for maintaining inhibitory activity.

A number of reported thieno[3,2-*d*]pyrimidine derivatives have concentrated around several well-designed scaffolds and target classes, particularly kinase inhibition. It was observed that much attention has been dedicated to PI3K-directed compounds, especially analogs related to pictilisib (GDC-0941). Such approaches have produced potent inhibitors and valuable SAR information; they have also led to repeated structural modifications around similar pharmacophoric designs.

At the same time, the literature reports demonstrate that the thieno[3,2-*d*]pyrimidine nucleus possesses a range of design potential than currently explored. Over the past 15–20 years, numerous hybrid systems incorporating various heterocycles, as well as urea, sulfonamide, and other pharmacophoric fragments, have been synthesized, many of which displayed promising anticancer properties and improved target interactions. These observations suggest that the future development of thieno[3,2-*d*]pyrimidines may serve not only as a source of known kinase inhibitors but also for the design of structurally major hybrid scaffolds and exploration of new biological targets. From this perspective, thieno[3,2-*d*]pyrimidines remain a chemically key synthone and pharmacologically promising scaffold for continued anticancer drug discovery.

Selectivity is a key challenge when developing thieno[3,2-*d*]pyrimidine kinase inhibitors. Since the ATP-binding site is very similar across human kinases, compounds that compete with ATP can often block more than one kinase. This is helpful for drugs meant to target several kinases, but unwanted inhibition can lead to side effects. The studies in this review include both strategies: some compounds were designed to block specific kinases or isoforms, while others were made to inhibit multiple signaling proteins at once. Because of this, testing compounds against a wide range of kinases is important to tell whether they are truly selective or just highly potent against one target.

Resistance is also a major concern when designing kinase inhibitors. Changes in or near the ATP-binding site can make it harder for inhibitors to bind, which reduces the effectiveness of drugs that once worked well. The EGFR-targeted thieno[3,2-*d*]pyrimidines mentioned earlier show that changing a compound’s structure can help keep it active against resistant forms. Still, resistance can also develop through other signaling pathways or new mutations, so simply targeting a resistant kinase may not be enough for lasting cancer treatment. This highlights the need to keep exploring multitarget, allosteric, and other resistance-focused thieno[3,2-*d*]pyrimidine compounds.

It is important to consider physicochemical properties along with biochemical potency. Many active thieno[3,2-*d*]pyrimidines have aromatic or heteroaromatic rings and hydrophobic groups that help them bind to their targets. However, making a compound more hydrophobic can lower its water solubility, change how it is processed in the body, and increase unwanted binding. To balance this, polar groups like morpholine, piperazine, hydroxy, and amide are often added. Still, the studies in this review did not always report physicochemical and pharmacokinetic data in the same way, which makes it hard to directly compare different compound series.

Ligand efficiency (LE) and lipophilic efficiency (LipE) are useful during lead optimization because they show how biological potency relates to molecular size and lipophilicity. However, these values were rarely reported for the thieno[3,2-*d*]pyrimidine compounds in this review. Also, the studies used different enzymatic and cellular tests to measure activity, which makes it hard to compare results across studies. In the future, it would help if researchers reported potency along with basic physicochemical properties and efficiency measures, such as molecular weight, lipophilicity, solubility, LE, and LipE. This information would make it easier to tell if a compound’s high potency comes from good molecular recognition or just from being larger or more lipophilic.

## 5. Clinical Translation, Limitations, and Challenges

Despite the large number of potent thieno[3,2-*d*]pyrimidine derivatives reported in preclinical studies, only a limited number have progressed to clinical evaluation. This difference between preclinical activity and clinical development remains one of the main limitations of this compound class. Many of the leads discussed in this review show nanomolar enzyme inhibition or strong antiproliferative activity, but most have been evaluated primarily in biochemical assays, cancer cell lines, and, in fewer cases, animal models. Therefore, high potency alone cannot be considered sufficient evidence of clinical potential.

Pictilisib (GDC-0941) provides an important example of this transition. Its development established the thieno[3,2-*d*]pyrimidine framework as a useful platform for PI3K inhibition and stimulated extensive structural modification of this scaffold. As discussed above, subsequent studies produced derivatives with different PI3K isoform profiles and, in several cases, high biochemical and cellular potency. However, pictilisib itself progressed through clinical investigation without becoming an approved anticancer drug. This illustrates an important point for this chemical class: successful optimization of target potency and selectivity does not necessarily result in successful clinical development.

Olmutinib represents a different outcome. As a mutant EGFR inhibitor, it progressed to clinical use and demonstrated that a thieno[3,2-*d*]pyrimidine-related scaffold can be successfully developed against a clinically relevant resistance mutation. At the same time, serious adverse reactions reported during its development and clinical use raised important safety concerns. The contrasting development of pictilisib and olmutinib therefore shows that clinical translation depends not only on target inhibition but also on therapeutic window, tolerability, and sustained benefit in patients.

Pharmacokinetic evaluation represents another important gap between early lead discovery and clinical development. For many of the experimental compounds reviewed here, detailed information on absorption, systemic exposure, metabolic stability, clearance, and bioavailability is limited or unavailable. Consequently, it remains difficult to determine whether the strongest compounds identified in biochemical or cellular assays can reach and maintain effective concentrations in vivo. More systematic ADME and pharmacokinetic evaluation at an earlier stage of lead optimization would help identify compounds with greater potential for further development.

Overall, the available evidence shows considerable progress in the medicinal chemistry of thieno[3,2-*d*]pyrimidines, but a much smaller number of compounds have demonstrated clinical viability. Future studies should therefore place greater emphasis on the transition from potent leads to developable candidates, with early evaluation of in vivo exposure, safety, tolerability, and therapeutic window alongside anticancer efficacy.

To provide an integrated perspective on the therapeutic potential of the compounds discussed above, the most representative thieno[3,2-*d*]pyrimidine anticancer leads are comparatively summarized in Table 3. The comparison highlights their molecular targets, potency and selectivity profiles, and current stages of development, thereby allowing the most advanced and pharmacologically promising representatives of this scaffold to be readily identified.

## 6. Conclusions

In summary, thieno[3,2-*d*]pyrimidine-based hybrid derivatives have emerged as a significant focus of research in the development of anticancer drugs over the past two decades. Studies discussed in this review demonstrate that this heterocyclic scaffold can be modified in various ways and adapted to target a broad range of cancer-related proteins, including EGFR, PI3K/mTOR, CDKs, JAK, VEGFR, HDAC, and ATR. Many of the reported compounds demonstrated strong antiproliferative activity, and several lead molecules showed nanomolar inhibitory potency and promising results in preliminary in vivo studies. SAR analysis indicates that the biological activity of thieno[3,2-*d*]pyrimidine derivatives is strongly influenced by the nature of the attached heterocycles, linker fragments, and electronic substituents. The introduction of *N*- and *S*-containing heterocycles in the thieno[3,2-*d*]pyrimidine core, including morpholine, piperazine, triazole, imidazole, and quinazoline, often improves potency and selectivity. Polar linkers, including amide, urea, carbamate, hydrazide, and sulfonamide groups, also play an important role in strengthening interactions with biological targets. On the other hand, most thieno[3,2-*d*]pyrimidine leads are still in the early stages of development. Further scientific research is needed to better understand their pharmacokinetic properties, toxicity, metabolic stability, and oral bioavailability. The identification of new molecular targets in these derivatives may serve the therapeutic potential of this scaffold.

Thus, available studies suggest that thieno[3,2-*d*]pyrimidines are promising scaffolds for the development of new targeted anticancer agents. Their structural diversity, broad biological activity, and compatibility with different medicinal chemistry strategies continue to make them promising compounds for future anticancer drug research.

## Figures and Tables

**Table 1:** Scope and coverage of previously published thienopyrimidine reviews.

Reference	Published Year	Literature Coverage	Main Focus	Isomer/Scaffold Coverage	Difference and Relation to Present Review
Farag et al. [85]	2024	2013–2023	VEGFR inhibition; aryl urea thienopyrimidines	[2,3-*d*] isomer	Target-oriented review; not dedicated to thieno[3,2-*d*] anticancer chemistry
Nadar et al. [86]	2025	Not specified	Synthesis, SAR, clinically investigated analogs, and diverse biological activities	All three isomers ([2,3-*d*]; [3,2-*d*]; [3,4-*d*])	Broad scaffold review; not specifically focused on thieno[3,2-*d*]pyrimidines or anticancer medicinal chemistry
Ghith et al. [87]	2017	Not specified	Tyrosine kinase-targeting thienopyrimidines and anticancer activity	[2,3-*d*] and [3,2-*d*] isomers	Target-oriented anticancer review; not specifically focused on thieno[3,2-*d*]pyrimidines
Rabeh et al. [88]	2026	2007–2025	Green and sustainable synthetic approaches	[2,3-*d*] and [3,2-*d*] isomers	Synthesis-focused review; not dedicated to anticancer medicinal chemistry or thieno[3,2-*d*] biological applications
Liu et al. [89]	2025	Not specified	Kinase inhibitors, anticancer activity, and SAR	[2,3-*d*] and [3,2-*d*] isomers	Kinase-oriented anticancer review; not specifically focused on thieno[3,2-*d*]pyrimidines
Sayed et al. [90]	2023	2014–2022	Synthesis and anticancer activity	[2,3-*d*] and with limited [3,2-*d*] isomers	Anticancer-focused review; limited coverage of thieno[3,2-*d*]pyrimidines (only 4 leading compounds is discussed)
Lagardère et al. [91]	2022	Not specified	Anti-infective activity, SAR, and synthesis	All three isomers ([2,3-*d*]; [3,2-*d*]; [3,4-*d*])	Anti-infective-focused review; not dedicated to anticancer or thieno[3,2-*d*] medicinal chemistry
Islam and Quadery [92]	2021	Not specified	Therapeutic potential, synthesis, patents, and SAR	Dedicated to thieno[3,2-*d*]pyrimidines	Broad thieno[3,2-*d*] review; not specifically focused on synthesis of anticancer leads

**Table 2:** Summary of SARs of the thieno[3,2-*d*]pyrimidine-based lead compounds are discussed in this review.

Lead Compounds	Rings and Fragments Influencing Activity	Groups/Sections Enhancing Activity	Pharmacophore Substituents	Lead Compounds	Rings and Fragments Influencing Activity	Groups/Sections Enhancing Activity	Pharmacophore Substituents
**3**	Pyridine, Imidazole-4,5-dione	Amino, Carbonyl	-CH_3_	**180**	Isoxazole	Amide, Phenyl	-OCH_3_
**7**	Thiazine	Phenyl, Hydroxy	-OCH_3_, -NH_2_, -CN	**187**	Thieno[2,3-*b*]pyridin, Piridine	Amine	-CH_3_
**10**	Benzylidene	-N=CH-, CH=N-NH-	-Br, -OH,	**192**	Indole, Piperidine	-CH_2_-	Ph
**13**	-	-	-Cl	**196**	Indolinone Piperidine	Amine	spirocyclopropane
**17**	Imidazole	Tetrahydrofuran	-OH, NH_2_	**199**	Indole, Piperidine	Amine	-F, -CN, -PO(CH_3_)_2_
**22**	Phenyl	Amide, Amino	-NH_2_	**205**	N-hydroxyamide	Methylene bridge, Amide	-CN, -OH
**27**	Pyridine	Phenyl	-NO_2,_ -Cl_2_	**209**	Morpholine, N-hydroxyamide	Methylene bridge, Ester	-OH
**30**	Pyrazole, Pyridine	Phenyl	-NO_2_	**213**	Pyrazole, Piperazine,	Methylene bridge, Amide,	-OCH_3_, -OH, -CH_3_
**35**	Phenyl	Sulfonamide, Amide	-F, -Cl,	**221**	Acrylamide, Morpholine,	Methylene bridge, Ester	-CH_3_
**39**	Thiophene	Amide	-CH_3_	**225**	Morpholine	Urea,	-F, -cyclopropyl
**43**	Phenyl	Amide	-Cl	**228**	Pyrimidine	Hydrazide	-NH_2_, -OCH_3_
**45**	Phenyl	Amide	-Cl	**230**	Morpholine,	Phenyl	-OH
**54**	Acryl	Amide	-OCH_3_	**235**	Morpholine	Azomethine, Urea	-CH_3_, -OH
**60**	Aniline	Amide	-Cl, -NH_2_	**237**	Morpholine,	Pyrazole	-CH_3_
**64**	Triazole Imidazole	Amide, Methylene bridge	-Ph	**238**	Morpholine, Indazole	Piperazine	-S(O)_2_-CH_3_
**69**	Morpholine Indole	-NH-N=CH-	-F	**239**	Morpholine, Pyrimidine	*i*-Propanole	-NH_2_
**75**	Benzo[*d*][1,3]dioxol, Morpholine	Piperazine, -NH-N=CH-	-S(O)_2_-CH_3_	**240**	Morpholine, Pyrimidine	*i*-Propanole	-NH_2_, -CH_3_
**79**	Morpholine Pyrazine	Amine	-N(CH_3_)_2_	**245**	Morpholine, Pyrimidine	Piperazine	-OH, -NH_2_, -CH_3_
**84**	D-phenylalanine	Phenyl	-I	**249**	Furan	Ester, Amine	-S(O)_2_-CH_3_, -F
**87**	Piperazine	-N-CH_2_-	-OH	**252**	Pyrrolidine	Carbamate, Ester, Ethynyl	-F, -Cl, -C_2_H_5_
**91**	Acrylamide, Piperazine	Ester	-I. -CH_3_	**256**	Phenyl	Amine	-CH_3_, -OCH_3_
**95**	Acrylamide	Ester	-CN	**268**	Imidazole, Oxazole	Piperazine, Methylene bridge, Ester	Acrylamide, -NH_2_
**100**	Quinoline	Bipiperidine	-F	**272**	Isoquinoline	Methylene bridge, Amine	-CH_3_, -OH
**106**	Thieno[3,2-c]pyridin, Acrylamide	Aminde, Amine	-Cl, -N(CH_3_), -OCH_3_	**274**	Pyrrolo [2,3-c]pyridinone, Morpholine,	Piperidine	-OH, -CH_3_
**108**	Pyridine	Aniline	-Cl	**281**	Phthalazinone, Pyrimidine, Morpholine	Piperazine	-NH_2_, -F
**115**	Quinazoline, Piperidine	Methylene bridge, Ester	-CH_3_, -OCH_3_	**283**	Morpholine, Benzofuran, Pyrimidine	Piperazine	-NH_2_, -CH_3_
**118**	Piperazine	Piperidine, Ester	Propenone, -CH_3_	**288**	Morpholine, Acrylamide	Methylene bridge, Ester	-Cl
**126**	Morpholine, Indazole	Acrylamide	Methylene bridge, -OH	**294**	5-Thia-4,8-diaza, Thiophena	Amine, Amide	-F, -CH_3_ -OCH_3_
**131**	Imidazole, Morpholine	Piperazinone	Cyclopropanecarbonyl	**298**	Piperidine	Amide	-F, -OCH_3_
**134**	Triazole, Dimorpholino	-CH_2_-NH-	-	**300**	Phenyl	-CH=N-NH-	-F
**136**	Morpholine	-CH=N-NH-, Amide	-OH	**304**	Benzo[*b*]thiophen	Thio	-COOH
**143**	Diethylaminopropyl	Urea	-Cl, -C_2_H_5_	**307**	Pyrazole,	Amide, Amine	-*tert*-butyl, -CH_3_
**145**	Morpholine, Indole	Piperidine	Methyltriazole	**314**	Diazabicyclo [3.2.1]octane,	Carbamoyl,	-CF_3_, -CONH_2_
**149**	Pyrimidin, Morpholine	Oxetane	-NH_2_, -OCH_3_	**318**	Pyrrolidine, Pyrazol	-CH_2_-	-CH_2_-
**152**	Indole	Amine	-CH_3_	**323**	Quinoline	Amine	-OCH_3_, -NH_2_, -CH_3_
**157**	Quinoxaline	-	-Cl	**326**	Acrylamide	Phenyl	-S(O)_2_-CH_3_
**159**	Tolylamino	Methanone	-OCH_3_	**331**	Piperazine	Amine, Phenyl	-S(O)_2_-CH_3_, -C_2_H_5_
**163**	Triazole	Amide Ester	-F, -Cl, -CF_3_,	**333**	Morpholin, Pyrrolo [2,3-c]pyridinone	-C(CH_3_)_2_-	-OH, -CH_3_
**169**	Pyridazine	Amide Ester	-F, -CH_3_,	**338**	Furan, Isoquinoline,	Amine, -CH_2_-	-CH_2_-
**173**	Morpholine, Imidazole	Amine	-S(O)_2_, -CH_3_	**341**	Phenyl	Methylene ring	-Cl

**Table 3:** Comparative summary of representative thieno[3,2-*d*]pyrimidine anticancer leads, their molecular targets, potency/selectivity profiles, and development stages.

Lead Compounds	Molecular Target/Cancer Model	Potency and Selectivity	Development Stage
**3**	HepG2, MCF-7	IC_50_ = 10.16 μM (HepG2) and 32.7 μM (MCF-7)	Preclinical: in vitro
**7**	MCF-7, HepG2	IC_50_ = 6.16 μM (MCF-7) and 7.13 μM (HepG2)	Preclinical: in vitro
**10**	Caco-2 colorectal cancer cells	IC_50_ = 0.73 μM (Caco-2) vs. 3.61 μM in normal fibroblasts; SI = 5.8	Preclinical: in vitro
**13**	L1210, CEM, HeLa	IC_50_ = 0.67 μM (L1210), 5.2 μM (CEM), 3.9 μM (HeLa); apoptosis induction	Preclinical: in vitro
**17**	L1210, CEM, HeLa, HEL	HEL cells, IC_50_ = 12 μM	Preclinical: in vitro
**22**	MCF-7	IC_50_ = 2.04 nM against MCF-7	Preclinical: in vitro
**27**	MCF-7, HCT-116	IC_50_ = 11.25 μM (MCF-7)	Preclinical: in vitro
**30**	MCF-7, HepG2, Caco-2	IC_50_ = 4.20, 3.65, and 7.62 μM, respectively	Preclinical: in vitro
**35**	B-Raf^V600E^	B-Raf^V600E^ IC_50_ = 0.18 nM; cellular pERK IC_50_ = 4.6 nM; A375-X1/Colo205/HT29 EC_50_ = 2/5/24 nM; strong preference for mutant B-Raf	Preclinical: in vitro, PK and in vivo xenograft
**39**	U87-MG glioblastoma	IC_50_ = 3.38 μM	Preclinical: in vitro
**43**	MCF-7, HepG2, HCT-116	IC_50_ = 14.53, 12.27, and 15.75 μM, respectively	Preclinical: in vitro
**45**	MCF-7, HepG2, HCT-116	IC_50_ = 11.17, 9.33, and 10.63 μM, respectively	Preclinical: in vitro
**54**	NCI-H1975, Ramos, A431, SNU-16, NCI-H1581	IC_50_ values ranging from 0.6 to 2.6 μM	Preclinical: in vitro
**60**	HepG2	IC_50_ = 0.0196 μM	Preclinical: in vitro
**64**	NCI-60 cancer panel	Mean activity ~525 nM; HCT-116/OVCAR-8 < 10 nM, several other lines ~11–20 nM; LC_50_ > 100 μM	Preclinical: in vitro, preliminary in vivo tolerability
**69**	H460, HT-29, H226, SGC-7901, MDA-MB-231	IC_50_ = 0.23 μM (H460), 0.39 μM (HT-29), 0.91 μM (H226), 0.80 μM (SGC-7901), 1.11 μM (MDA-MB-231)	Preclinical: in vitro
**75**	H460, HT-29, MDA-MB-231	IC_50_ = 0.003, 0.42, and 0.74 μM, respectively; WI-38 IC_50_ = 7.4 μM	Preclinical: in vitro
**79**	A2780 and other cancer cell models	A2780, IC_50_ = 8.6 μM	Preclinical: in vitro
**84**	HepG2, MCF-7, HeLa	IC_50_ = 5.39, 2.99, and 9.98 μM, respectively	Preclinical: in vitro
**87**	EGFR^Del19/T790M/C797S; EGFR^L858R/T790M	A549 IC_50_ = 0.77 μM; 98.9% inhibition of triple-mutant Ba/F3 cells at 10 μM; mutant EGFR inhibition favored over WT	Preclinical: in vitro, in vivo xenograft; TGI ≈ 55.9%
**91**	EGFR-mutant NSCLC models, particularly L858R/T790M	IC_50_ = 10.49 μM in the L858R/T790M-associated model and 14.55 μM for the L858R-associated model; weaker than olmutinib	Preclinical: in vitro
**95**	EGFR^T790M^; EGFR^L858R/T790M^	Kinase IC_50_ = 65.0 nM (T790M) and 13.6 nM (L858R/T790M); EGFR^WT > 1 μM; H1975 IC_50_ = 699.2 nM	Preclinical: in vitro
**100**	EGFR^L858R/T790M^	Mutant EGFR IC_50_ = 0.11 μM vs. WT EGFR = 12.43 μM; SI = 113	Preclinical: in vitro
**106**	EGFR^L858R/T790M^	Mutant EGFR IC_50_ = 13 nM vs. WT = 996 nM; 76.6-fold selectivity; H1975 IC_50_ = 0.087 μM; LO2 > 40 μM	Preclinical: in vitro
**108**	EGFR; NCI-60 cancer panel	EGFR IC_50_ = 36.7 nM; GI_50_ values around 10 nM for several sensitive leukemia, CNS and NSCLC lines	Preclinical: in vitro
**115**	EGFR; MiaPaCa2, DU145;	IC_50_ = 9.04 μM (MiaPaCa2) and 21.05 μM (DU145); EGFR inhibition was weak (~9.8% at 10 μM)	Preclinical: in vitro
**118**	EGFR^T790M/L858R^	Mutant EGFR IC_50_ = 4.90 nM vs. WT = 359.33 nM; SI = 73.33; H1975 IC_50_ = 0.62 μM; reduced toxicity toward L02/HBE cells	Preclinical: in vitro
**126**	PI3Kα	PI3Kα IC_50_ = 0.039 μM; MDA-MB-453 IC_50_ = 0.93 μM; HEK-293T IC_50_ = 28.18 μM	Preclinical: in vitro
**131**	PI3Kδ	PI3Kδ IC_50_ = 1.1 nM; PI3Kα/β/γ = 84/137/185 nM; ~76-fold δ/α selectivity and >100-fold vs. β/γ	Preclinical: in vitro
**134**	PI3Kα	92.4% PI3Kα inhibition at 1 μM; 62.3% PI3Kβ inhibition; HCT116 IC_50_ = 3.24 μM; not clearly PI3Kα-selective	Preclinical: in vitro
**136**	PI3Kα	PI3Kα IC_50_ = 0.20 μM; HCT-116 IC_50_ = 0.22 μM	Preclinical: in vitro
**143**	PI3Kα; H460, HT-29, MKN-45, MDA-MB-231	PI3Kα IC_50_ = 0.13 μM; cellular IC_50_ = 0.058–0.23 μM	Preclinical: in vitro
**145**	PI3Kδ	PI3Kδ pIC_50_ = 9.1; 126-, 158-, and 631-fold selectivity over PI3Kα, PI3Kβ, and PI3Kγ, respectively	Preclinical: in vitro
**149**	PI3K	Potent PI3K-pathway cellular activity; BBB penetration, transporter efflux and metabolic stability; PI3KC2β inhibition in kinase profiling	Preclinical: in vitro, in vivo optimization
**152**	Tubulin, colchicine-binding site	Tubulin polymerization IC_50_ = 2.54 ± 0.12 μM; cancer cell IC_50_ = 0.38–11.69 nM; direct colchicine-site binding confirmed crystallographically	Preclinical: in vitro
**157**	Tubulin, colchicine-binding site	A549/A2780/SKOV3/HCC827 IC_50_ = 0.84/0.38/0.31/0.34 nM; A2780/T RI = 1.97; tubulin K_D = 24.37 μM, retaining activity in paclitaxel-resistant cells	Preclinical: in vitro
**159**	Tubulin, colchicine-binding site; HL-60, HCT116, A549, MCF-7, HeLa	Tubulin polymerization IC_50_ = 4.1 μM; Antiproliferative IC_50_ = 34–196 nM; late apoptosis in HeLa reached 78.7% at 500 nM	Preclinical: in vitro
**163**	c-Met; A549, HepG2, MCF-7	c-Met IC_50_ = 16 nM; cellular IC_50_ = 0.9 μM (A549), 0.5 μM (HepG2), 1.1 μM (MCF-7); Flt-3 = 2800 nM, VEGFR-2 = 8900 nM, c-Kit/EGFR > 10,000 nM	Preclinical: in vitro
**169**	c-Met; A549, HepG2, MCF-7	c-Met IC_50_ = 19 nM; cellular IC_50_ = 0.58, 0.47, and 0.74 μM, respectively	Preclinical: in vitro
**173**	ATR; LoVo, HT-29	ATR IC_50_ = 1.5 nM; LoVo IC_50_ = 0.073 μM; HT-29 IC_50_ = 0.161 μM and 0.003 μM with AZD1390; good selectivity across an 80-kinase panel	Preclinical: in vitro, PK, in vivo xenograft; TGI = 55% at 50 mg/kg b.i.d.
**180**	PC3, MCF-7, Colo-205, A549; ATR proposed by molecular docking	IC_50_ = 0.012 μM (PC3), 0.019 μM (MCF-7), 0.11 μM (Colo-205), 0.87 μM (A549)	Preclinical: in vitro, in silico
**187**	VEGFR-2/KDR	VEGFR-2 inhibition = 67% at 10 μM; IC_50_ = 2.6 μM; inhibition of the other five kinases in the six-kinase panel remained < 50%	Preclinical: in vitro
**192**	VEGFR-2/KDR; HUVEC angiogenesis	VEGFR-2 IC_50_ = 2.25 ± 0.10 μM; 99.2% inhibition at 200 μM; endothelial tube-formation EC_50_ ≈ 31 nM vs. sunitinib 645 nM	Preclinical: biochemical, cellular/in vitro anti-angiogenic
**196**	CDK7/Cyclin H/MAT1; MDA-MB-453 TNBC	CDK7 IC_50_ = 1.4 nM; 100% inhibition at 100 and 1000 nM; MDA-MB-453 EC_50_ = 0.20 μM. In a 416-kinase panel at 100 nM, CDK7 inhibition was 98%; CLK3 was also strongly inhibited (99%), with ~14 additional kinases inhibited by 50–90%; Normal-cell EC_50_ = 9.7 μM (HEK293) and 8.64 μM (MCF-10A).	Preclinical: in vitro, kinome profiling, PK
**199**	CDK7/CyclinH/MAT1; MDA-MB-453, MDA-MB-231 TNBC	CDK7 IC_50_ = 6.5 ± 0.3 nM; EC_50_ = 0.15 μM (MDA-MB-453) and 0.32 μM (MDA-MB-231); 468-kinase profiling gave S(10) = 0.006; MAP2K5 IC_50_ = 170 nM	Preclinical: in vitro, kinome profiling, PK, in vivo xenograft; oral 5–10 mg/kg produced marked tumor regression
**205**	HDAC1, HDAC3 and HDAC6; RPMI 8226, HCT116	HDAC1/3/6 IC_50_ = 29.81 ± 0.52/24.71 ± 1.16/21.29 ± 0.32 nM; RPMI 8226 and HCT116 IC_50_ = 0.97 ± 0.072 and 1.01 ± 0.033 μM, respectively. Activity is pan-HDAC rather than isoform-selective; 0.3 μM increased acetylated histone H3 and α-tubulin.	Preclinical: in vitro
**209**	HDAC; HCT-116, HeLa, MCF-7	HDAC IC_50_ = 0.38 μM; HCT-116/HeLa/MCF-7 IC_50_ = 4.0/4.0/4.8 μM; 76.8% HDAC inhibition at 1 μM	Preclinical: in vitro
**213**	JAK1; H1975, H2087 NSCLC	JAK1 IC_50_ = 0.022 μM (22 nM); JAK2 = 0.759 μM, JAK3 = 1.601 μM; 35-fold selectivity over JAK2 and 73-fold over JAK3; H1975/H2087 GC_50_ = 6.67/5.96 μM	Preclinical: in vitro, kinase-selectivity profiling
**221**	JAK3; Ramos, Raji, Namalwa B-cell lymphoma	JAK3 IC_50_ = 1.89 ± 0.04 nM; BTK > 100 nM; Ramos/Raji/Namalwa IC_50_ = 10.44/9.43/9.92 μM; L-02 = 23.44 μM; minimal effect on PBMCs at 20 μM	Preclinical: in vitro
**225**	mTOR; MCF-7, PC-3, A549, MDA-MB-231 and breast cancer models	mTOR IC_50_ = 1.7 nM; PI3Kα ≈ 242 nM; 142-fold preference for mTOR; cellular IC_50_ = 0.08–1.14 μM	Preclinical: in vitro
**228**	PI3K/mTOR; PC-3, HCT-116, A549, MDA-MB-231	PI3Kα/β/γ/δ IC_50_ = 0.46/55/13/32 nM; mTOR = 12 nM; cellular IC_50_ = 1.95/2.02/1.62/1.55 μM	Preclinical: in vitro, in silico ADME; predicted oral bioavailability score 0.17
**230**	PI3Kα/δ and mTOR; SKOV3, Rh30, PC-3	PI3Kα/β/γ/δ/mTOR=12.5/70.2/276.3/47.2/12.5 nM. Cellular IC_50_=0.15–0.26 μM	Preclinical, in vitro, in vivo, PI3Kα co-crystal structure
**235**	PI3Kα/mTOR; H460, HT-29, MKN-45, MDA-MB-231	PI3Kα IC_50_ = 27 nM; mTOR = 0.77 μM; cellular IC_50_ = 0.039–0.25 μM; apoptosis demonstrated in HT-29	Preclinical: in vitro
**237**	Class I PI3K/mTOR; PC-3, U87MG, SKOV-3	PI3Kα/β/γ/δ IC_50_ = 15/175/75/10 nM; mTOR = 16 nM; submicromolar cellular activity	Advanced preclinical: in vitro, PK, in vivo xenograft
**238**	Class I PI3K—pictilisib (GDC-0941); PI3K-driven tumors	p110α IC_50_ = 3 nM; U87MG/A2780 IC_50_ = 0.95/0.14 μM; pAKT IC_50_ = 46/37/28 nM in U87MG/PC3/MDA-MB-361; strong selectivity over non-PI3K kinases	Clinical development: orally active, with in vivo U87MG antitumor efficacy
**239**	Pan-PI3K/mTOR—GNE-493; PC3, MCF7.1	PI3Kα/β/δ/γ/mTOR IC_50_ = 3.4/12/16/16/32 nM; PC3 ≈ 330 nM, MCF7.1 ≈ 180 nM	Advanced preclinical: PK, in vivo PD, xenograft efficacy
**240**	Pan-PI3K—GNE-490; PC3, MCF7.1	PI3Kα/β/δ/γ IC_50_ = 3.5/25/5.2/15 nM; mTOR = 740 nM; PC3 ≈ 490 nM, MCF7.1 ≈ 280 nM	Advanced preclinical: PK, in vivo PD, xenograft efficacy
**245**	PI3K/mTOR—apitolisib (GDC-0980)	PI3Kα/β/δ/γ IC_50_ = 5/27/7/14 nM; mTOR K_i_ = 17 nM; oral F ≈ 56–66%	Clinical development: tumor stasis/regression demonstrated in PC-3 and MCF-7 neo/HER2 xenografts
**249**	EGFR/ErbB-2	EGFR IC_50_ = 1 nM; ErbB-2 IC_50_ = 71 nM	Preclinical: in vitro
**252**	Covalent EGFR/ErbB-2; BT474	EGFR IC_50_ = 32 nM; ErbB-2 = 20 nM; BT474 IC_50_ = 20 nM; ErbB-2 cellular DELFIA IC_50_ = 10 nM	Preclinical: in vitro, mouse oral PK; poor oral exposure for this stereoisomer
**256**	Tubulin/EGFR dual targeting; A549, HeLa, HT-29, Jurkat	Cellular IC_50_ = 1–20 nM; tubulin polymerization IC_50_ = 0.71 μM; EGFR IC_50_ ≈ 30 nM	Advanced preclinical: in vitro, in vivo xenograft; TGI ≈ 52.5% at 7.5 mg/kg
**268**	EGFR^L858R/T790M^ / PI3Kα; NCI-H1975 NSCLC	EGFR^L858R/T790M^ IC_50_ = 3.6 nM; PI3Kα = 30 nM; PI3Kβ = 450.5 nM; PI3Kδ/γ/mTOR > 1 μM; NCI-H1975 IC_50_ = 0.48 μM vs. A549 > 10 μM (SI > 20.8)	Preclinical: in vitro, metabolic stability
**272**	ATR/PIKK family—predicted by molecular docking; HCT116, HeLa	HCT116 IC_50_ = 7.164 μM; HeLa = 4.799 μM; computational docking suggested ATR/PIKK interactions	Early preclinical: in silico, in vitro cellular
**274**	PI3Kδ/BRD4-BD1; SU-DHL-4, SU-DHL-6 DLBCL	PI3Kδ IC_50_ = 112 ± 8 nM; BRD4-BD1 = 19 ± 1 nM; SU-DHL-4/SU-DHL-6 IC_50_ = 91/61 nM	Advanced preclinical: oral PK, xenograft; F = 74.8%, TGI = 70.8% at 20 mg/kg
**281**	PARP-1/PI3Kα; BRCA-deficient and BRCA-proficient cancer cells	PARP-1 = 1.57 nM; PARP-2 = 0.89 nM; PI3Kα/β/γ/δ = 2.0/53/25/6.8 nM; mTOR = 50 nM; cellular IC_50_ = 0.31–1.30 μM	Advanced preclinical: in vitro, MDA-MB-468 xenograft; TGI = 56.39%
**283**	PARP-1/PI3Kα; BRCA-deficient and BRCA-proficient cancer cells	PARP-1 = 0.91 nM; PARP-2 = 0.92 nM; PI3Kα/β/γ/δ = 1.5/43/73/2.7 nM; mTOR = 12 nM; cellular IC_50_ = 0.28–1.02 μM; hERG > 30 μM	Advanced preclinical: in vitro, MDA-MB-468 xenograft; TGI = 48.77%
**288**	JAK3/BTK; biological validation primarily in pulmonary fibrosis	JAK3 IC_50_ = 1.38 nM; BTK = 62.4 nM; normal HBE IC_50_ = 39 μM	Preclinical: in vitro, in vivo pharmacology in a non-oncology BLM-induced fibrosis model
**294**	Pan-PI3K/mTOR/PIM—IBL-302/AUM302; MV4:11 and solid-tumor models	PI3Kα/β/δ/γ K_i_ = 0.27/0.31/0.13/0.17 nM; mTOR IC_50_ ≈ 74.5 nM; PIM-1/2/3 IC_50_ = 22.8/8.04/5.75 nM; pAKT EC_50_ < 1 nM; pBAD = 60 nM	Late preclinical development: oral PK, in vivo xenograft efficacy
**298**	FAK; U-87MG, A549, MDA-MB-231	FAK IC_50_ = 28.2 nM; U-87MG/A549/MDA-MB-231 IC_50_ = 0.16/0.27/0.19 μM; HK2 normal cells IC_50_ = 3.32 μM	Preclinical: in vitro
**300**	Apoptosis/caspase-9; MCF-7	MCF-7 IC_50_ = 0.43 μM; G2/M population increased from 18.7 to 49.1%; apoptosis from 2.15 to 29.24%; caspase-9 activation	Preclinical: in vitro
**304**	STK17B—SGC-STK17B-1	STK17B biochemical IC_50_ = 34 nM; cellular NanoBRET IC_50_ = 190 nM; K_D = 5.6 nM; >100-fold selectivity over STK17A; broad kinome profiling	Preclinical chemical probe: biochemical, cellular target engagement
**307**	Pan-TRK (TRKA/TRKB/TRKC); KM12	TRKA/TRKB/TRKC IC_50_ = 75/74/55 nM; KM12 IC_50_ = 0.14 μM	Preclinical: in vitro, kinase profiling, BBB permeability assay
**314**	SIRT3; MOLM13, MV4-11, HL-60 AML	SIRT3 IC_50_ = 0.043 μM; SIRT1/SIRT2 = 0.387/0.474 μM (~9-/11-fold selectivity); MOLM13/MV4-11/HL-60 IC_50_ = 0.32/0.73/6.21 μM	Advanced preclinical: in vitro, in vivo AML model
**318**	Cdc7/Dbf4; COLO205	Cdc7 IC_50_ = 0.70 nM; CDK2/cyclin E > 10 μM; ROCK1 = 140 nM; > 14,000-fold selectivity over CDK2; COLO205 growth IC_50_ = 1.1 μM; pMCM2 PD EC_50_ = 250 nM	Preclinical: biochemical, cellular PD
**323**	PRMT7; prostate cancer (DU-145, 22Rv1)	PRMT7 IC_50_ = 0.50 μM; K_D = 0.32 μM; PRMT1/3/4/5/6/8/9 IC_50_ > 100 μM; PCa-cell IC_50_ ≈ 0.5–0.7 μM	Advanced preclinical: in vitro, DU-145 xenograft; tumor inhibition 62.4%; low oral bioavailability
**326**	BTK; B-cell proliferation	BTK IC_50_ = 29.9 nM; EGFR > 10 μM; B-cell proliferation IC_50_ = 284 nM; T-cell IC_50_ > 10 μM; CC_50_ > 50 μM	Preclinical: in vitro biochemical, cellular profiling
**331**	FAK/FLT3 mutants; HCT-116, MDA-MB-231, MV4-11	FAK IC_50_ ≈ 7–9.7 nM; FLT3-D835Y < 0.5 nM; strong activity against FLT3-ITD and resistance-associated mutants; multitarget kinome profile	Advanced preclinical: PK, multiple in vivo tumor models
**333**	Pan-BET (BRD2/3/4/BRDT); SU-DHL-4, Jurkat	BRD2-BD1/BRD3-BD1/BRD4-BD1/BRD4-BD2/BRDT-BD1 IC_50_ = 4.1/20.4/3.4/3.3/42.0 nM; SU-DHL-4/Jurkat IC_50_ = 8.6/52.3 nM	Preclinical: in vitro, in silico, ADME/physicochemical profiling
**338**	DNA demethylation; DNMT1/BAH2 proposed by docking; HeLa	Tumor-DNA methylation reduced by 89.1%; HeLa IC_50_ = 4.4 ± 0.3 μg/mL, Vero = 6.4 ± 0.4 μg/mL	Early preclinical: in vitro, in silico docking
**341**	CDK-associated mechanism proposed by docking; HeLa, HT-29	HeLa IC_50_ = 0.591 μM (72 h); G2/M arrest, ROS generation, microtubule disruption and apoptosis; docking to CDK-related targets	Early preclinical: in vitro cellular, in silico docking

## Data Availability

No new data were created or analyzed in this study. Data sharing is not applicable to this review article.
